# Calix[4]Resorcinarene Carboxybetaines and Carboxybetaine Esters: Synthesis, Investigation of In Vitro Toxicity, Anti-Platelet Effects, Anticoagulant Activity, and BSA Binding Affinities

**DOI:** 10.3390/ijms232315298

**Published:** 2022-12-04

**Authors:** Julia E. Morozova, Zuchra R. Gilmullina, Alexandra D. Voloshina, Anna P. Lyubina, Syumbelya K. Amerhanova, Victor V. Syakaev, Olga B. Babaeva, Albina Y. Ziganshina, Timur A. Mukhametzyanov, Aleksandr V. Samorodov, Michael M. Galagudza, Igor S. Antipin

**Affiliations:** 1 Arbuzov Institute of Organic and Physical Chemistry, FRC Kazan Scientific Center of RAS, 8 Arbuzov Street, 420088 Kazan, Russia; 2 Alexander Butlerov Chemistry Institute, Kazan Federal University, 18 Kremlyovskaya Str., 420008 Kazan, Russia; 3 Department of Pharmacology, Bashkir State Medical University, 3 Lenina Str., 450008 Ufa, Russia; 4 Almazov National Medical Research Centre, 190000 Saint Petersburg, Russia

**Keywords:** calix[4]resorcinarene, carboxybetaine, hemotoxicity, cytotoxicity, anti-platelet effect, anticoagulant activity, bovine serum albumin

## Abstract

As a result of bright complexation properties, easy functionalization and the ability to self-organize in an aqueous solution, amphiphilic supramolecular macrocycles are being actively studied for their application in nanomedicine (drug delivery systems, therapeutic and theranostic agents, and others). In this regard, it is important to study their potential toxic effects. Here, the synthesis of amphiphilic calix[4]resorcinarene carboxybetaines and their esters and the study of a number of their microbiological properties are presented: cytotoxic effect on normal and tumor cells and effect on cellular and non-cellular components of blood (hemotoxicity, anti-platelet effect, and anticoagulant activity). Additionally, the interaction of macrocycles with bovine serum albumin as a model plasma protein is estimated by various methods (fluorescence spectroscopy, synchronous fluorescence spectroscopy, circular dichroic spectroscopy, and dynamic light scattering). The results demonstrate the low toxicity of the macrocycles, their anti-platelet effects at the level of acetylsalicylic acid, and weak anticoagulant activity. The study of BSA–macrocycle interactions demonstrates the dependence on macrocycle hydrophilic/hydrophobic group structure; in the case of carboxybetaines, the formation of complexes prevents self-aggregation of BSA molecules in solution. The present study demonstrates new data on potential drug delivery nanosystems based on amphiphilic calix[4]resorcinarenes for their cytotoxicity and effects on blood components.

## 1. Introduction

Supramolecular macrocycles are multifunctional platforms, which, with appropriate functionalization, can act as drug delivery systems [1,2], diagnostic systems [3], antimicrobials [4], antidotes [5], and other therapeutic agents [6,7]. With regard to the creation of drug delivery systems, such popular supramolecular macrocycles as calix[n]arenes and their analogues, calix[4]resorcinarenes, occupy a special niche. The ease of synthesis and functionalization of these macrocycles, their ability to participate in various non-covalent interactions, bright receptor properties, and the ability, with suitable modification, for spontaneous self-organization in aqueous solutions have led to a large number of works devoted to their potential biomedical applications [1,2,3,4,5,6,7,8]. Calixarenes in the cone and calix[4]resorcinarenes in the cone and boat conformations with aliphatic substituents in the lower rim and hydrophilic substituents in the upper rim are amphiphilic macrocycles capable of forming co-associates with drugs and targeting ligands in solutions, which promotes their use as drug delivery systems [9,10].

When developing new drug delivery systems, in particular for cancer treatment, intravenous injection for most nanosystems is intended. As a rule, the in vitro cytotoxic effects of nanosystems on normal and cancer cells are evaluated first of all. However, intravenous administration of nanosystems can lead to problems already in the vascular system [9]. Blood is a complex biological fluid composed of red blood cells, white blood cells, platelets, and plasma fluid, which in turn is a highly concentrated solution of proteins, blood clotting factors, sugars, and electrolytes. Once in the bloodstream, the nanosystem can come into contact with blood components. Thus, when creating potential nanosystems and their constituent parts, it is necessary to evaluate their effects on the blood components [11].

One of the problems associated with nanosystems in the blood is the ability to cause erythrocyte destruction, which can be determined by assessing the hemolytic activity of the system. Further, in addition to the activation of leukocytes (which can cause an immune response), nanoscale systems can induce the activation of platelets, affecting the processes of homeostasis. It has been previously shown that the presence of nanosystems can disrupt the coagulation system and cause hemostatic imbalance, which can lead to serious life-threatening conditions, such as venous thrombosis [12].

The non-cellular component of blood, plasma, also plays an important role in intravenous drug delivery, especially in the uptake and clearance of particles by the reticuloendothelial system. Therefore, the assessment of the interaction of nanosystems with the most common blood proteins, such as serum albumins, is an important aspect. Albumins perform a number of important functions in the body (regulation of blood pH and osmotic pressure, binding and transport of ions, metabolites, and harmful substances [13]). The characteristics of the structural domains of albumin allow it to bind to biologically active compounds and metal nanoparticles. The protein consists of both hydrophilic and hydrophobic zones, allowing it to strongly adhere to surfaces, where it then serves as an anchor layer for the adhesion of other proteins [13]. The functionality of BSA has led to its study as a component of nanosystems for drug transport and metal nanoparticle stabilization [14].

Here, we present new amphiphilic carboxybetaines, calix[4]resorcinarenes and their ester precursors, and carry out an in vitro study of their effects on blood components—erythrocyte hemolysis, plasma clotting, and anti-platelet action. The study of their interactions with a model blood plasma protein, BSA, is also presented. The data on their cytotoxic effects on normal and tumor cells are shown as well.

In the literature, there are scattered data on the study of the effects of water-soluble calix[n]arenes and calix[4]resorcinarenes on blood components. It was previously shown that para-sulfonatocalix[n]arenes have almost no hemolytic effect at concentrations below 2 × 10^−1^ M [15]. The high hemolytic activity was observed with the use of amino derivatives of calix[4]arenes [16] and calix[4]resorcinarenes [17], as well as calix[4]resorcinarene cavitands with lipophilic cationic groups [18]. Calix[4]resorcinarene bearing N-methyl-D-glucamine groups [19] and pegylated calix[4]resorcinarenes [20,21,22,23] has zero or low effect on red blood cells. It was found that the hemolytic activity of carboxy calix[4]resorcinarenes depends on the hydrophobicity of macrocycles: basically, macrocycles demonstrate low hemolysis (0.34–3.42% at C = 5 × 10^−4^ M) except for the macrocycle with octyl groups on the lower rim (34.1% at C = 5 × 10^−4^ M) [24].

It was earlier reported that para-sulfonatocalix[8]arenes and calix[8]arenes, bearing propanosulfonic and butanosulfonic groups, pose potential anticoagulant and antithrombotic effects [8,25,26]. The anticoagulant behavior of O-derivatized para-octanoyl-calix[8]arenes bearing carboxymethoxy and 4-sulfonatobutoxy groups has been shown in ref. [27]. Calix[4]arene tetrakis-methylene-bis-phosphonic acid and its sodium salt have antithrombotic properties, and act as inhibitors of blood coagulation and fibrin polymerization [28,29].

It was shown that in the solution, the interaction of para-sulfonatocalix[n]arenes with BSA led to the aggregation of protein molecules [15]. Oppositely, amphiphilic thiacalixarenes modified with sulfobetaine groups stabilize the monomeric form of BSA in solution, due to the formation of complexes in which BSA molecules are wrapped around the macrocycle molecules [30]. It was reported that sulfonatomethylated tetrapentylcalix [4]resorcinarene interacts with BSA due to the hydrogen bonding and van der Waals forces, and lg*K*_as_ values of macrocycle–BSA complex are 6.76, 6.41, and 6.04 at 298, 303 and 308 K, respectively [31]. It was also shown that cationic bis-ammonium thiacalix[4]arenes form complexes with BSA, for which values of association constant increase with the growth of macrocycle hydrophobicity [32].

Thus, data in the literature show that calixarenes and calix[4]resorcinarenes both can have different effects on blood components, which implies that even small differences in the structure of the macrocycle can lead to a change in microbiological properties.

When a nanosystem enters the blood, protein is adsorbed on the surface of the particle in seconds [33], but it can be prevented by “invisible” “stealth” mechanisms. Potential ways to limit the effects of plasma protein targeting include the use of pegylation and zwitterionic functionalization of the particle surface, which can limit protein adsorption and thus increase in vivo circulation time and prevent non-specific interactions with the immune system [34,35]. Nevertheless, the functionalization of amphiphilic calix[4]resorcinarenes with the potential for hydrophobic interactions with protein molecules by zwitterionic groups raises questions about the prediction of possible complex formation with plasma proteins.

**Scheme 1:**
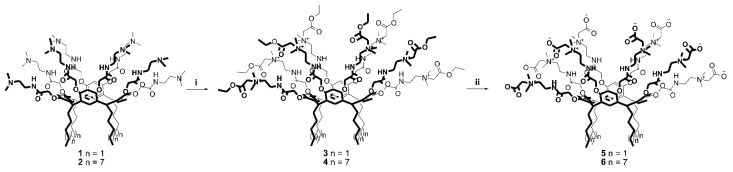
The synthetic path for macrocycles **3**–**6**. Reagents and conditions: (i) ethyl bromoacetate, dry acetonitrile, 60 °C, 44 h; (ii) NaOH, ethanol, 60 °C, 23 h.

Our work is aimed at the study of a number of microbiological and BSA-binding properties of calix[4]resorcinarene carboxybetaine esters and carboxybetaines (Scheme 1). The paper considers four compounds with varying lengths of aliphatic substituents—pentyl and undecyl, and varying hydrophilic peripheral groups—cationic and zwitterionic. The study shows that if the hemolysis practically does not depend on the structure of these macrocycles, then cytotoxicity is more pronounced in the ether derivatives. Additionally, the interaction with BSA is stronger with ester macrocycles, and anti-platelet effects and anticoagulant activity are practically independent of the structure of the macrocycles.

## 2. Results and Discussion

### 2.1. Synthesis of the Calix[4]Resorcinarene Carboxybetaines and Carboxybetaine Esters

To prepare calix[4]resorcinarene carboxybetaines, two calix[4]resorcinarenes in boat conformation with N-(2-(dimethylamino)ethyl)-2-methoxyacetamide groups on the upper rim and pentyl (**1**) and undecyl groups (**2**) on the lower rim were used (Scheme 1). The peripheral dimethylamino-groups of macrocycles **1** and **2** were treated with ethyl ester of bromoacetic acid to obtain cationic ester macrocycles **3** and **4**; in the next step, the saponification with sodium hydroxide led to carboxybetaines **5** and **6**. The compounds **3**–**6** were characterized by ^1^H and ^13^C NMR spectra, IR spectra, ESI mass spectra, and elemental analysis data. The spectral data of macrocycles confirmed the completeness of their substitution (Figures S13–S46). In the ^1^H NMR spectra, the quaternization of dimethylamino groups of initial macrocycles **1** and **2** led to a downfield shift of corresponding signals to about 3.3 ppm, and the saponification led to the disappearance of ester group signals at about 1.2–1.3 and about 4.2–4.3 ppm. It should be noted that, as a rule, the functionalization of calix[4]resorcinarene OH-groups by bulky substituents leads to the broadening of some signals in the NMR spectra for a number of reasons: the slowing down of the intramolecular mobility of substituents, the influence of magnetically anisotropic groups and different orientation of substituents relative to them in different spatial forms. In IR spectra, the greatest change was observed in the region of 1700–1500 cm^−1^ (stretching vibrations of C=O groups, and Amide I and Amide II absorption bands) and about 1300 cm^−1^ (C-O-C group stretching vibrations).

The boat conformation of the calix[4]resorcinarene molecule, functionalized by hydrophobic substituents on the lower rim and hydrophilic groups on the upper rim, suggests the clear separation of the hydrophobic and hydrophilic zones in the macrocycle molecule and its amphiphilic properties. Variation in the length of hydrophobic substituents of calix[4]resorcinarenes affects their properties: an increase in hydrophobicity leads to an increase in the size of self-associates and an increase in the binding of guest molecules due to their inclusion in co-associates [36,37].

The amphiphilic nature of the macrocycles was confirmed by pyrene fluorescence (Figure S47). The critical association concentration (cac) values were 5.8 × 10^−4^, 2.2 × 10^−4^, 3.9 × 10^−4^, and 2.7 × 10^−5^ M for macrocycles **3**, **4**, **5**, and **6**, respectively. A decrease in cac values was observed with an increase in the hydrophobicity of lower rim substituents of macrocycles, and also when cationic ester groups of the upper rim were changed to zwitterionic, the latter indicating the increase in the hydrophobicity of self-associates of zwitterionic macrocycles. According to data in the literature, zwitterionic and, in particular, carboxybetaine groups effectively interact with water molecules [38], which probably leads to an increase in hydrophobic interactions between adjacent molecules of macrocycles in their self-associates.

The ability of macrocycles **3**–**6** to form self-associates in solution can be used to create supramolecular nanosystems on their base. Independent of a pathway for a nanosystem entering the body (skin, esophagus, lungs, intravenous injection), it will eventually enter the bloodstream. After entering the systemic circulation, the nanosystem collides with various blood components, including blood cells and a number of serum proteins. These components are by no means inert with respect to interaction with nanoobjects and can significantly affect their further existence in the body. Therefore, along with the study of the cytotoxicity of nanosystems, it is necessary to study their hemolytic activity and interaction with blood components involved in hemostasis.

In this regard, the microbiological activity of calix[4]resorcinarenes **3**–**6** was studied, which included (1) an assessment of cytotoxicity against normal (Chang liver cells) and cancer cells (M-HeLa cells), (2) a test for toxicity against human blood erythrocytes (hemolytic activity), (3) test for anticoagulant activity, (4) test for aggregation activity against human blood platelets, and (5) a study of interaction with a model plasma protein, BSA.

### 2.2. The Cytotoxic Effect of Macrocycles ***3***–***6*** on Human Cell Lines

In vitro assessment of macrocycles’ cytotoxicity was performed on popular test cell cultures, normal Chang liver cells cells and M-HeLa tumor cells, by counting viable cells cultivated in the presence of macrocycles, using flow cytometry. According to the data obtained, zwitterionic macrocycles 5 and 6 showed less cytotoxicity than cationic ester macrocycles **3** and **4** (Table 1, Table S1). Interestingly, macrocycles **3** and **4** showed a higher cytotoxic effect on normal cells. The lowest toxicity was observed for the zwitterionic macrocycle 5 with pentyl lower rim substituents.

### 2.3. The Hemolytic Activity of Macrocycles ***3***–***6***

The hemolysis test evaluates the ability of the studied compound to cause the destruction of human red blood cells and illustrates its toxic effect on the internal environment of the body. There is no standard preclinical in vivo testing method to comprehensively evaluate the hemolytic response of pharmaceuticals, so the in vitro hemolytic activity test is taken into account in toxicity studies [39].

The toxic effects of the studied compounds are observed when the erythrocytes are destroyed with the release of hemoglobin, while the optical density of the liquid is controlled. Data on the hemolytic activity of macrocycles **3**–**6** are presented in Table 1; in the studied concentration range, the HC_50_ value was not reached. In a 5 mM solution of macrocycles, the hemolysis ranged from 0 to 8% (Table S2), which demonstrated the low hemolytic activity of the compounds.

### 2.4. Plasma Clotting Assessment

The coagulation balance of blood in the body is achieved by the interaction of platelets with the plasma coagulation system and vascular endothelial cells. In a healthy body, these systems prevent blood clots, and when blood vessels are damaged, they contribute to blood clotting to stop bleeding. Dysregulation of hemostasis can cause serious thrombotic and/or hemorrhagic pathologies [40].

In plasma coagulation experiments, the values of activated partial thromboplastin time (APTT), prothrombin time (PT), and plasma fibrinogen (Fib) are most commonly used. PT and APTT reflect the state of the exogenous and endogenous blood coagulation system, respectively. After adding drugs to the plasma, the appearance of a gel-like clot is recorded using a coagulometer, and the APTT and PT are measured and compared with normal groups containing an equivalent concentration of saline [39].

The data obtained are presented in Table 2. Macrocycles **3**–**6** affected only APTT values (the internal pathway of blood coagulation). Fibrinogen concentration and prothrombin time did not change (data not shown). The APTT assay measures the clotting factors present in the intrinsic clotting system and is commonly used to monitor therapy with the known anticoagulant heparin. All compounds caused an increase in APTT by 3.1–8.4% (hypocoagulation) compared with the control; however, the effects obtained were significantly inferior to those of heparin, which increases APTT by 20.3%. The zwitterionic macrocycle **5** lowered coagulation most strongly, exhibiting a weak anticoagulant activity.

### 2.5. Platelet Aggregation Test

Platelets in the human circulation are dispersed, and their activation and aggregation are one of the risk factors in the pathogenesis of thrombosis and atherosclerosis [41]. Thus, drugs intended for intravenous administration should not cause platelet aggregation.

Platelet aggregation induced by agonists such as adenosine diphosphate (ADP), collagen, and thrombin is an important indicator of platelet function in vitro. The assessment of aggregation parameters is usually carried out by the turbidimetric method described by Born (1962) [42]: the effect on aggregation parameters is compared with known inhibitors of platelet aggregation, for example, with acetylsalicylic acid (AA).

The effects of macrocycles **3**–**6** on the maximum amplitude (MA), the rate of aggregation, and the time to reach MA in ADP-induced platelet aggregation were studied. The results are shown in Table 2. All tested compounds inhibited platelet aggregation. According to the MA values, macrocycles exhibited platelet antiaggregatory ability at the level of AA. Moreover, zwitterionic macrocycle 6 reduced the aggregation rate value and increased the time to reach MA more efficiently than acetylsalicylic acid.

Thus, macrocycles **3**–**6** show low toxicity on human erythrocytes and a low ability to influence the plasma component of the hemostasis system, but they can lead to inhibition of platelet aggregation at the AA level, which should be taken into account when developing drug delivery systems based on them.

### 2.6. The Study of the Interaction of Macrocycles ***3***–***6*** with Bovine Serum Albumin

Understanding the interaction of nanosystems with proteins in plasma is an important factor facilitating their application in the field of biomedicine. Serum protein adsorption can affect the internalization and biodistribution of nanosystems, so the interaction between plasma proteins and nanomaterials needs to be evaluated. As known, serum albumins are the main endogenous proteins in blood circulation; they constitute about 60% of the total protein species of blood and play an important role in the transportation of exogenous substances. Due to the sequence similarity between HSA and BSA, the latter is used as the model protein for diverse physicochemical and biochemical studies [43].

To establish the interaction of macrocycles **3**–**6** with BSA, parameters of complex formation were estimated (fluorimetry), and the effect of macrocycles on protein conformation (circular dichroism and synchronous fluorimetry), and the self-aggregation of protein molecules in solution (dynamic light scattering (DLS)) were studied.

#### 2.6.1. The Study of Macrocycle–BSA Interactions by Fluorimetry

Due to the presence of amino acid residues (tryptophan, tyrosine, and, to a lesser extent, phenylalanine), BSA exhibits fluorescent properties. When a compound interacts with BSA, the protein fluorescence intensity is quenched, which makes it possible to establish the mechanism of interaction and to find quantitative characteristics (quenching and binding constants and thermodynamic parameters) by varying the quencher concentration and temperature [44].

The fluorimetry method showed that an increase in macrocycle concentration in solution leads to an increase in BSA emission quenching (Figure S48). To obtain BSA fluorescence spectra, the solutions were excited at 279 nm (BSA absorption maximum), and an emission maximum was recorded at 337 nm. Herewith, the BSA emission intensity can be underestimated due to the absorption of the quencher at both excitation and emission wavelengths. This is the so-called inner filter effect (IFE) [45], which should be taken into account by estimating the absorption intensity of solutions at the corresponding wavelengths and calculating the coefficient using the formula:

F_corr_ = F_exp_ × e^(Aem+Aex)/2^, (1)

where F_exp_ and F_corr_ are the experimental and corrected fluorescence intensity, respectively; A_em_ and A_ex_ are the absorbance at emission (337 nm) and excitation (279 nm) wavelength, respectively, of each solution.

The absorption spectra of BSA–macrocycle solutions were registered at different concentrations of macrocycles, and the dependences of the absorption intensity at 279 and 337 nm on the concentration of macrocycles were plotted (Figure S49). It was found that the change in intensity at 337 nm did not exceed 5%, so this value was neglected. From the plot A_279_(C), where C is concentration of macrocycle, the e^(Aex)/2^ coefficients were calculated. For subsequent calculations, the value of F_exp_, obtained at 337 nm, at each macrocycle concentration, was multiplied by the corresponding coefficient e^(Aex)/2^.

To study the BSA quenching process, the fluorescence spectra of BSA–macrocycle solutions were analyzed at three temperatures (293, 303, and 308 K). In all cases, with an increase in the concentration of macrocycles, the quenching of BSA fluorescence was observed (Figure S48). The dynamic quenching constant *K*_SV_ and the bimolecular quenching rate constant *k*_q_ were determined with the help of the Stern–Volmer equation:

F_0_/F = 1 + *K*_SV_[C] = 1 + *k*_q_τ_0_[C], (2)

where F_0_ and F are the intensity of BSA emission at 337 nm in the absence and presence of a quencher, C is the concentration of the quencher (macrocycle), τ_0_ is the mean lifetime of the molecule in the excitation state (τ_0_ = 1 × 10^−8^ s for BSA [46,47]).

**Figure 1:**
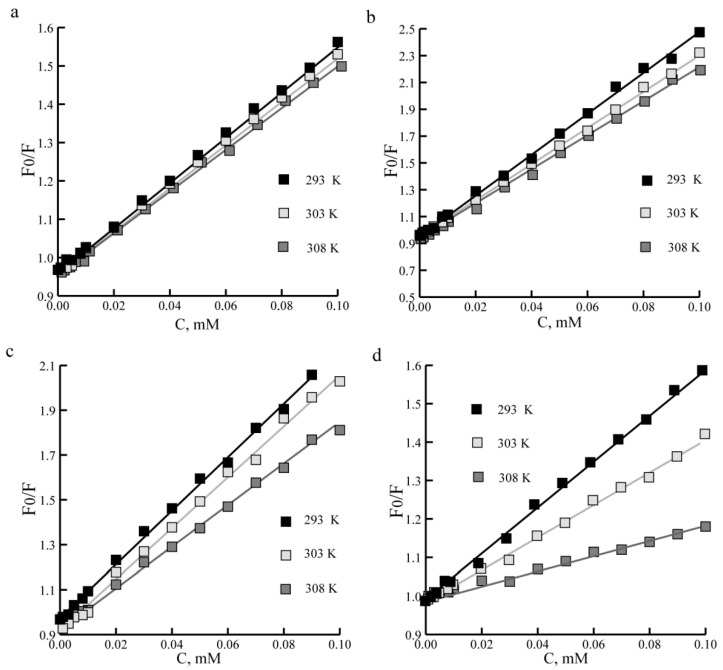
Stern–Volmer plots for BSA quenching by macrocycles **3** (**a**), **5** (**b**), **4** (**c**), and **6** (**d**) at three different temperatures.

The F_0_/F([C]) plots for macrocycles **3**–**6**–BSA solutions are given in Figure 1. The plots are arranged in such order that the plot obtained at a lower temperature is higher than the plot obtained at a higher temperature, which corresponds to the static quenching mechanism associated with the formation of the ground-state complex of BSA with macrocycles. The linearity of Stern–Volmer plots indicated the existence of one type of fluorophore in solution equally accessible to the quencher. The slope of the plots was equal to *K*_SV_ (Table 3). The *k*_q_ values were much higher than the *k*_q_ value for the dynamic quenching mechanism (2 × 10^10^ L mol^−1^s^−1^ [48]), which also confirmed the quenching mechanism due to the complex formation. The quenching constants for zwitterionic macrocycles were 2–5 times lower than for cationic esters **3** and **4**, which meant a weaker interaction of BSA with carboxybetaine macrocycles.

To evaluate the binding constant (*K*_as_) of fluorophore and the number of binding sites (n), the following equation was used:

lg(F_0_ − F)/F = lg*K*_as_ + n log[C], (3)

It describes the linear dependence lg((F_0_ − F_0_)/F)(logC), in which the slope determines the number of binding sites, and the segment on the y-axis determines lg*K*_as_. The plots lg((F_0_ − F_0_/F) (lgC) are presented in Figure S50, and the obtained data are presented in Table 3. The number of binding sites was close to 1. According to lg*K*_as_ values, cationic ester macrocycles **3** and **4** showed a higher ability to form complexes with BSA. It should be noted that at 308 K, complexes of BSA with zwitterionic macrocycles **5** and **6** had low lg*K*_as_ values [49]; thus, it can be expected that under physiological conditions, these macrocycles will weakly interact with the serum proteins.

Thermodynamic parameters of complexation (ΔH° and ΔS°) can be obtained using linear plot ln*K*_as_(1/T) according to the van ’t Hoff equation:

ln*K*_as_ = −ΔH°/RT + ΔS°/R, (4)

where T is the absolute temperature and R is the gas constant. Further, the value of the Gibbs energy (ΔG°) was calculated according to the following equation:

ΔG° = −ΔH° − TΔS°(5)

The plots ln*K*_as_(1/T) are presented in Figure S51, and the thermodynamic parameters obtained are presented in Table 3.

The negative ΔG° values reflect the spontaneous process of BSA–macrocycle binding. According to the literature, the sign and magnitude of ΔH° and ΔS° are associated with various types of intermolecular interaction; ΔH° > 0 and ΔS° > 0 suggest hydrophobic forces; ΔH° < 0 and ΔS° < 0 imply van der Waals forces and H-bonds; and ΔH° < 0 and ΔS° > 0 reflect electrostatic forces [50].

In the current study, the values of ΔH° and ΔS° were negative, which means H-bonds and van der Waals forces are the main driving forces of the binding process. As known, van der Waals forces reflect dipole–dipole interactions in supramolecular systems and affect the structure of proteins. Zwitterionic groups in macrocycles **5** and **6** and cationic groups in macrocycles **3** and **4** both can participate in dipole–dipole interactions with protein fragments, while for cationic ester macrocycles, there will also be a contribution from electrostatic interactions (since BSA has negative surface charge in solution at pH 7 [51]). The values of the thermodynamic parameters confirmed the stronger interaction of BSA with cationic ester macrocycles.

#### 2.6.2. Effect of Macrocycles **3**–**6** on BSA Conformation

Synchronous fluorescence spectroscopy is used to study protein conformational changes in the presence of quenching molecules. This method is based on simultaneous scanning of excitation and emission wavelengths, between which a constant interval (Δλ) is maintained. In the case of BSA, this method is used to study the surroundings of the tryptophan and tyrosine residues by measuring the shift in the position of the maximum. At low values of Δλ, the fluorescence spectrum of the tryptophan–tyrosine mixture is similar to the fluorescence spectrum of tyrosine (Δλ = 15 nm), and at large values, it is similar to the fluorescence spectrum of tryptophan (Δλ = 60 nm). The shift in the position of the band maximum in these spectra indicates a change in polarity near the chromophore fragment [52].

**Figure 2:**
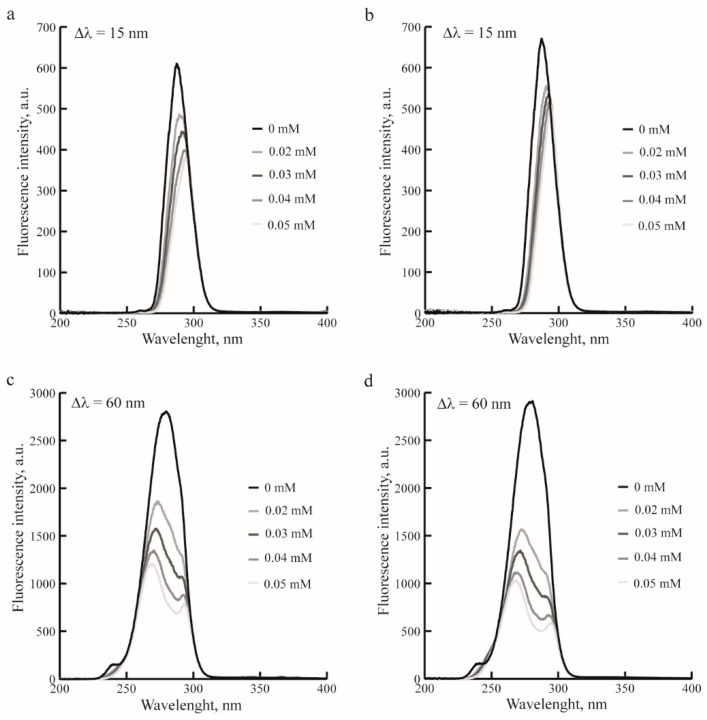
Synchronous fluorescence spectra of BSA in the presence of various amounts of macrocycles **3** (**a**,**c**) and **5** (**b**,**d**).

Cationic ester **3** and zwitterionic macrocycle **5** were chosen for the synchronous fluorescence experiment (Figure 2). It can be seen from the spectra that, in addition to the shift of the emission maximum, BSA fluorescence quenching is observed in the presence of macrocycles. Considering that quenching can be distorted due to IFE, the spectra of solutions were analyzed only at low concentrations of macrocycles (0.02–0.05 mM). In the spectra with Δλ = 15 nm, a red shift was observed, which indicated a decrease in the hydrophobicity of the microenvironment around tyrosine residues. In the spectra with Δλ = 60 nm, a blue shift was observed, which indicated an increase in the hydrophobicity of the microenvironment near tryptophan residues. Thus, the presence of macrocycles affected the microenvironment near the BSA amino acid residues and, thereby, changed its conformation.

**Figure 3:**
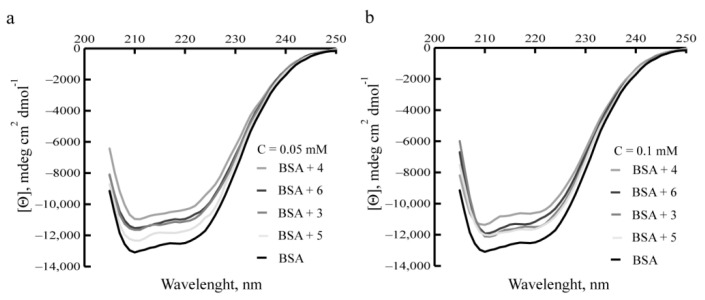
CD spectra of BSA in the presence of 5 × 10^−5^ M (**a**) and 1 × 10^−4^ M (**b**) solutions of macrocycles **3**–**6**.

Circular dichroism (CD) spectroscopy is used to establish the secondary structure of a protein and to determine its folding properties. BSA refers to α-helical proteins that have negative bands at 222 nm (n–π * transition) and 208 nm (π–π * transition) in the CD spectrum [53]. CD spectra of BSA were obtained in the presence of 5 and 10-fold excess of macrocycles **3**–**6** (Figure 3). In each case, a decrease in the intensity of negative peaks was observed, which indicated a decrease in protein α-helicity [53]. At the same time, at both concentrations, the same order of influence on the protein spectrum was observed: the intensity of peaks in the spectrum decreased most of all with tetraundecyl ether **4**, then tetraundecyl zwitterion **6**, then tetrapentyl ester **3**, and the presence of tetrapentyl zwitterion **5** had the least effect. Thus, it can be assumed that the interaction of macrocycles with BSA also included a hydrophobic effect due to the participation of alkyl groups of macrocycles, and an increase in the concentration of macrocycles in solution enhanced this effect.

#### 2.6.3. The Study of BSA–Macrocycle Solutions by Dynamic Light Scattering Method

**Figure 4:**
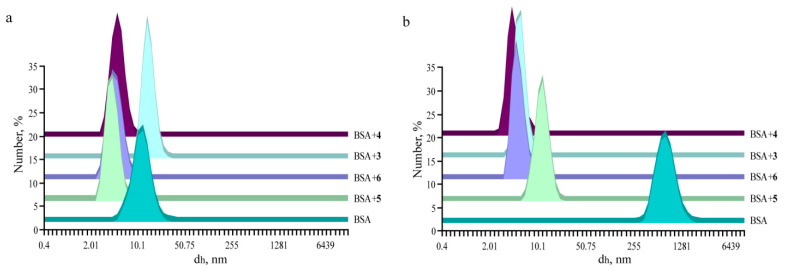
The number-averaged particle size distribution for freshly prepared BSA and BSA–macrocycle solutions (**a**) and after 2 weeks of standing (**b**), PB, pH 7, 25 °C.

To study macrocycle–BSA interactions, the particle sizes in BSA solutions in the absence and presence of macrocycles were measured by the DLS method at two concentrations of macrocycles, 1 × 10^−4^ and 1 × 10^−3^ M (Figure 4). It should be noted that in individual macrocycle solutions, a high polydispersity index (PDI) of 0.392–0.781 was observed, and the average particle sizes (hydrodynamic diameter averaged by number) were 2.3–4.2 nm (Table S3). In the freshly prepared solution of BSA in the absence of macrocycles, the particle size was 10.1 ± 4.8 nm (PDI 0.323 ± 0.131). Serum albumins are globular proteins with a molecular size of 4 × 4 × 14 nm^3^ [15], thus, the presence of BSA monomers or dimers in individual solutions can be assumed. After 2 weeks of the solution standing at room temperature, the average size of BSA particles was 712.4 ± 6.63 nm (PDI 0.219 ± 0.006), which meant the self-aggregation of protein molecules in the solution.

In freshly prepared macrocycle–BSA solutions in the presence of 1 × 10^−4^ M macrocycles, the sizes of the particles were 4.9–7.5 nm (PDI 0.334–0.484), and after 2 weeks, the particle sizes in solutions with zwitterionic macrocycles **5** and **6** remained unchanged (7.5 nm), but in the solutions with cationic esters **3** and **4**, a precipitation was observed. In the freshly prepared solutions with a concentration of macrocycles of 1 × 10^−3^ M, the existence of particles with sizes of 4.2–13.5 nm (PDI 0.279–0.408) was observed, and after 2 weeks of standing, they were 4.2–11.7 nm (PDI 0.543–0.786) (Table S3, Figure 4).

Thus, during BSA solution standing, the self-aggregation and enlargement of protein particles were observed; in the solutions with a 10-fold excess of macrocycles, the precipitation in the presence of cationic macrocycles **3** and **4** could be explained by compensation of the negative charge on the BSA surface. In the presence of a 10-fold excess of zwitterionic macrocycles **5** and **6** and a 100-fold excess of macrocycles **3**–**6**, no particle enlargement or precipitation was caused, which could suggest the prevention of the self-aggregation of BSA molecules due to the formation of BSA–macrocycle complexes.

Thus, macrocycles can bind BSA in the solution due to the formation of H-bonds, van der Waals forces, and electrostatic (in the case of ether macrocycles **3** and **4**) and hydrophobic interactions. The excess of macrocycles in the solution prevented the self-aggregation of BSA molecules. The lower *K*_as_ values of BSA–zwitterionic macrocycle complexes in conditions close to physiological temperatures (lg*K*_as_ 3.62–3.58 at 308 K) could probably mean their inertness toward the serum albumins in the bloodstream.

## 3. Materials and Methods

The syntheses of compounds **1** and **2** and their ^1^H NMR, IR and mass spectra are presented in Supplementary Materials. The initial calix[4]resorinarenes with pentyl and undecyl substituents on the lower rim were synthesized as described in ref. [54]. The syntheses of the compounds **3**–**6** were first reported in this study. BSA (≥96.0%) and pyrene (99%) were obtained from Sigma-Aldrich (St. Louis, MO, USA). The stock solution of 10.0 × 10^−6^ mol L^−1^ BSA was prepared in 0.1 mol L^−1^ phosphate buffer solution (PB) (pH = 7.0). The stock solution of pyrene was prepared in ethanol at a concentration of 9.71 × 10^−4^ mol L^−1^.

^1^H and ^13^C NMR spectra were performed on an MSL-400 spectrometer (400 MHz for ^1^H NMR, 100 MHz for ^13^C NMR). The chemical shifts were reported relative to the residual solvent peaks as internal standards. IR spectra were recorded with a Vector 22 Spectrometer in KBr pellets. Electrospray ionization mass spectra (ESI) were obtained on an AmazonX mass spectrometer (Bruker Daltonik GmbH, Bremen, Germany).

### 3.1. General Procedure for the Synthesis of Compounds ***3*** and ***4***

Compound **1** (8.00 g, 4.47 mmol) or **2** (8.04 g, 3.77 mmol) was dissolved in 200 mL of dry acetonitrile and 4.00 mL (36.0 mmol) or 3.35 mL (30.0 mmol), respectively, of ethyl bromoacetate added under stirring. The reaction mixture was heated at 60 °C for 44 h. The reaction mixture was filtrated, and the filtrate was dried under reduced pressure.

2,8,14,20-Tetrapentylpentacyclo[19.3.1.1^3,7^.1^9,13^.1^15,19^]-octacosa-1(25),3,5,7(28),9,11,13(27),15,17,19(26),21,23-dodecaen-4,6,10,12,16,18,22,24-octakis{N-[3-(dimethyl{ethoxycarbonylmethyl}ammonio)ethyl]aminocarbonylmethoxy} octabromide **3**. Yield 13.01 g (93.3%); m.p. 148–150 °C; ^1^H NMR (400 MHz, D_2_O): δ = 0.74 (t, ^3^*J*_H,H_ = 8 Hz, 12H; CH_2_(CH_2_)_3_CH_3_), 1.20 (m, 48H; CH_2_(CH_2_)_3_CH_3_, OCH_2_CH_3_), 1.75 (br s, 8H, CH_2_(CH_2_)_3_CH_3_), 3.33 (s, 48H; N^+^(CH_3_)_2_), 3.77 (br s, 16H; C(O)HNCH_2_CH_2_N^+^), 3.86 (br s, 16H; C(O)HNCH_2_CH_2_N^+^), 4.21 (br s, 16H; OCH_2_CH_3_), 4.42 (br s, 16H; OCH_2_C(O)), 4.44 (br s, 16H; N^+^CH_2_C(O)O), 4.70 (s, 4H; CH), 6.61 (s, 8H; ArH). ^1^H NMR (500 MHz, CD_3_OD): δ = 0.88 (t, ^3^*J*_H,H_ = 6.9 Hz, 12H; CH_2_(CH_2_)_3_CH_3_), 1.32 (t, 24H; OCH_2_CH_3_), 1.34 (s, 24H; CH_2_(CH_2_)_3_CH_3_), 1.86 (s, 8H, CH_2_(CH_2_)_3_CH_3_), 3.46 (s, 48H; N^+^(CH_3_)_2_), 3.89 (m, 32H; C(O)HNCH_2_CH_2_N^+^), 4.32 (q, ^3^*J*_H,H_ = 7.2 Hz, 16H; OCH_2_CH_3_), 4.44 (br s, 16H; OCH_2_C(O)), 4.59 (s, 16H; N^+^CH_2_C(O)O), 4.70 (t, ^3^*J*_H,H_ = 7.2 Hz, 4H; CH), 6.68 (s, 4H; ArH), 6.96 (s, 4H; ArH). ^13^C{^1^H} NMR (100 MHz, D_2_O) δ = 170.5, 164.6, 153.5, 128.4, 125.6, 68.4, 63.0, 62.0, 61.3, 52.4, 35.0, 34.4, 33.0, 31.8, 26.6, 21.9, 13.7. ^13^C{^1^H} NMR (126 MHz, CD_3_OD) δ = 171.8, 166.1, 155.8, 130.9, 127.8, 104.5, 70.8, 64.6, 64.1, 63.1, 53.2, 37.2, 36.0, 34.7, 33.5, 29.0, 23.9, 14.7, 14.5. IR: $\tilde{\nu }$ = 3411 (NH), 2930 (CH), 2859 (CH), 1748 (C=O), 1671 (Amid I), 1534 (Amid II), 1500 (C=C_Ar_), 1204 (C-O). MS (ESI): *m/z* calcd for C_128_H_216_Br_8_N_16_O_32_: 963.5 [M − 3Br^−^]^3+^; found 963.4; 702.7 [M − 4Br^−^]^4+^; found 702.4; 546.2 [M − 5Br^−^]^5+^; found 546.1; 441.8 [M − 6Br^−^]^6+^; found 441.7; elemental analysis calcd (%) for C_128_H_216_Br_8_N_16_O_32_6H_2_O: C 47.47, H 7.10, Br 19.47, N 6.92; found: C 47.46, H 7.16, Br 19.39, N 6.80.

2,8,14,20-Tetraundecylpentacyclo[19.3.1.1^3,7^.1^9,13^.1^15,19^]-octacosa-1(25),3,5,7(28),9,11,13(27),15,17,19(26),21,23-dodecaen-4,6,10,12,16,18,22,24-octakis{N-[3-(dimethyl{ethoxycarbonylmethyl}ammonio)ethyl]aminocarbonylmethoxy} octabromide **4**. Yield 11.84 g (95%); m.p. 155–156 °C; ^1^H NMR (400 MHz, D_2_O): δ = 0.77 (br s, 12H; CH_2_(CH_2_)_9_CH_3_), 1.20 (m, 96H; CH_2_(CH_2_)_9_CH_3,_ OCH_2_CH_3_), 1.99 (br s, 8H, CH_2_(CH_2_)_9_CH_3_), 3.39 (s, 48H; N^+^(CH_3_)_2_), 3.87 (br s, 16H; C(O)HNCH_2_CH_2_N^+^), 3.40 (br s, 16H; C(O)HNCH_2_CH_2_N^+^), 4.26 (br s, 16H; OCH_2_CH_3_), 4.49 (br s, 16H; N^+^CH_2_C(O)O), 4.50 (br s, 16H; OCH_2_C(O)), 4.78 (s, 4H; CH), 6.61 (s, 8H; ArH). ^1^H NMR (500 MHz, CD_3_OD): δ = 0.89 (t, ^3^*J*_H,H_ = 6.9 Hz, 12H; CH_2_(CH_2_)_9_CH_3_), 1.27 (s, 72H; CH_2_(CH_2_)_9_CH_3_), 1.32 (t, ^3^*J*_H,H_ = 7.2 Hz, 24H; OCH_2_CH_3_), 1.86 (s, 8H, CH_2_(CH_2_)_9_CH_3_), 3.47 (s, 48H; N^+^(CH_3_)_2_), 3.88 (m, 16H; C(O)HNCH_2_CH_2_N^+^), 3.91 (m, 16H; C(O)HNCH_2_CH_2_N^+^), 4.32 (q, ^3^*J*_H,H_ = 7.2 Hz 16H; OCH_2_CH_3_), 4.44 (br s, 16H; OCH_2_C(O)), 4.61 (s, 16H; N^+^CH_2_C(O)O), 4.72 (m, 4H; CH), 6.68 (s, 4H; ArH), 6.88 (s, 4H; ArH). ^13^C{^1^H} NMR (126 MHz, CD_3_OD) δ = 171.7, 166.1, 155.8, 130.1, 127.8, 104.4, 70.8, 64.6, 64.0, 63.1, 53.2, 37.2, 36.1, 34.7, 33.5, 23.9, 14.7, 14.5. IR: $\tilde{\nu }$ = 3411 (NH), 2924 (CH), 2853 (CH), 1747 (C=O), 1672 (Amid I), 1533 (Amid II), 1500 (C=C_Ar_), 1203 (C-O). MS (ESI): *m/z* calcd for C_152_H_264_Br_8_N_16_O_32_: 1075.8 [M − 3Br^−^]^3+^; found 1075.9; 786.9 [M − 4Br^−^]^4+^; found 787.3; 613.5 [M − 5Br^−^]^5+^; found 613.5; 415.4 [M − 7Br^−^]^7+^; found 415.6; elemental analysis calcd (%) for C_152_H_264_Br_8_N_16_O_32_9H_2_O: C 50.30, H 7.83, Br 17.61, N 6.18; found: C 50.34, H 7.80, Br 17.69, N 6.11.

### 3.2. General Procedure for the Synthesis of Compounds ***5*** and ***6***

Compound **3** (4.00 g, 1.30 mmol) or **4** (5.00 g, 1.45 mmol) was dissolved in 100 mL of ethanol and 3 mL of an aqueous solution of NaOH (1.28 g, 32.0 mmol or 1.45 g, 36.0 mmol, respectively) added under stirring. The reaction mixture was heated at 60 °C for 23 h. After cooling to rt, the precipitate was collected, washed by ethanol, and dried. The precipitate was dissolved in distilled water (4 mL), and the pH of the solution was adjusted to pH 7.5 by the addition of 0.1 M HCl. The resulting solutions were dialyzed under distilled water (molecular weight cutoff, 1000 Da), followed by evaporation of the aqueous solution and drying of the product under reduced pressure.

2,8,14,20-Tetrapentyl-4,6,10,12,16,18,22,24-octakis{N-[2,8,14,20-Tetrapentylpentacyclo[19.3.1.1^3,7^.1^9,13^.1^15,19^]-octacosa-1(25),3,5,7(28),9,11,13(27),15,17,19(26),21,23-dodecaen-4,6,10,12,16,18,22,24-octakis{N-[3-(dimethyl{acetoxido}ammonio)ethyl]aminocarbonylmethoxy} **5**. Yield 2.70 g (91.8%); m.p. 198–200 °C; ^1^H NMR (400 MHz, D_2_O): δ = 0.62 (br s, CH_2_(CH_2_)_3_CH_3_), 1.12 (m, 24H; CH_2_(CH_2_)_3_CH_3_), 1.70 (br s, 8H, CH_2_(CH_2_)_3_CH_3_), 3.22 (s, 48H; N^+^(CH_3_)_2_), 3.59 (br s, 16H; C(O)HNCH_2_CH_2_N^+^), 3.72 (br s, 16H; C(O)HNCH_2_CH_2_N^+^), 3.87 (br s, 16H; CH_2_C(O)O^−^), 4.27 (br s, 16H; OCH_2_C(O)), 4.60 (s, 4H; CH), 6.42 (s, 4H; ArH), 6.53 (s, 4H; ArH). ^1^H NMR (500 MHz, CD_3_OD): δ = 0.89 (t, ^3^*J*_H,H_ = 6.9 Hz, CH_2_(CH_2_)_3_CH_3_), 1.34 (s, 24H; CH_2_(CH_2_)_3_CH_3_), 1.87 (s, 8H, CH_2_(CH_2_)_3_CH_3_), 3.34 (s, 48H; N^+^(CH_3_)_2_), 3.80 (s, 16H; C(O)HNCH_2_CH_2_N^+^), 3.86 (s, 16H; C(O)HNCH_2_CH_2_N^+^), 3.95 (s, 16H; CH_2_C(O)O^−^), 4.30 (br s, 16H; OCH_2_C(O)), 4.65 (s, 4H; CH), 6.52 (s, 4H; ArH), 6.71 (s, 4H; ArH). ^13^C{^1^H} NMR (100 MHz, D_2_O) δ = 170.3, 168.0, 128.4, 125.6, 125.5, 100.2, 63.8, 68.2, 60.9, 51.3, 34.6, 32.7, 31.7, 26.5, 22.0, 13.4. ^13^C{^1^H} NMR (126 MHz, CD_3_OD) δ = 171.7, 168.8, 155.9, 129.3, 127.8, 104.4, 70.7, 66.0, 63.1, 52.7, 37.1, 35.9, 34.7, 33.4, 29.0, 14.7. IR: $\tilde{\nu }$ = 3400 (NH), 2929 (CH), 2858 (CH), 1631 (Amid I), 1541 (Amid II), 1500 (C=C_Ar_). MS (ESI): *m/z* calcd for C_112_H_176_N_16_O_32_: 1152.3 [M + 2Na]^2+^; found 1152.2; 775.9 [M + 3Na]^3+^; found 775.9; elemental analysis calcd (%) for C_112_H_176_N_16_O_32_14H_2_O: C 53.57, H 8.19, N 8.93; found: C 53.61, H 8.15, N 8.80.

2,8,14,20-Tetraundecylpentacyclo[19.3.1.1^3,7^.1^9,13^.1^15,19^]-octacosa-1(25),3,5,7(28),9,11,13(27),15,17,19(26),21,23-dodecaen-4,6,10,12,16,18,22,24-octakis{N-[3-(dimethyl{acetoxido}ammonio)ethyl]aminocarbonylmethoxy} **6**. Yield 3.50 g (93.0%); m.p. 211–220 °C; ^1^H NMR (400 MHz, D_2_O): δ = 0.77 (br s, 12H; CH_2_(CH_2_)_9_CH_3_), 1.30 (m, 72H; CH_2_(CH_2_)_9_CH_3_), 1.99 (br s, 8H, CH_2_(CH_2_)_9_CH_3_), 3.39 (s, 48H; N^+^(CH_3_)_2_), 3.87 (br s, 16H; C(O)HNCH_2_CH_2_N^+^), 3.88 (br s, 16H; C(O)HNCH_2_CH_2_N^+^), 4.49 (br s, 16H; CH_2_C(O)O^−^), 4.50 (br s, 16H; OCH_2_C(O)), 4.78 (s, 4H; CH), 6.45 (s, 4H; ArH), 7.21 (s, 4H; ArH). ^1^H NMR (500 MHz, CD_3_OD): δ = 0.89 (t, ^3^*J*_H,H_ = 7.2 Hz, CH_2_(CH_2_)_3_*CH*_3_), 1.27 (s, 72H; CH_2_(CH_2_)_9_CH_3_), 1.86 (s, 8H, CH_2_(CH_2_)_3_CH_3_), 3.34 (s, 48H; N^+^(CH_3_)_2_), 3.78 (s, 16H; C(O)HNCH_2_CH_2_N^+^), 3.80 (s, 16H; C(O)HNCH_2_CH_2_N^+^), 3.92 (s, 16H; CH_2_C(O)O^−^), 4.30 (br s, 16H; OCH_2_C(O)), 4.62 (s, 4H; CH), 6.52 (s, 4H; ArH), 7.13 (s, 4H; ArH). ^13^C{^1^H} NMR (126 MHz, CD_3_OD) δ = 176.8, 172.0, 168.9, 156.0, 130.3, 127.4, 107.8, 70.8, 65.6, 63.2, 52.4, 36.6, 34.6, 33.3, 31.0, 23.9, 14.6. IR: $\tilde{\nu }$ = 3409 (NH), 2924 (CH), 2853 (CH), 1626 (Amid I), 1542 (Amid II), 1499 (C=C_Ar_). MS (ESI): *m/z* calcd for C_136_H_224_N_16_O_32_: 888.1 [M + 3Na]^3+^; found 888.0; elemental analysis calcd (%) for C_136_H_224_N_16_O_32_14H_2_O: C 57.36, H 8.92, N 7.87; found: C 57.41, H 8.89, N 7.86.

### 3.3. Hemolytic Activity Assay

Fresh hRBC with heparin was rinsed 3 times with 0.15 M NaCl by centrifugation at 800 rpm for 10 min and re-suspended in 0.15 M NaCl. Each of the investigated solutions in 0.15 M NaCl was then added to 0.5 mL of a solution of the stock hRBC in 0.15 M NaCl to reach a final volume of 5 mL (final erythrocyte concentration 10% *v/v*). The resulting suspension was incubated under agitation for 1 h at 37 °C. The samples were then centrifuged at 2000 rpm for 10 min. The release of hemoglobin was monitored by measuring the absorbance of the supernatant at 540 nm by means of a digital photoelectric colorimeter AP-101 (Apel, Tokyo, Japan). Controls for zero hemolysis (blank) and 100% hemolysis consisted of hRBC, which was suspended in 0.15 M NaCl, and bidistilled water, respectively.

### 3.4. Cytotoxic Effect Assay

Chang liver cell and M-HeLa clone 11 cell viability was evaluated by means of multifunctional system Cytell Cell Imaging (GE Healthcare Life Sciences, Sweden) using the Cell Viability BioApp application, which makes it possible to precisely count the number of cells and estimate their viability from the fluorescence intensity [41]. Cells were cultured in a standard Eagle’s nutrient medium manufactured at the Chumakov Institute of Poliomyelitis and Virus Encephalitis (PanEco Company, Moscow, Russia) and supplemented with 10% fetal calf serum and 1% nonessential amino acids. The cells were dispersed on a 24-well «Eppendorf» plate at a concentration of 200 × 10^3^ cells/mL, 500 μL of medium per well and cultured in a CO_2_ incubator at 37 °C. After 24 h seeding the cells into wells, the examined compounds were added at a preset dilution, 500 μL to each well. The twofold dilutions of the compounds were prepared immediately in nutrient media. The resulting suspensions were incubated for 24 h at 37 °C. The experiments were performed in triplicate. Intact cells cultured in parallel with experimental cells served as a reference. The fraction of the grown-up cells was expressed in % vs. reference cells. The degree of cell growth inhibition under the influence of the testing agent was calculated by the equation: N(%) = (1 − Exp/Control)·100%, where Exp is the quantity of uninhibited cells in the study sample, and Control is the quantity of uninhibited cells in the control sample. Then, IC_50_ (the concentration that caused 50% cell growth inhibition) was determined from the curve of cell cultural growth versus the examined compound concentration.

### 3.5. Antiaggregant and Anticoagulant Activities Assay

The experiments were performed in compliance with the requirements of the Good Laboratory Practice Rules of the Eurasian Economic Union in the field of medicine circulation. Antiaggregant and anticoagulant activities were assessed under in vitro conditions in isolated blood fractions from 79 healthy male volunteers 18–24 years old. The research was approved by the Ethics Committee of BSMU, Ministry of Health of Russia (No. 1 dated 20 February 2019). Informed consent was obtained from all participants before collecting blood. The influence of the compounds on platelet aggregation was studied using the Born method [42] on an AT-02 aggregometer (SPC Medtekh, Kazan, Russia). The antiaggregant activities of the tested compounds and the reference drug were assessed at a final concentration of 1 mM with incubation for 5 min. Adenosine diphosphate (ADP) at a concentration of 20 µg/mL (Tekhnologiya-Standart, Barnaul, Russia) was used as aggregation inducer. The influence of the compounds on the maximum amplitude (MA), aggregation rate, and time to reach MA during ADP-induced platelet aggregation was studied. The reference drug was acetylsalicylic acid (powder substance; Shandong Xinhua Pharmaceutical Co. Ltd., Zibo, China).

Anticoagulant activity was determined by clotting tests [55] in a Solar CGL2110 turbidimetric hemocoagulometer (Zao Solar, Minsk, Belarus). The final concentration of the tested compounds and the reference drug was 0.5 mg/mL. The activated partial thromboplastin time (APTT), prothrombin time (PT), and fibrinogen concentration according to Clauss were studied. The reference drug was heparin sodium (5000 IU/mL solution for injection, 1-mL ampuls, OAO Sintez, Moscow, Russia).

Statistical analysis used the Statistica 10.0 software (StatSoft Inc., Tulsa, OK, USA). A check for normal distributions used the Shapiro–Wilk criterion. Variational series were described by calculating the median, 25 and 75 percentiles, and minimum and maximum values. One-factor dispersion analysis (if a dataset obeyed normal distribution laws, and the dispersions of all sets were equal; F-criterion) or the Kruskal–Wallis test (if a dataset did not obey normal distribution laws; A-criterion) was performed. The critical significance level P for statistical criteria was set to 0.05.

### 3.6. Determination of Cac Values of Compounds ***3***–***6*** by Fluorimetry Method with Pyrene Probe

The steady-state emission spectra were recorded on a fluorescence spectrophotometer Hitachi F-7100. A quartz cell of 1 cm path length was used for all fluorescence measurements, and spectra were recorded at 25 °C. In the solutions, the concentration of pyrene was maintained at 2 × 10^−6^ mM, whereas the concentrations of calix[4]resorcinarenes varied from 0 to 1 × 10^−3^ M. Fluorescence spectra were obtained using 2.5/2.5 nm (excitation/emission) slit widths. The excitation wavelength was set at 333 nm, and the emission range was from 345 to 500 nm. The ratio of first (372 nm) and third (381 nm) emission bands I/III for every spectrum was estimated, and the cac values were graphically determined from the sigmoidal plots of I/III ratio versus the logarithm of calix[4]resorcinarene concentration according to ref [56].

### 3.7. Investigation of Compounds ***3***–***6***—BSA Interaction

A solution of BSA with the concentration of 1 × 10^−5^ M in phosphate buffer solution (pH 7.0) was used. The steady-state emission spectra were recorded on a fluorescence spectrophotometer Hitachi F-7100. A quartz cell of 1 cm path length was used for all fluorescence measurements, and spectra were recorded at 20, 30 and 35 °C. In the solutions, the concentration of BSA was maintained at 1 × 10^−5^ M, whereas the concentrations of calix[4]resorcinarenes varied from 0 to 1 × 10^−3^ M. Fluorescence spectra were obtained using 2.5/2.5 nm (excitation/emission) slit widths. The excitation wavelength was set at 279 nm, and the emission range was from 290 to 500 nm. Absorbance measurements were performed in a Lambda 35 UV-vis spectrometer (Perkin Elmer Instruments, Shelton, CT, USA) in quartz cells of 0.5 cm path length. Synchronous fluorescence spectra of BSA in the presence of calix[4]resorcinarenes were registered using two fixed differences in the emission and excitation wavelengths Δλ (15 and 60 nm). CD spectra of BSA in the absence and presence of calix[4]resorcinarenes were recorded with a J-1500 Circular Dichroism Spectrophotometer. A cuvette with an optical path length of 1 mm was used for the record of CD spectra. Far-CD spectra were recorded from 250 to 205 nm, and the scanning speed was 200 nm/min. Spectra were registered at 25 °C using a phosphate buffer (pH 7.0) as a reference. DLS measurements were carried out on a Zetasizer Nano-ZS (Malvern Instruments, UK) using Dispersion Technology Software 5.00. Each solution was tested in at least three identical solutions. The error of hydrodynamic particle size determination was 2%.

## 4. Conclusions

Thus, we synthesized and studied four new amphiphilic calix[4]resorcinarenes with carboxybetaine and carboxybetaine ester groups on the upper rim. It was shown that due to the aliphatic groups on the lower rim, they can form self-associates in solution, with the lowest cac value of 2.7 × 10^−5^ M for zwitterionic tetraundecylcalix[4]resorcinarene **6**. The cytotoxic effects of macrocycles on normal and tumor cells were studied; herewith, carboxybetaine calix[4]resorcinarenes were less toxic than esters, which, moreover, were more cytotoxic on normal cells. The influence of macrocycles on blood components, namely erythrocytes, platelets, and the plasma coagulation system, was studied. In the studied concentration range (0–5 × 10^−3^ M), almost complete absence of destruction of blood erythrocytes was shown. Additionally, it was shown that macrocycles exhibit weak anticoagulant activity (especially in the case of zwitterionic macrocycle **5**) and platelet anti-aggregation activity at the level of acetylsalicylic acid. A complex of methods was used to study the macrocycle interactions with a model blood plasma protein, BSA. It was shown that the binding of BSA by macrocycles includes various intermolecular forces (H-bonds, van der Waals forces, electrostatic interaction, and hydrophobic effect) and leads to a change in the BSA conformation but prevents its self-aggregation in the presence of an excess of macrocycles in solution. At the same time, under conditions close to physiological, the complex formation of BSA by zwitterionic macrocycles was weak (lg*K*_as_ 3.58, 3.62 for tetraundecylcalix[4]resorcinarene **6** and tetrapentylcalix[4]resorcinarene **5**, respectively). An analysis of the obtained data showed that carboxybetaine calix[4]resorcinarenes and, to a lesser extent, carboxybetaine esters, have low toxicity, especially to blood components, and can be used to create therapeutic nanosystems.

## Figures and Tables

**Table 1:** The cytotoxic effect (IC_50_, µM) on normal and cancer cells and hemolytic activity (HC_50_, µM) of compounds **3**–**6**.

Macrocycle	IC_50_ (µM)		HC_50_ (µM)
	Chang Liver	M-HeLa	
**3**	688	1244	>5000
**4**	1219	2166	>5000
**5**	6000	4600	>5000
**6**	3200	2400	>5000

**Table 2:** Effect of compounds **3**–**6** on hemostasis coagulation stage and platelet aggregatio (median ^1^ 0.25–0.75).

Macrocycle	APTT, % of Control	Maximum Amplitude, % of Control	Aggregation Rate, % of Control	Time to Reach of Maximum Amplitude, % of Control
**3**	+4.9 (3.7–5.6)	−15.7 (13.7–18.9) *	−3.8 (2.7–5.9) #	+6.3 (4.6–9.7) *
**4**	+6.2 (5.7–7.8)	−16.9 (15.3–20.7) *	−9.4 (8.2–11.9) *	+9.4 (7.3–12.7) *
**5**	+8.4 (7.5–10.2)	−13.4 (11.9–14.5) *	−2.3 (1.7–4.2) #	+6.7 (4.3–8.5) *
**6**	+3.1 (2.7–6.5)	−9.8 (7.6–11.9) *	−14.5 (10.4–17.3) *	+13.7 (8.9–15.2) *
Heparin	+20.3 (19.7–21.4) **	-	-	-
Acetylsalicylic acid	-	−13.7 (10.8–16.4) *	−10.5 (7.6–12.3) *	+10.5 (8.7–13.4) *

^1^ average value. * *p* ≤ 0.05, ** *p* ≤ 0.001 compared to control; # *p* ≤ 0.05 compared to acetylsalicylic acid; n = 6.

**Table 3:** Calculated parameters of BSA–macrocycle associations in PB (pH 7.4).

System	T, K	*K*_SV_ 10^−3^_,_ L mol^−1^	*k*_q_ 10^−12^, L mol^−1^ s^−1^	lg*K*_as_	n	ΔH°, kJ mol^−1^	ΔS°, J mol^−1^	ΔG°, kJ mol^−1^
3 + BSA	293	15.1	1.51	6.42	1.6	−182.841	−500.8	−36.1
	303	13.4	1.34	5.39	1.3			
	308	12.8	1.28	4.83	1.0			
4 + BSA	293	11.8	1.18	4.83	1.2	−107.3	−274.3	−27.0
	303	11.4	1.14	4.08	1.0			−24.2
	308	8.7	0.87	3.93	1.0			−22.8
5 + BSA	293	5.9	0.59	4.19	1.1	−59.0	−121.2	−23.5
	303	5.6	0.56	3.83	1.0			−22.3
	308	5.5	0.55	3.62	1.0			−21.7
6 + BSA	293	6.1	0.61	3.90	1.1	−37.0	−51.5	−21.9
	303	4.2	0.42	3.68	1.1			−21.4
	308	1.7	0.17	3.58	1.1			−21.1

## Data Availability

The data presented in this study are available in Supplementary Materials.

## Supplementary Materials

The following supporting information can be downloaded at: https://www.mdpi.com/article/10.3390/ijms232315298/s1, Figures S1–S12: ^1^H NMR, IR and mass-spectra of compounds **9**, **10**, **1** and **2**; Figures S13–S38: ^1^H NMR, ^13^C NMR, COSY, HSQC, HMBC spectra of compounds **3**–**6**; Figures S39–S46: IR and ESI spectra of compounds **3**–**6**. Figure S47. The pyrene I/III values dependence on the logarithmic concentration of compounds **3**–**6**; Figures S48–S51: The fluorescence and absorbance spectra of BSA-compound **3**–**6** solutions; Table S1: Cytotoxicity of **3**–**6**; Table S2: Hemolytic activity of **3**–**6**; Table S3: DLS data for BSA-macrocycle **3**–**6** solutions. Reference [54] is cited in the supplementary materials.

## References

[1] Gu A., Wheate N.J. (2021). Macrocycles as drug-enhancing excipients in pharmaceutical formulations. J. Incl. Phenom. Macrocycl. Chem..

[2] Fahmy S.A., Brüßler J., Alawak M., El-Sayed M.M.H., Bakowsky U., Shoeib T. (2019). Chemotherapy based on supramolecular chemistry: A promising strategy in cancer therapy. Pharmaceutics.

[3] Yu G., Chen X. (2019). Host–guest chemistry in supramolecular theranostics. Theranostics.

[4] Gao L., Wang H., Zheng B., Huang F. (2021). Combating antibiotic resistance: Current strategies for the discovery of novel antibacterial materials based on macrocycle supramolecular chemistry. Giant.

[5] Yin H., Zhang X., Wei J., Lu S., Bardelang D., Wang R. (2021). Recent advances in supramolecular antidotes. Theranostics.

[6] Sowa A., Voskuhl J. (2020). Host-guest complexes—Boosting the performance of photosensitizers. Int. J. Pharm..

[7] Geng W.-C., Huang Q., Xu Z., Wang R., Guo D.-S. (2019). Gene delivery based on macrocyclic amphiphiles. Theranostics.

[8] Tauran Y., Coleman A.W., Perret F., Kim B. (2015). Cellular and in vivo biological activities of the calix[n]arenes. Curr. Org. Chem..

[9] Fan X., Guo X. (2021). Development of calixarene-based drug nanocarriers. J. Mol. Liq..

[10] Razuvayeva Y., Kashapov R., Zakharova L. (2020). Calixarene-based pure and mixed assemblies for biomedical applications. Supramol. Chem..

[11] Sobczynski D.J., Fish M.B., Fromen C.A., Carasco-Teja M., Coleman R.M., Eniola-Adefeso O. (2015). Drug carrier interaction with blood: A critical aspect for high-efficient vascular-targeted drug delivery systems. Ther. Deliv..

[12] De la Harpe K.M., Kondiah P.P.D., Choonara Y.E., Marimuthu T., du Toit L.C., Pillay V. (2019). The hemocompatibility of nanoparticles: A review of cell–nanoparticle interactions and hemostasis. Cells.

[13] Silva E.D., Rousseau C.F., Zanella-Cléon I., Becchi M., Coleman A.W. (2006). Mass spectrometric determination of association constants of bovine serum albumin (BSA) with *para*-sulphonato-calix[*n*]arene derivatives. J. Incl. Phenom. Macrocycl. Chem..

[14] Solanki R., Rostamabadi H., Patel S., Jafari S.M. (2021). Anticancer nano-delivery systems based on bovine serum albumin nanoparticles: A critical review. Int. J. Biol. Macromol..

[15] Memmi L., Lazar A., Brioude A., Ball V., Coleman A.W. (2001). Protein–calixarene interactions: Complexation of bovine serum albumin by sulfonatocalix[n]arenes. Chem. Commun..

[16] Ukhatskaya E.V., Kurkov S.V., Rodik R.V., Kalchenko V.I., Matthews S.E., Jansook P., Loftsson T. (2014). Surface activity and self-aggregation ability of three cationic quaternized aminocalix[4]arenes. J. Incl. Phenom. Macrocycl. Chem..

[17] Morozova J.E., Syakaev V.V., Kazakova E.K., Shalaeva Y.V., Nizameev I.R., Kadirov M.K., Voloshina A.D., Zobov V.V., Konovalov A.I. (2016). Amphiphilic calixresorcinarene associates as effective solubilizing agents for hydrophobic organic acids: Construction of nano-aggregates. Soft Matter..

[18] Morozova J.E., Myaldzina C.R., Voloshina A.D., Lyubina A.P., Amerhanova S.K., Syakaev V.V., Kazakova E.K., Ziganshina A.Y., Antipin I.S. (2022). Calixresorcine cavitands bearing lipophilic cationic fragments in the construction of mitochondrial-targeting supramolecular nanoparticles. Colloids Surf. A.

[19] Kashapov R.R., Razuvayeva Y.S., Ziganshina A.Y., Mukhitova R.K., Sapunova A.S., Voloshina A.D., Nizameev I.R., Kadirov M.K., Zakharova L.Y. (2020). Design of N-methyl-D-glucamine-based resorcin[4]arene nanoparticles for enhanced apoptosis effects. Mol. Pharm..

[20] Shumatbaeva A.M., Morozova J.E., Syakaev V.V., Zakharychev D.V., Sapunova A.S., Voloshina A.D., Bekmuratova F.A., Babaev V.M., Antipin I.S. (2021). A novel salt-responsive hydrogel on the base of calixresorcinarene–mPEG amide conjugate. Colloids Surf. A.

[21] Shumatbaeva A.M., Morozova J.E., Syakaev V.V., Shalaeva Y.V., Sapunova A.S., Voloshina A.D., Gubaidullin A.T., Bazanova O.B., Babaev V.M., Nizameev I.R. (2020). The pH-responsive calix[4]resorcinarene-mPEG conjugates bearing acylhydrazone bonds: Synthesis and study of the potential as supramolecular drug delivery systems. Colloids Surf. A.

[22] Shumatbaeva A.M., Morozova J.E., Shalaeva Y.V., Gubaidullin A.T., Saifina A.F., Syakaev V.V., Bazanova O.B., Sapunova A.S., Voloshina A.D., Nizameev I.R. (2019). The novel calix[4]resorcinarene-PEG conjugate: Synthesis, self-association and encapsulation properties. Colloids Surf. A.

[23] Ermakova A.M., Morozova J.E., Shalaeva Y.V., Syakaev V.V., Gubaidullin A.T., Voloshina A.D., Zobov V.V., Nizameev I.R., Bazanova O.B., Antipin I.S. (2018). Nanoconjugates of a calixresorcinarene derivative with methoxy poly(ethylene glycol) fragments for drug encapsulation. Beilstein J. Nanotechnol..

[24] Syakaev V.V., Morozova J.E., Bogdanov A.V., Shalaeva Y.V., Ermakova A.M., Voloshina A.D., Zobov V.V., Nizameev I.R., Kadirov M.K., Mironov V.F. (2018). Solubilization of azo-dye-modified isatin derivative by amphiphilic carboxyresorcinarenes: The effect of macrocycle structure on the supramolecular association. Colloids Surf. A.

[25] Silva E.D., Ficheux D., Coleman A.W. (2005). Anti-thrombotic activity of water-soluble calix[n]arenes. J. Incl. Phenom. Macrocycl. Chem..

[26] Rekkab S., Lahouel M., Hadda T.B., Félix C., Kabouche Z. (2013). Design, synthesis and anticoagulant activity of new flexible calix[8]arene sulfonic acids. Comptes Rendus Chim..

[27] Jebors S., Fache F., Balme S., Devoge F., Monachino M., Cecillon S., Coleman A.W. (2008). Designer amphiphiles based on para-acyl-calix[8]arenes. Org. Biomol. Chem..

[28] Lugovskoy E.V., Gritsenko P.G., Koshel T.A., Koliesnik I.O., Cherenok S.O., Kalchenko O.I., Kalchenko V.I., Komisarenko S.V. (2011). Calix[4]arene methylenebisphosphonic acids as inhibitors of fibrin polymerization. FEBS J..

[29] Chernyshenko V.O., Korolova D.S., Dosenko V.E., Pashevin D.O., Kalchenko V.I., Pirogova L.V., Chernyshenko T.M., Lugovska O.E., Kravchenko N.A., Makogonenko Y.M. (2015). Calix[4]arene C-145 effects on plasma haemostasis. Pharm. Anal. Acta.

[30] Yakimova L., Kunafina A., Mostovaya O., Padnya P., Mukhametzyanov T., Voloshina A., Petrov K., Boldyrev A., Stoikov I. (2022). Albumin/thiacalix[4]arene nanoparticles as potential therapeutic systems: Role of the macrocycle for stabilization of monomeric protein and self-assembly with ciprofloxacin. Int. J. Mol. Sci..

[31] Shalaeva Y.V., Morozova J.E., Shumatbaeva A.M., Nizameev I.R., Kadirov M.K., Antipin I.S. (2019). Binding of L-tryptophan and bovine serum albumin by novel gold nanoparticles capped with amphiphilic sulfonatomethylated calixresorcinarenes. J. Mol. Liq..

[32] Burilov V., Mironova D.A., Ibragimova R.R., Solovieva S.E., Antipin I.S. (2016). Interactions of new bis-ammonium thiacalix[4]arene derivatives in 1,3-*alternate* stereoisomeric form with bovine serum albumin. BioNanoSci.

[33] Dell’Orco D., Lundqvist M., Oslakovic C., Cedervall T., Linse S. (2010). Modeling the time evolution of the nanoparticle-protein corona in a body fluid. PLoS ONE.

[34] Nikkhah S.J., Vandichel M. (2022). Modeling polyzwitterion-based drug delivery platforms: A perspective of the current state-of-the-art and beyond. ACS Eng. Au.

[35] Lee H. (2020). Molecular simulations of PEGylated biomolecules, liposomes, and nanoparticles for drug delivery applications. Pharmaceutics.

[36] Syakaev V.V., Kazakova E.K., Morozova J.E., Shalaeva Y.V., Latypov S.K., Konovalov A.I. (2012). Guest controlled aggregation of amphiphilic sulfonatomethylated calix[4]resorcinarenes in aqueous solutions. J. Colloid Interface Sci..

[37] Mironova D.A., Muslinkina L.A., Syakaev V.V., Morozova J.E., Yanilkin V.V., Konovalov A.I., Kazakova E.K. (2013). Crystal violet dye in complexes with amphiphilic anionic calix[4]resorcinarenes: Binding by aggregates and individual molecules. J. Colloid Interface Sci..

[38] Erfani A., Seaberg J., Aichele C.P., Ramsey J.D. (2020). Interactions between biomolecules and zwitterionic moieties: A review. Biomacromolecules.

[39] Guo S., Shi Y., Liang Y., Liu L., Sun K., Li Y. (2021). Relationship and improvement strategies between drug nanocarrier characteristics and hemocompatibility: What can we learn from the literature. Asian J. Pharm. Sci..

[40] Simak J., De Paoli S. (2017). The effects of nanomaterials on blood coagulation in hemostasis and thrombosis. WIREs Nanomed. Nanobiothechnol..

[41] Olas B., Wachowicz B., Stochmal A., Oleszek W. (2002). Anti-platelet effects of different phenolic compounds from Yucca schidigera Roezl. Bark. Platelets.

[42] Born G.V.R. (1962). Quantitative investigation into the aggregation of blood platelets. J. Physiol..

[43] Mishra V., Heath R.J. (2021). Structural and biochemical features of human serum albumin essential for eukaryotic cell culture. Int. J. Mol. Sci..

[44] Lakowicz J.R. (1999). Principles of Fluorescence Spectroscopy.

[45] Bi S., Sun Y., Qiao C., Zhang H., Liu C. (2009). Binding of several anti-tumor drugs to bovine serum albumin: Fluorescence study. J. Lumin..

[46] Zhao X., Liu R., Chi Z., Teng Y., Qin P. (2010). New insights into the behavior of bovine serum albumin adsorbed onto carbon nanotubes: Comprehensive spectroscopic studies. J. Phys. Chem. B.

[47] Hu Y.J., Ou-Yang Y., Dai C.M., Liu Y., Xiao X.H. (2010). Binding of berberine to bovine serum albumin: Spectroscopic approach. Mol. Biol. Rep..

[48] Ware W.R. (1962). Oxygen quenching of fluorescence in solution: An experimental study of diffusion process. J. Phys. Chem..

[49] Khaibrakhmanova D., Nikiforova A., Sedov I. (2020). Binding constants of substituted benzoic acids with bovine serum albumin. Pharmaceuticals.

[50] Ross P.D., Subramanian S. (1981). Thermodynamics of protein association reactions: Forces contributing to stability. Biochemistry.

[51] Gao S., Holkar A., Srivastava S. (2019). Protein–polyelectrolyte complexes and micellar assemblies. Polymers.

[52] Kuznetsova D.A., Gabdrakhmanov D.R., Lukashenko S.S., Faizullin D.A., Zuev Y.F., Nizameev I.R., Kadirov M.K., Kuznetsov D.M., Zakharova L.Y. (2020). Interaction of bovine serum albumin with cationic imidazolium-containing amphiphiles bearing urethane fragment: Effect of hydrophobic tail length. J. Mol. Liq..

[53] Greenfield N. (2006). Using circular dichroism spectra to estimate protein secondary structure. Nat. Protoc..

[54] Tunstad L.M., Tucker J.A., Dalcanale E., Weiser J., Bryant J.A., Sherman J.C., Helgeson R.C., Knobler C.B., Cram D.C. (1989). Host-guest complexation. 48. Octol building blocks for cavitands and carcerands. J. Org. Chem..

[55] Mironov A.N. (2012). Handbook for Preclinical Drug Trials.

[56] Aguiar J., Carpena P., Molina-Bolívar J.A., Ruiz C.C. (2003). On the determination of the critical micelle concentration by the pyrene 1:3 ratio method. J. Colloid Interface Sci..

