# Supplementary material for: Calix[4]Resorcinarene Carboxybetaines and Carboxybetaine Esters: Synthesis, Investigation of In Vitro Toxicity, Anti-Platelet Effects, Anticoagulant Activity, and BSA Binding Affinities
Source: Int J Mol Sci. 2022 Dec 4;23(23):15298. doi: 10.3390/ijms232315298 (PMC9740030; doi:10.3390/ijms232315298)
Supplement: Supplementary file 1 [file ijms-23-15298-s001.zip › ijms-2028072-supplementary.pdf]

Calix[4]resorcinarene carboxybetaines and carboxybetaine esters: synthesis, investigation of *in vitro* toxicity, anti-platelet effects, anticoagulant activity, and BSA binding affinities

Julia E. Morozova[1], Zuchra R.Gilmullina[2], Alexandra D. Voloshina[1], Anna P. Lyubina[1], Syumbelya K. Amerhanova[1], Victor V. Syakaev[1], Olga B. Babaeva[1], Albina Yu. Ziganshina[1], Timur A. Mukhametzyanov[2], Aleksandr V. Samorodov[3], Michael M. Galagudza[4], Igor S. Antipin[2]*

[1] Arbuzov Institute of Organic and Physical Chemistry, FRC Kazan Scientific Center of RAS, 8 Arbuzov Street, 420088 Kazan, Russia
[2] A.M. Butlerov Chemistry Institute, Kazan Federal University, 18 Kremlyovskaya Str., 420008 Kazan, Russia
[3] Bashkir State Medical University, 3 Lenina St., 450008, Ufa, Bashkortostan, Russia.
[4] Almazov National Medical Research Centre, 190000 Saint Petersburg, Russia
* Correspondence: iantipin54@yandex.ru

Supplementary Materials

Table of content

	page
The synthesis route of calix[4]resorcinarenes **1** and **2**	2
Materials and methods.	2
General procedure for the synthesis of compounds **9** and **10**	2
General procedure for the synthesis of compounds **1** and **2**	3
^{1}H NMR, IR and mass-spectra of compounds **9**, **10**, **1** and **2** (Figures S1-S12).	3-8
^{1}H NMR, ^{13}C NMR, COSY, HSQC, HMBC spectra of compounds **3-6** (Figures S13-S38).	9-22
IR and ESI spectra of compounds **3-6** (Figures S39-S46).	23-26
The pyrene I/III values dependence on the logarithmic concentration of compounds **3-6** (Figure S47).	26
Cytotoxicity and hemolytic activity of **3** – **6** (Table S1, S2).	27
The fluorescence and absorbance spectra of BSA – compound **3-6** solutions (Figures S48-S51).	28-31
DLS data for BSA-macrocycle **3-6** solutions (Table S3).	32
Reference	32

1. The synthesis route of calix[4]resorcinarenes 1 and 2:

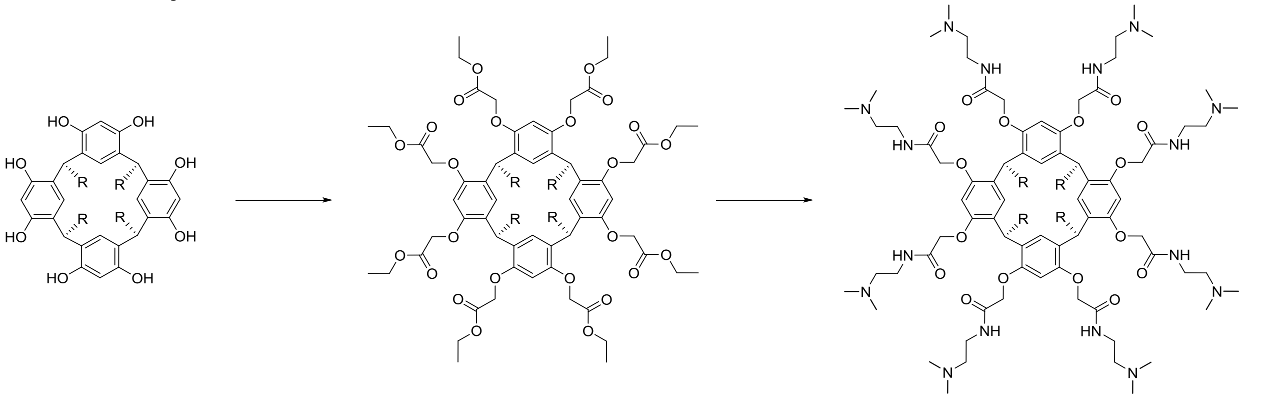

7 R = C_5H_{11}
8 R = $C_{11}H_{23}$

9 R = C_5H_{11}
10 R = $C_{11}H_{23}$

1 R = C_5H_{11}
2 R = $C_{11}H_{23}$

Materials and methods.

Macrocycles **7** and **8** were synthesized as described in ref. [1]. The spectral characteristics of the macrocycles are in accordance to the literature data. Ethyl 2-bromoacetate and N,N-dimethylethylenediamine obtained from Sigma-Aldrich.

^{1}H NMR spectra were performed on a MSL-400, Bruker AVANCE(III)-500 and Bruker AVANCE-600 spectrometers. The chemical shifts were reported relative to the residual solvent peaks as internal standards. IR spectra were recorded with Vector 22 Spectrometer in KBr pellets. Maldi-TOF mass spectra were recorded on an Ultralex III TOF/TOF mass spectrometer (Bruker Daltonic, Bremen, Germany) operated in the linear and reflection mode. A Nd:YAG laser, λ = 355 nm was used. The data were processed using the FlexAnalysis 3.0 software (Bruker Daltonics, Bremen, Germany).

General procedure for the synthesis of compounds 9 and 10

The compound **7** (20.12 g, 26.0 mmol) or **8** (20.00 g, 18.00 mmol) were dissolved in 200 ml of dry acetonitrile. Then an anhydrous K_2CO_3 (43.05 g, 0.31 mol or 29.81 g, 0.216 mol, respectively) was added and reaction mixture was stirred at rt for 0.5 h. Then ethyl 2-bromoacetate (23 mL, 0.208 mol or 16 mL, 0.144 mol, respectively) was added and the reaction mixture was stirred and heated at 60 °C for 150 h in an inert atmosphere. After cooling to rt the precipitate was filtered and filtrate was evaporated under reduced pressure. After drying the macrocycles were obtained as amorphous compounds.

2,8,14,20-Tetrapentylpentacyclo[19.3.1.1^{3,7}.1^{9,13}.1^{15,19}]-octacosa-1(25),3,5,7(28),9,11,13(27),15,17,19(26),21,23-dodecaen-4,6,10,12,16,18,22,24-octakis(ethyl[2-methoxy]acetate) **9**.Yield 34.8 g (91.8 %); ^{1}H NMR (400 MHz, $CDCl_3$): δ = 0.83 (t, 12H, J_{HH} 6 MHz, $CH_2(CH_2)_3$*CH_3*), 1.26 (m, 48H; CH_2*$(CH_2)_3$*CH_3, OCH_2*CH_3*), 1.85 (q, 8H, J_{HH} 8 MHz, *CH_2*$(CH_2)_3CH_3$), 4.25 (m, 32H; OCH_2, O*CH_2*CH_3), 4.59 (t, J_{HH} 8 MHz, 4H; CH), 6.22 (s, 4H; ArH), 6.61 (s, 4H; ArH). IR: ν̃ = 2931 (CH), 2860 (CH), 1761 (C=O), 1612 (C=C_{Ar}), 1586 (C=C_{Ar}), 1500 (C=C_{Ar}), 1306 (C-O-C), 1201 (C-O-C), 1079 (C-O-C). MS (Maldi-TOF): m/z calcd for $C_{80}H_{112}O_{24}$: 1457.7; found 1496.9 $[M+K]^+$; elemental analysis calcd (%) for $C_{80}H_{112}O_{24}$: C 65.91, H 7.74; found: C 64.70, H 7.54.

2,8,14,20-Tetraundecylpentacyclo[19.3.1.1^{3,7}.1^{9,13}.1^{15,19}]-octacosa-1(25),3,5,7(28),9,11,13(27),15,17,19(26),21,23-dodecaen-4,6,10,12,16,18,22,24-octakis(ethyl[2-methoxy]acetate) **10**.Yield 29.9 g (92.6 %); ^{1}H NMR (600 MHz, $CDCl_3$): δ = 0.86 (t, 12H, J_{HH} 6 MHz, $CH_2(CH_2)_9$*CH_3*), 1.22-1.27 (m, 96H; CH_2*$(CH_2)_9$*CH_3, -OCH_2*CH_3*), 1.84 (q, 8H, J_{HH} 8

MHz, $CH_2(CH_2)_9CH_3$), 4.20 (q, 16H, J_{HH} 8 MHz; -O$CH_2$$CH_3$), 4.25 (s, 16H; OCH_2), 4.58 (t, J_{HH} 8 MHz, 4H; CH), 6.22 (s, 4H; ArH), 6.60 (s, 4H; ArH). IR: $\tilde{\nu}$ = 2925 (CH), 2854 (CH), 1762 (C=O), 1612 ($C=C_{Ar}$), 1587 ($C=C_{Ar}$), 1501 ($C=C_{Ar}$), 1305 (C-O-C), 1205 (C-O-C), 1082 (C-O-C). MS (Maldi-TOF): m/z calcd for $C_{104}H_{160}O_{24}$: 1794.1; found 1832.1 $[M+K]^+$; elemental analysis calcd (%) for $C_{104}H_{160}O_{24}$: C 69.91, H 8.99; found: C 69.03, H 8.68.

General procedure for the synthesis of compounds 1 and 2

The compound **9** (20.00 g, 13.7 mmol) or **10** (24.26 g, 13.5 mmol) were dissolved in 40 ml of N,N-dimethylethylenediamine, and reaction mixture was stirred at rt for 0.5 h in an inert atmosphere. Then reaction mixture was stirred and heated at 60 °C for 62 h in an inert atmosphere. After cooling to rt the precipitate was filtered and washed by dioxane (100 mL), and dried under reduced pressure. After drying the macrocycles were obtained as white solids.

2,8,14,20-Tetrapentylpentacyclo[19.3.1.1^{3,7}.1^{9,13}.1^{15,19}]-octacosa-1(25),3,5,7(28),9,11,13(27),15,17,19(26),21,23-dodecaen-4,6,10,12,16,18,22,24-octakis(N-(2-(dimethylamino)ethyl)-2-methoxyacetamide) **1**. M.p. 185-188 °C. Yield 17.1 g (69.6 %); ^{1}H NMR (500 MHz, D_2O): δ = 0.66 (br s, 12H, $CH_2(CH_2)_3\mathit{CH_3}$), 1.24-1.13 (m, 24H; $CH_2\mathit{(CH_2)_3}CH_3$), 1.69 (br s, 8H, $\mathit{CH_2}(CH_2)_3CH_3$), 2.13 (s, 48H, $N(CH_3)_2$), 2.40 (s, 16H, NCH_2), 3.35-3.26 (m, 16H, NCH_2), 4.27 (s, 16H, OCH_2), 4.57 (br s, 4H; CH), 6.55 (s, 4H; ArH). IR: $\tilde{\nu}$ = 3411 (NH), 3325 (NH), 2932 (CH), 2859 (CH), 2770 (CH), 1686 (Amid I), 1529 (Amid II), 1611 ($C=C_{Ar}$), 1585 ($C=C_{Ar}$), 1500 ($C=C_{Ar}$). MS (Maldi-TOF): m/z calcd for $C_{96}H_{160}N_{16}O_{16}$: 1794.4; found 1794.8 $[M]^+$; elemental analysis calcd (%) for $C_{96}H_{160}N_{16}O_{16}$: C 64.26, H 8.99, N 12.49; found: C 64.27, H 8.72, N 12.41.

2,8,14,20-Tetraundecylpentacyclo[19.3.1.1^{3,7}.1^{9,13}.1^{15,19}]-octacosa-1(25),3,5,7(28),9,11,13(27),15,17,19(26),21,23-dodecaen-4,6,10,12,16,18,22,24-octakis(N-(2-(dimethylamino)ethyl)-2-methoxyacetamide) **2**. M.p. 130 °C. Yield 17.8 g (62.0 %); ^{1}H NMR (600 MHz, $CDCl_3$): δ = 0.86 (t, 12H, J_{HH} 6 MHz, $CH_2(CH_2)_9\mathit{CH_3}$), 1.22 (m, 72H; $CH_2\mathit{(CH_2)_9}CH_3$), 1.83 (br s, 8H, $\mathit{CH_2}(CH_2)_9CH_3$), 2.60 (s, 48H, $N(CH_3)_2$), 2.96 (s, 16H, NCH_2), 3.61 (br s, 16H, NCH_2), 4.30 (br s, 16H, OCH_2), 4.53 (br s, 4H; CH), 6.48 (s, 4H; ArH), 7.78 (s, 4H; ArH). IR: $\tilde{\nu}$ = 3411 (NH), 3379 (NH), 3323 (NH), 2925 (CH), 2853 (CH), 2768 (CH), 1685 (Amid I), 1534 (Amid II), 1500 ($C=C_{Ar}$). MS (Maldi-TOF): m/z calcd for $C_{96}H_{160}N_{16}O_{16}$: 2129.6; found 2128.0 $[M]^+$; elemental analysis calcd (%) for $C_{120}H_{208}N_{16}O_{16}$: C 67.63, H 9.84, N 10.52; found: C 67.90, H 9.80, N 10.60.

^{1}H NMR, IR and mass-spectra of macrocycles 9, 10, 1 and 2.

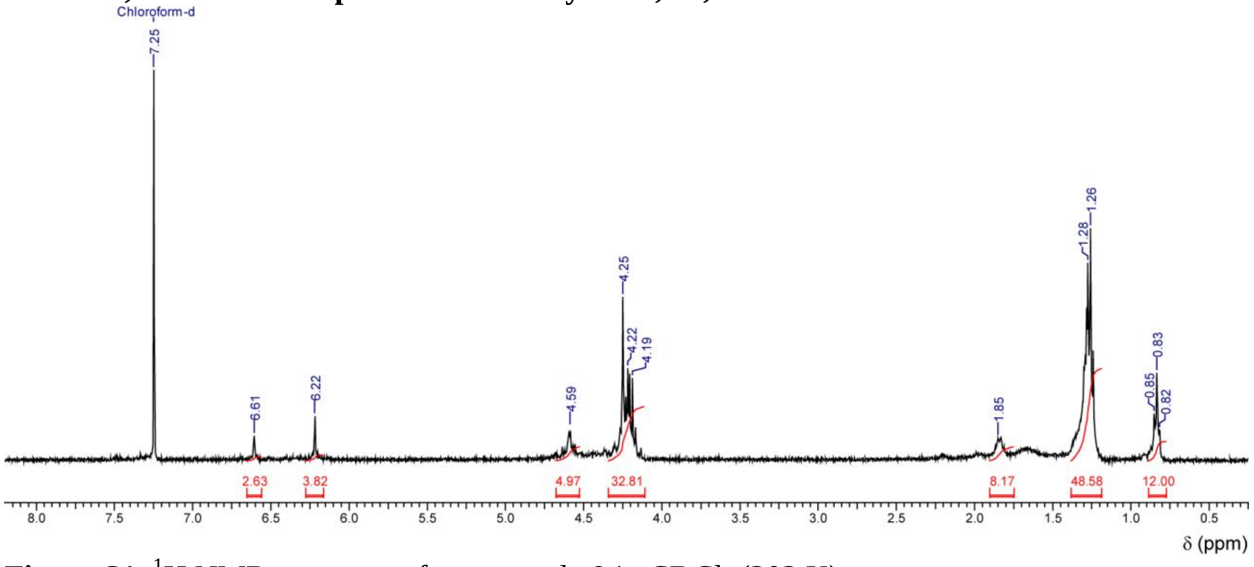

Figure S1. ^{1}H NMR spectrum of macrocycle **9** in $CDCl_3$ (303 K).

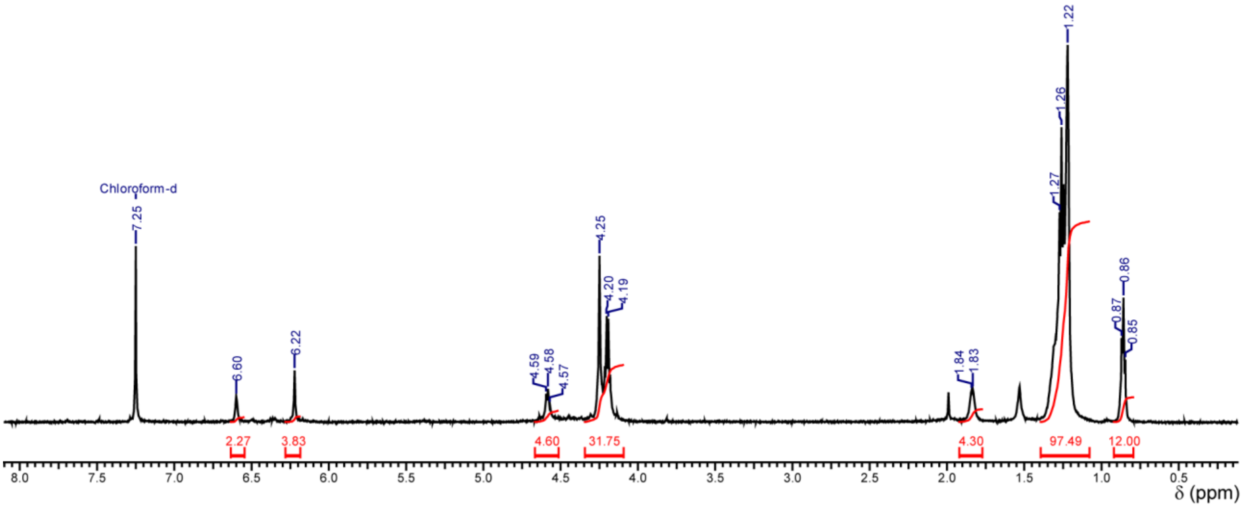

Figure S2. ^{1}H NMR spectrum of macrocycle **10** in $CDCl_3$ (303 K).

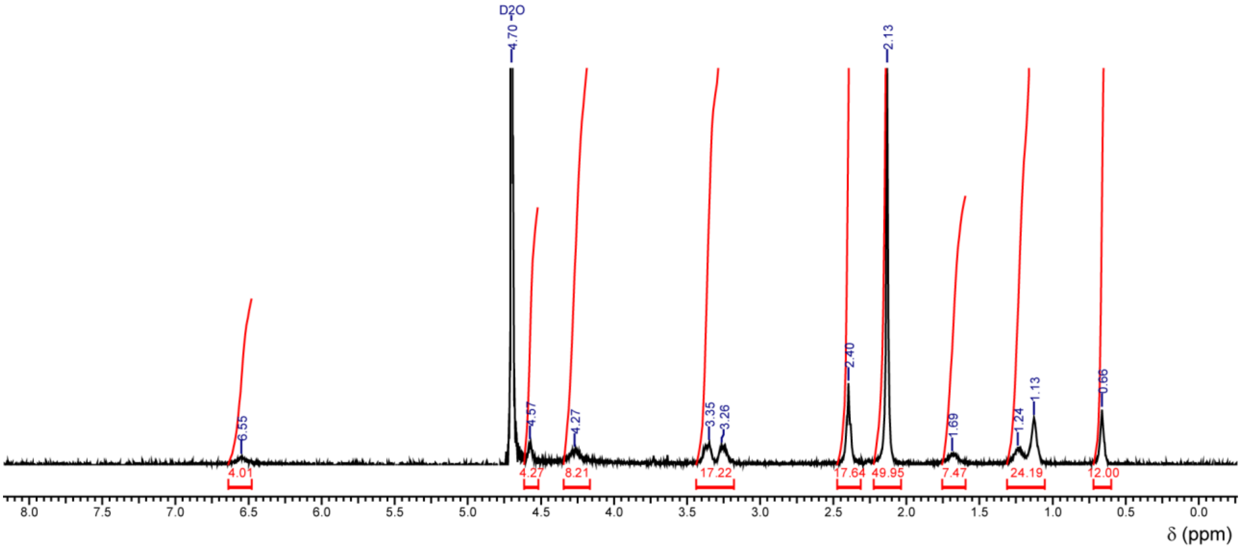

Figure S3. ^{1}H NMR spectrum of macrocycle **1** in D_2O (303 K).

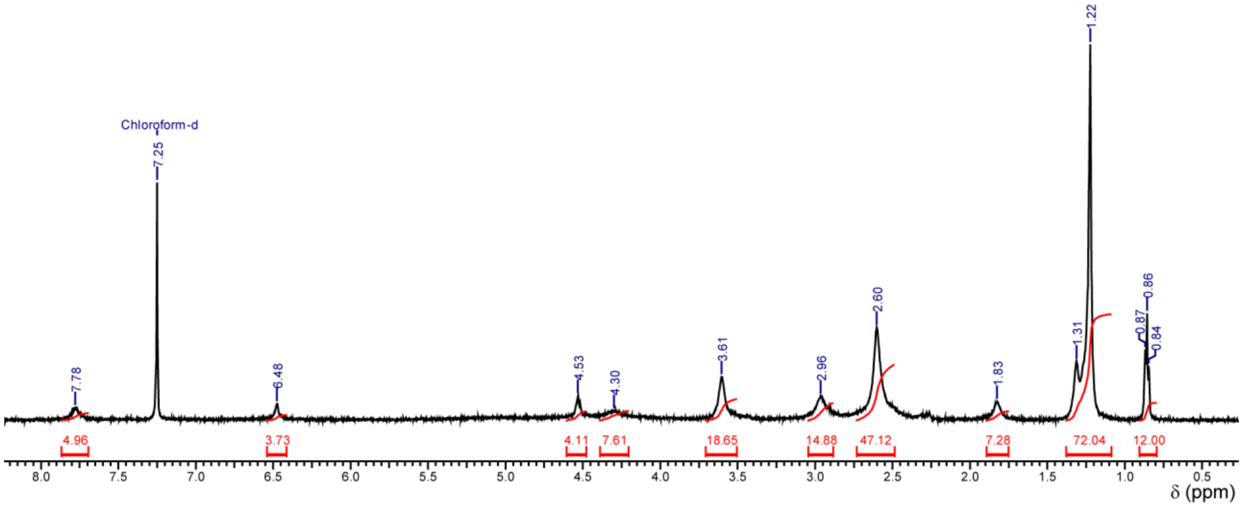

Figure S4. ^{1}H NMR spectrum of macrocycle **2** in $CDCl_3$ (303 K).

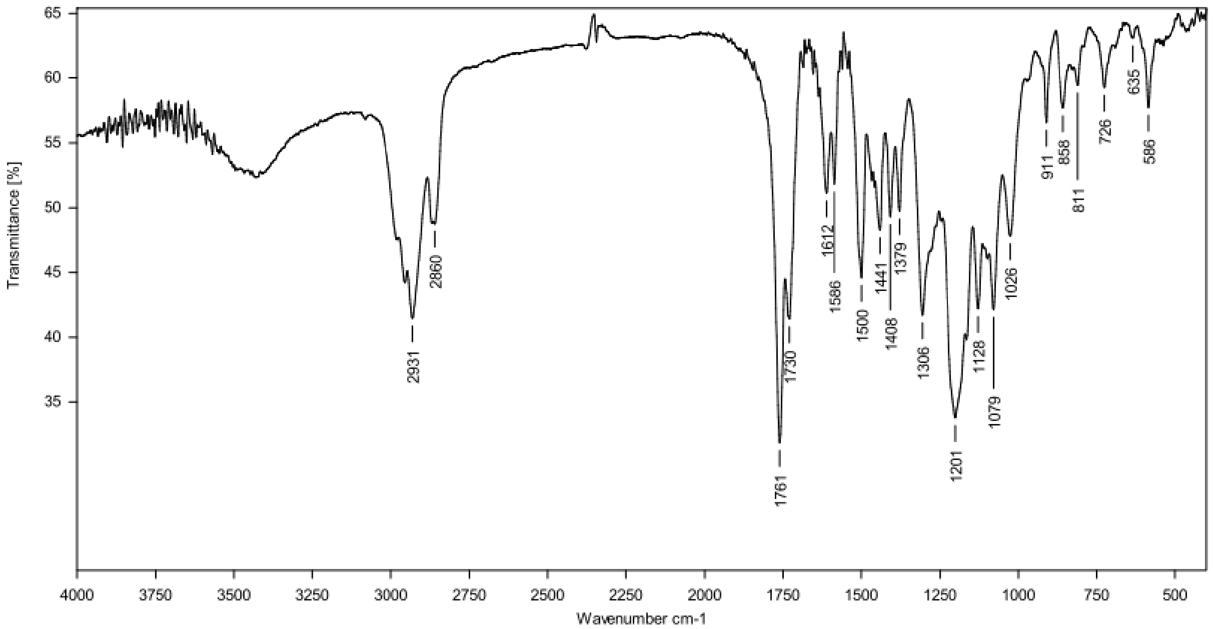

Figure S5. IR spectrum of macrocycle **9** (KBr).

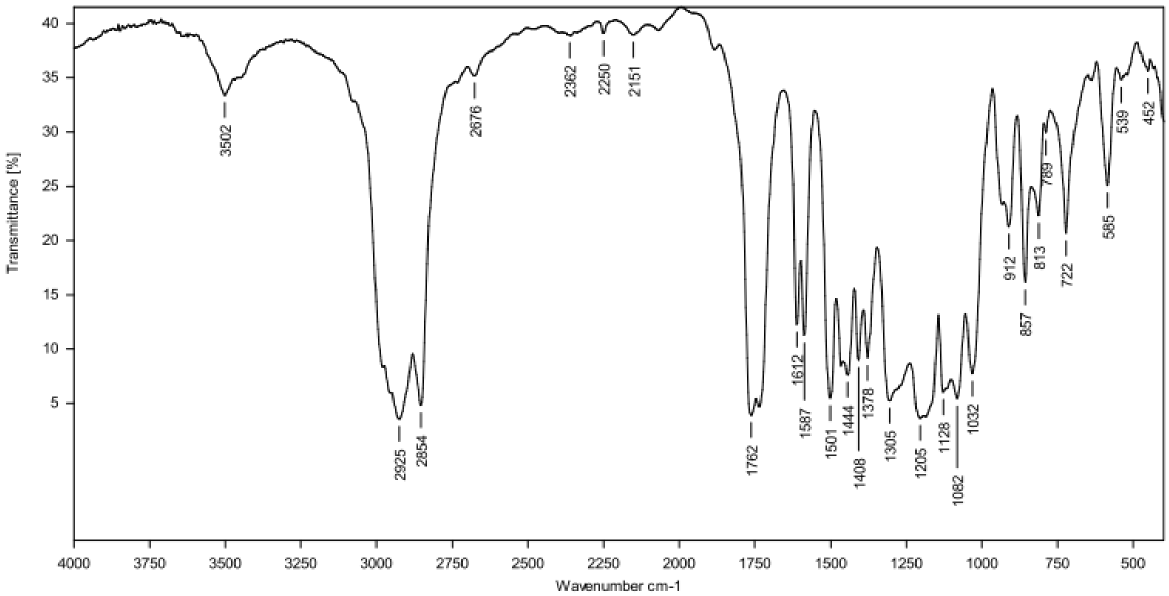

Figure S6. IR spectrum of macrocycle **10** (KBr).

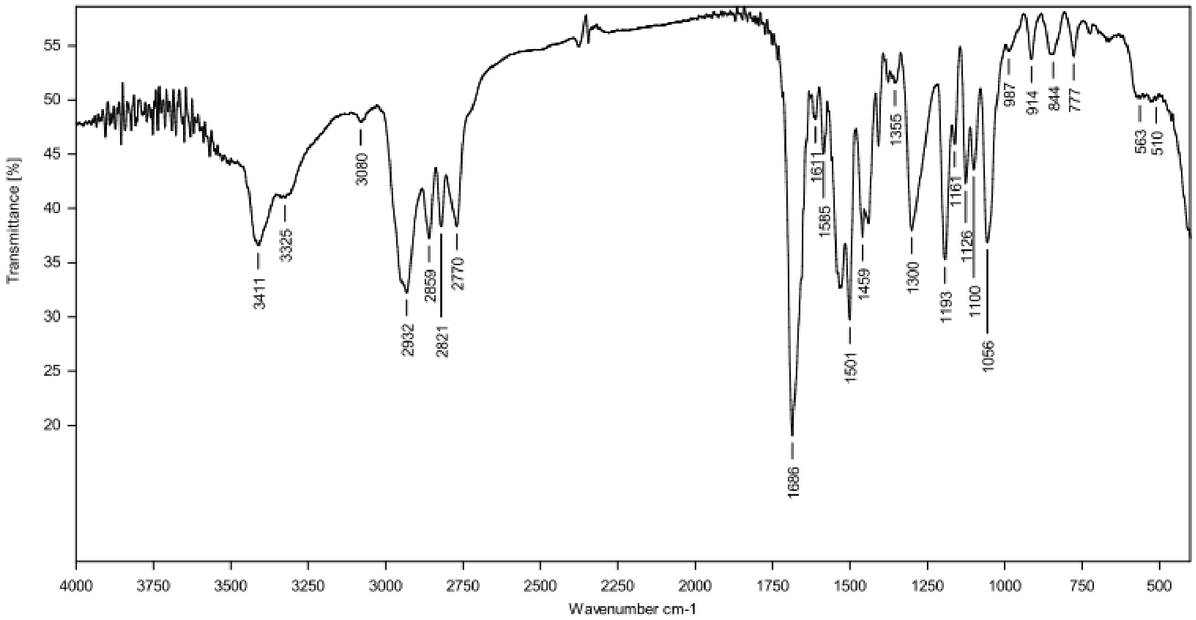

Figure S7. IR spectrum of macrocycle **1** (KBr).

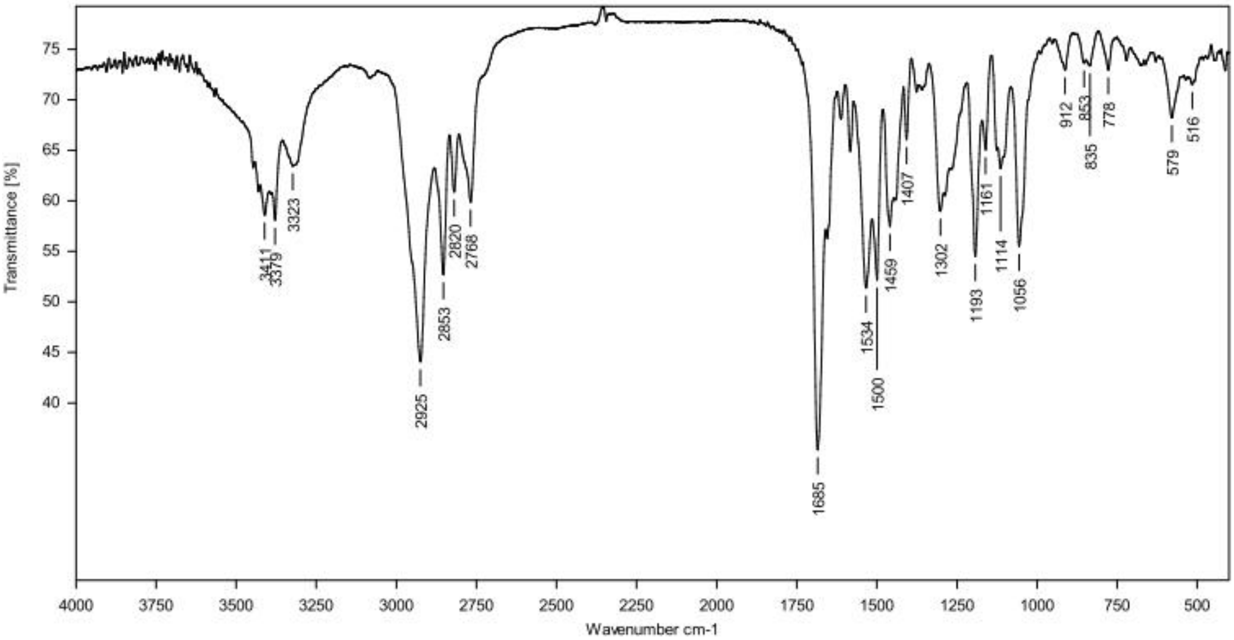

Figure S8. IR spectrum of macrocycle **2** (KBr).

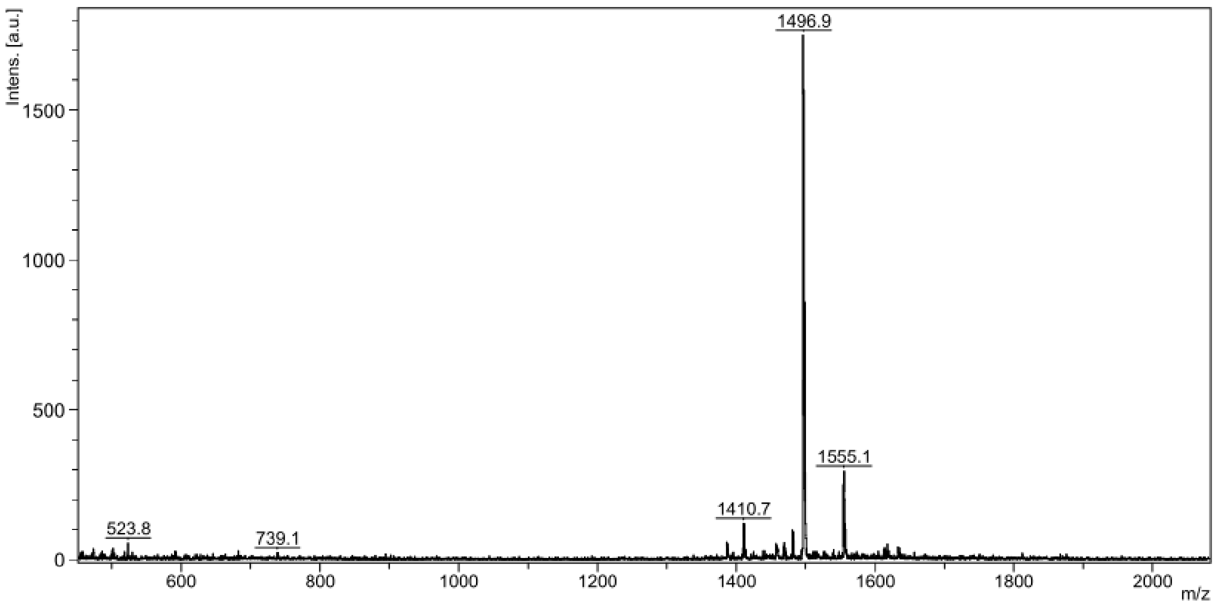

Figure S9. Mass-spectrum (Maldi-TOF) of macrocycle **9.**

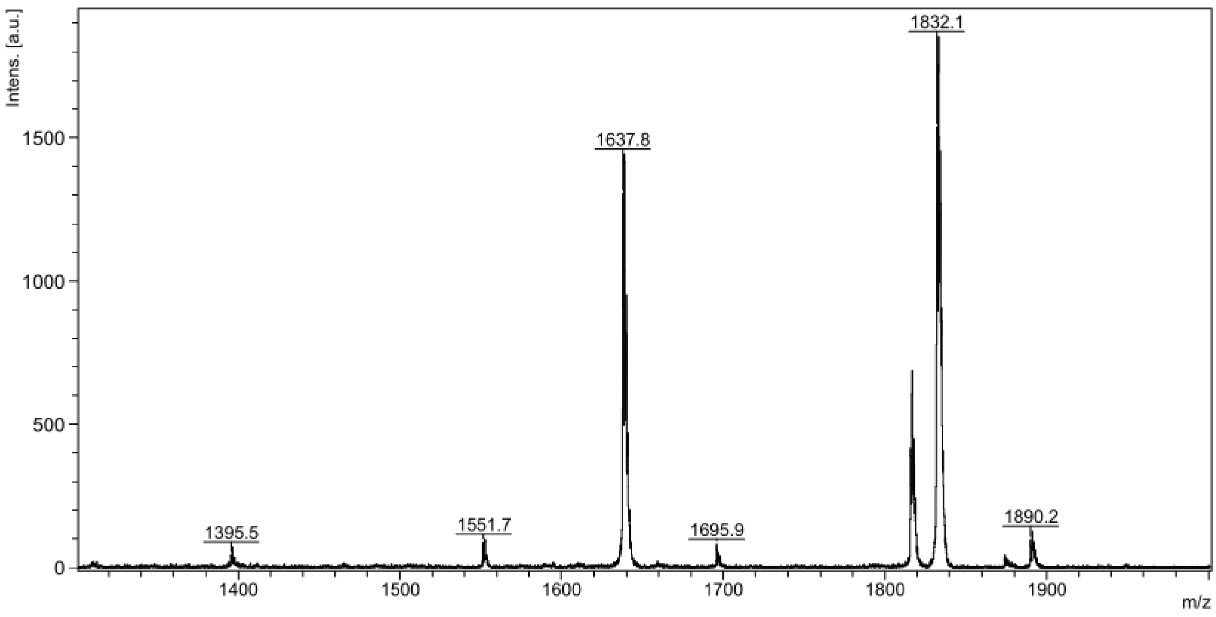

Figure 10. Mass-spectrum (Maldi-TOF) of macrocycle **10.**

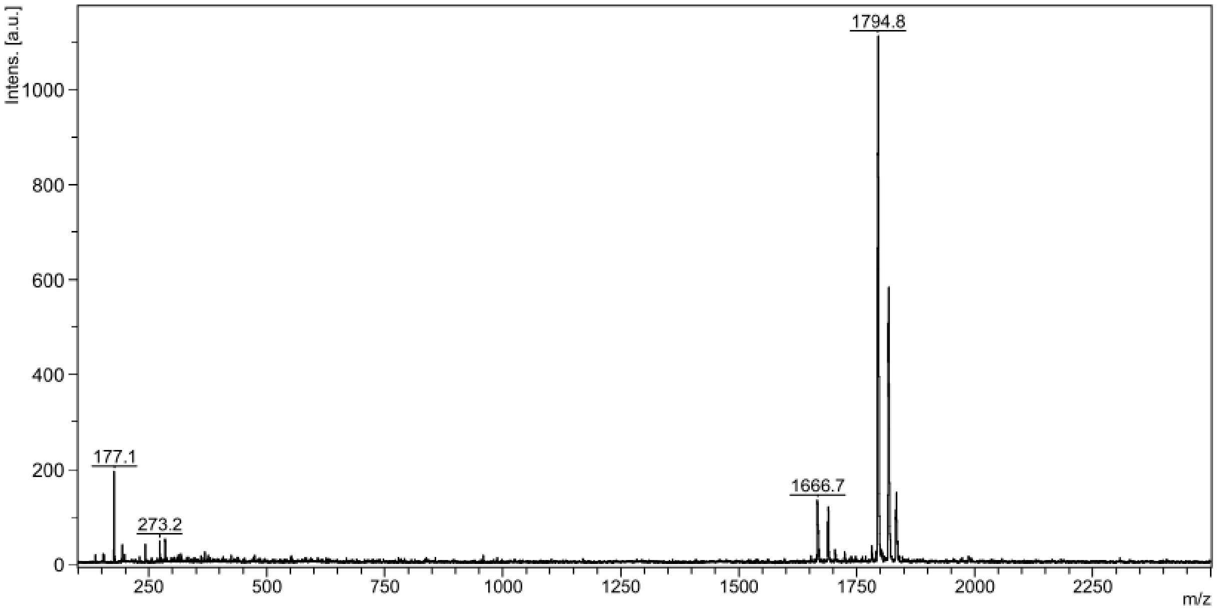

Figure S11. Mass-spectrum (Maldi-TOF) of macrocycle **1.**

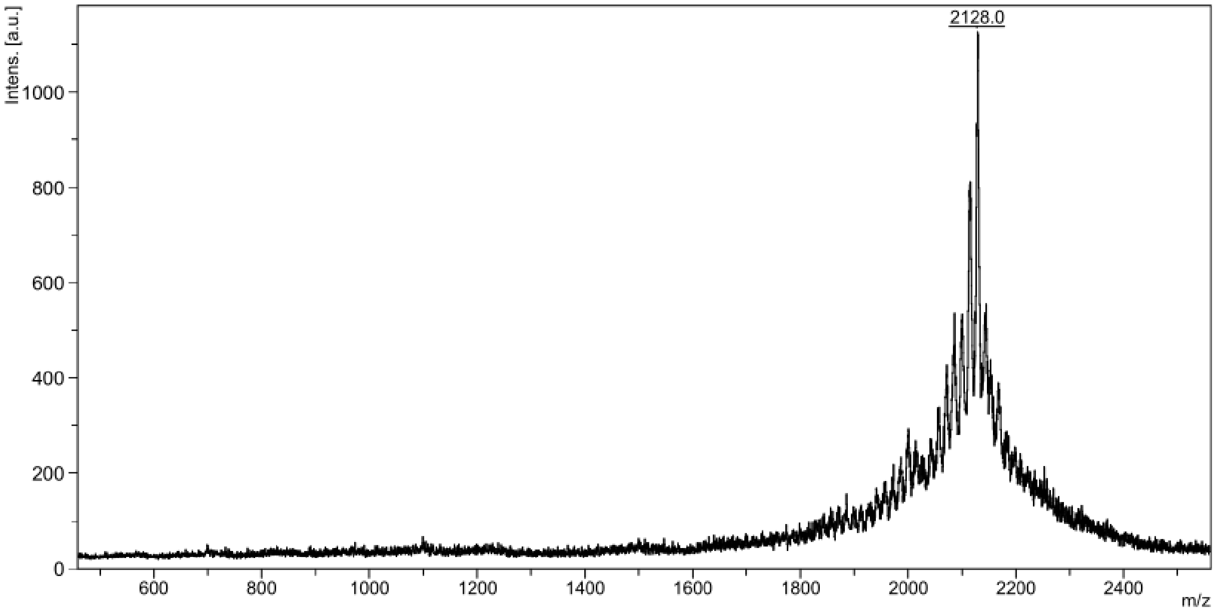

Figure S12. Mass-spectrum (Maldi-TOF) of macrocycle **2.**

^{1}H NMR, ^{13}C NMR, COSY, HSQC, HMBC spectra of compounds 3-6

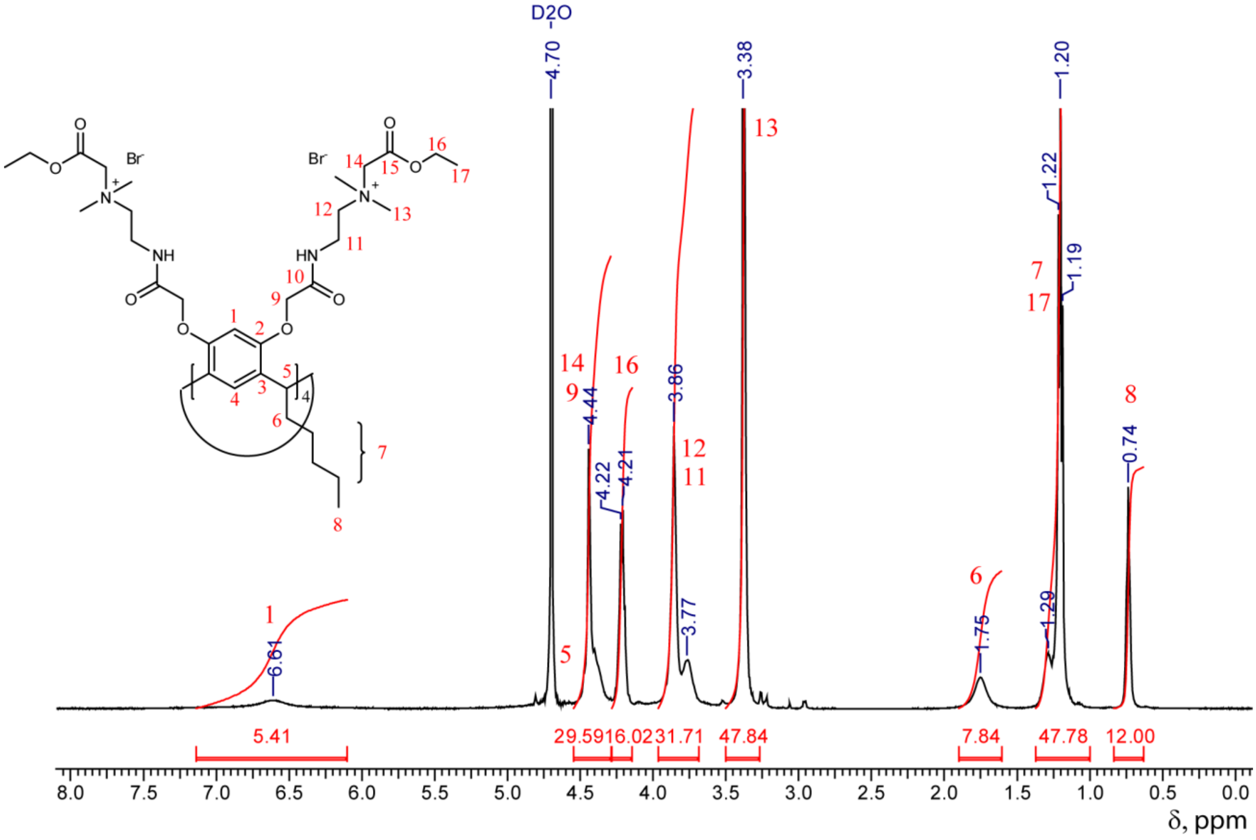

Figure S13.^{1}H NMR spectrum of macrocycle **3** in D_2O (303 K).

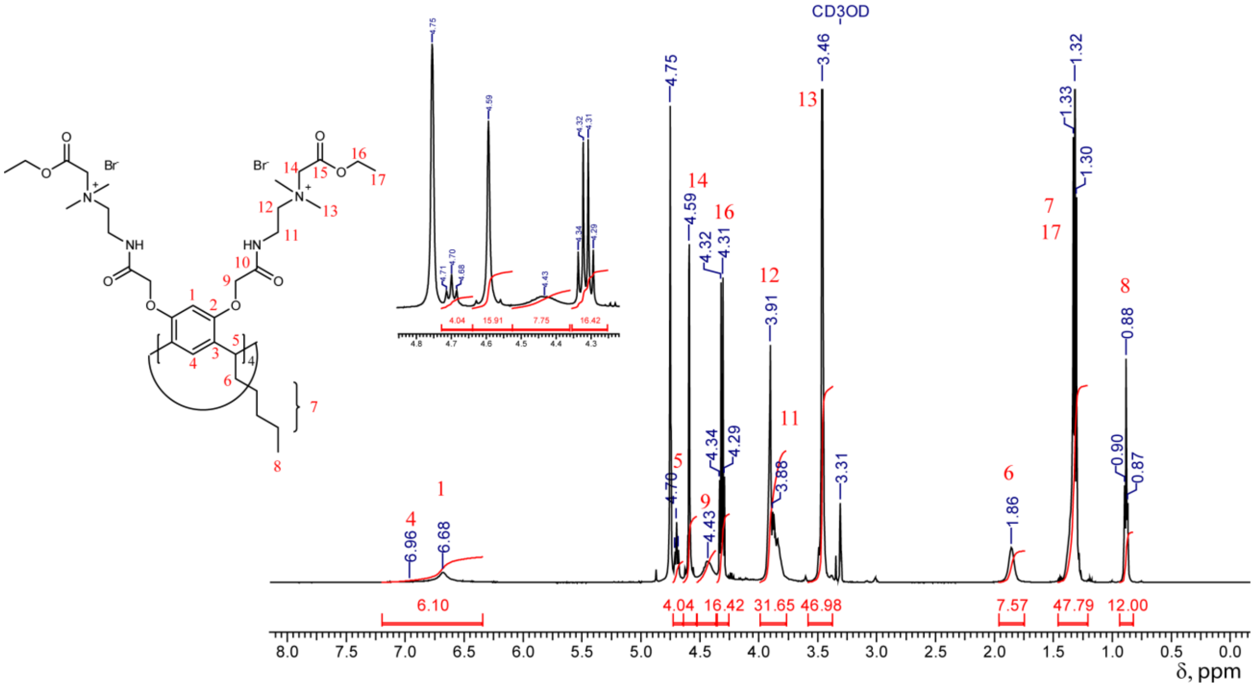

Figure S14.^{1}H NMR spectrum of macrocycle **3** in CD_3OD (303 K).

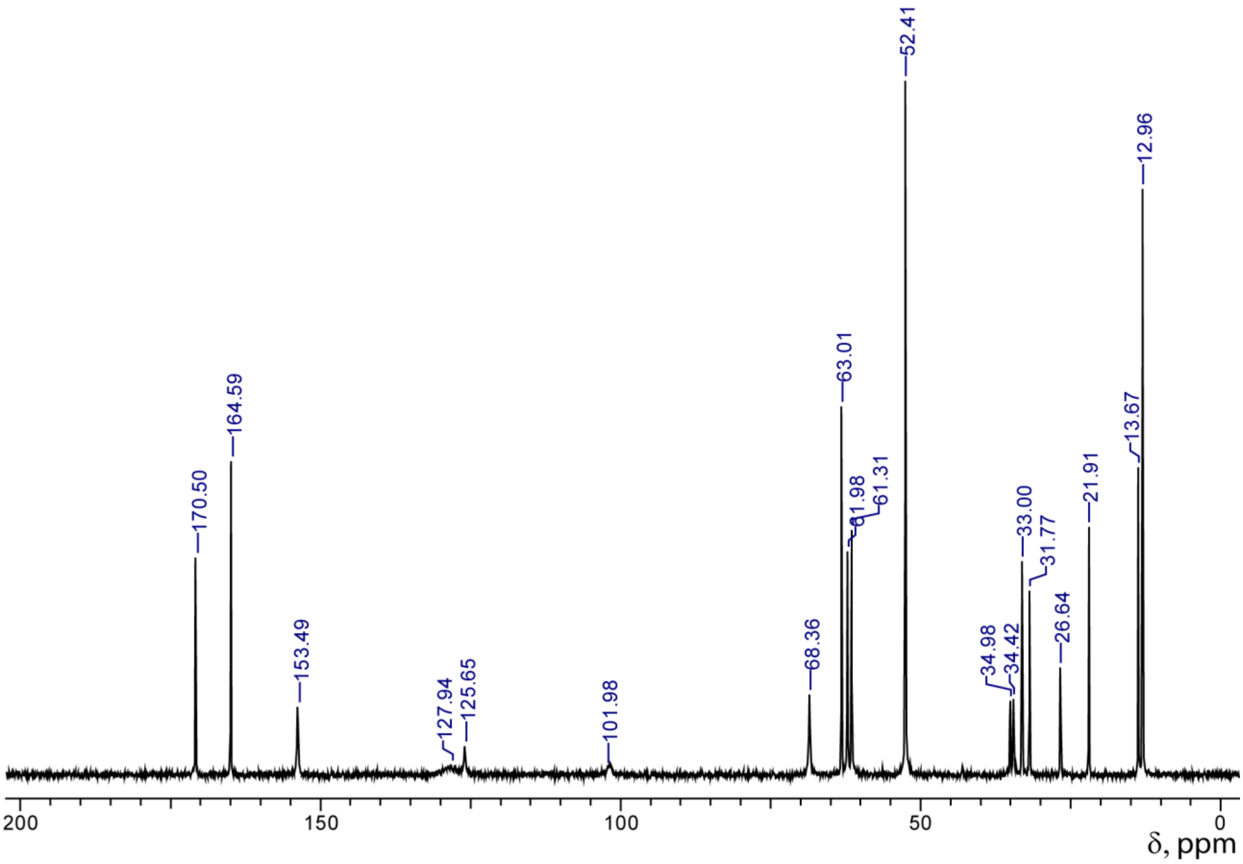

Figure S15.^{13}C NMR spectrum of macrocycle **3** in D_2O (303 K).

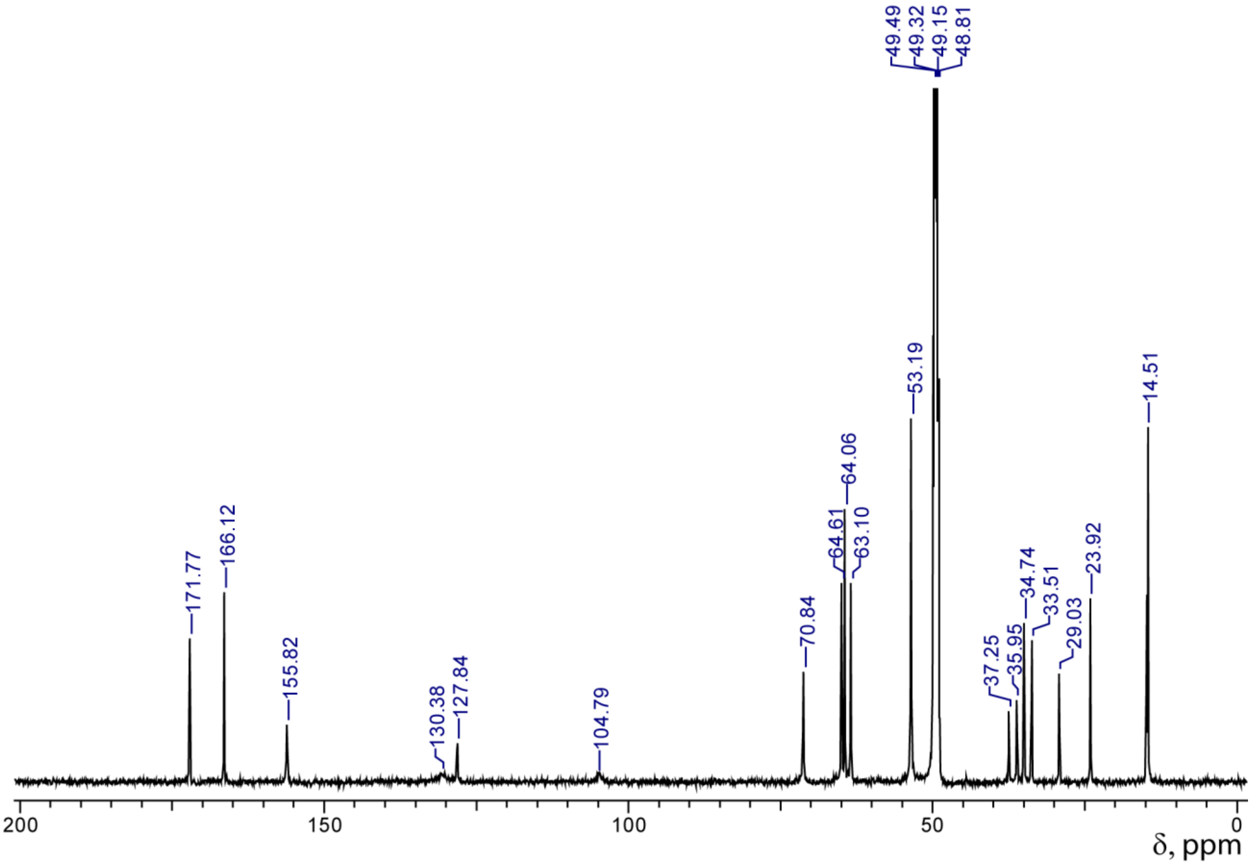

Figure S16.^{13}C NMR spectrum of macrocycle **3** in CD_3OD (303 K).

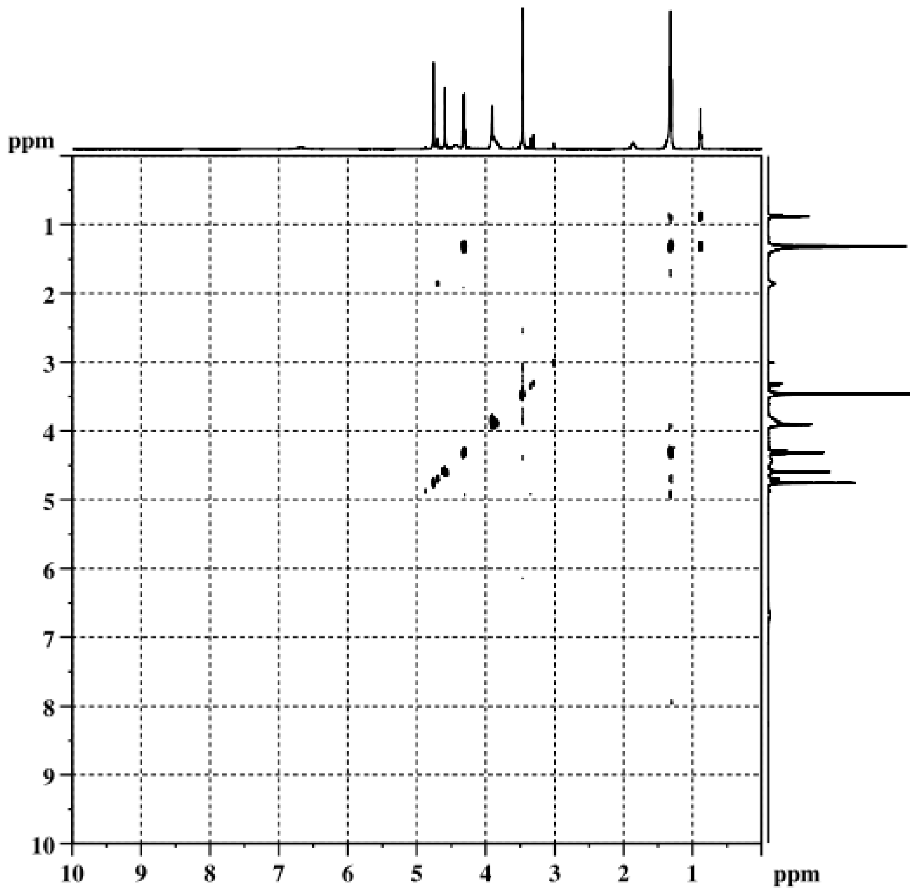

Figure S17. COSY spectrum of macrocycle **3** in CD_3OD (303 K).

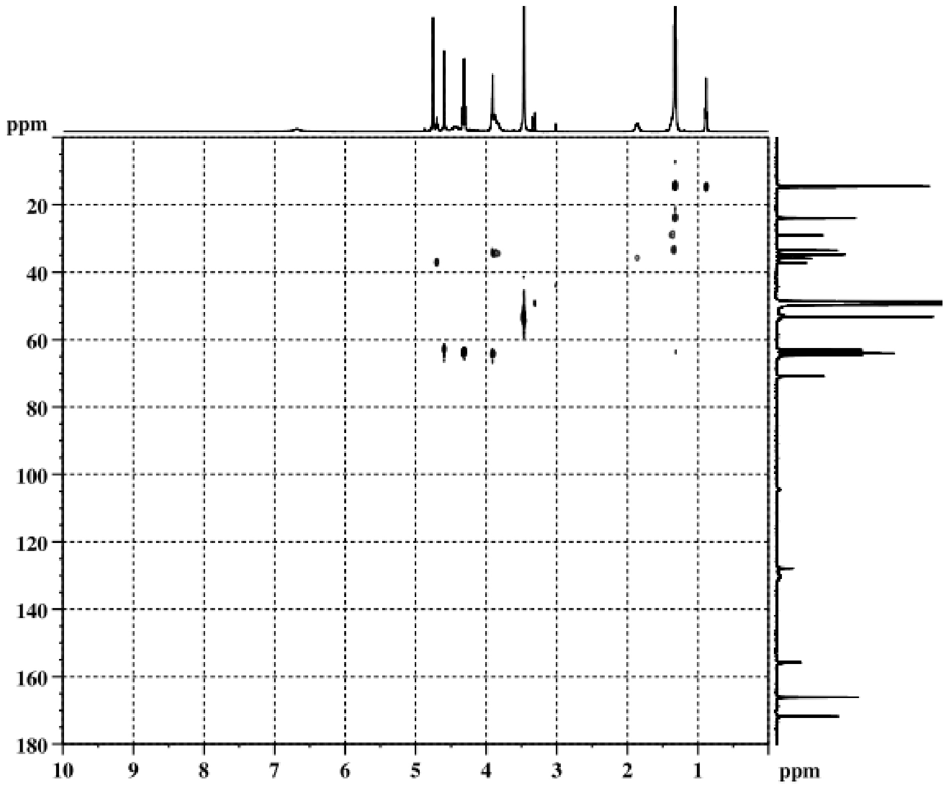

Figure S18. HSQC spectrum of macrocycle **3** in CD_3OD (303 K).

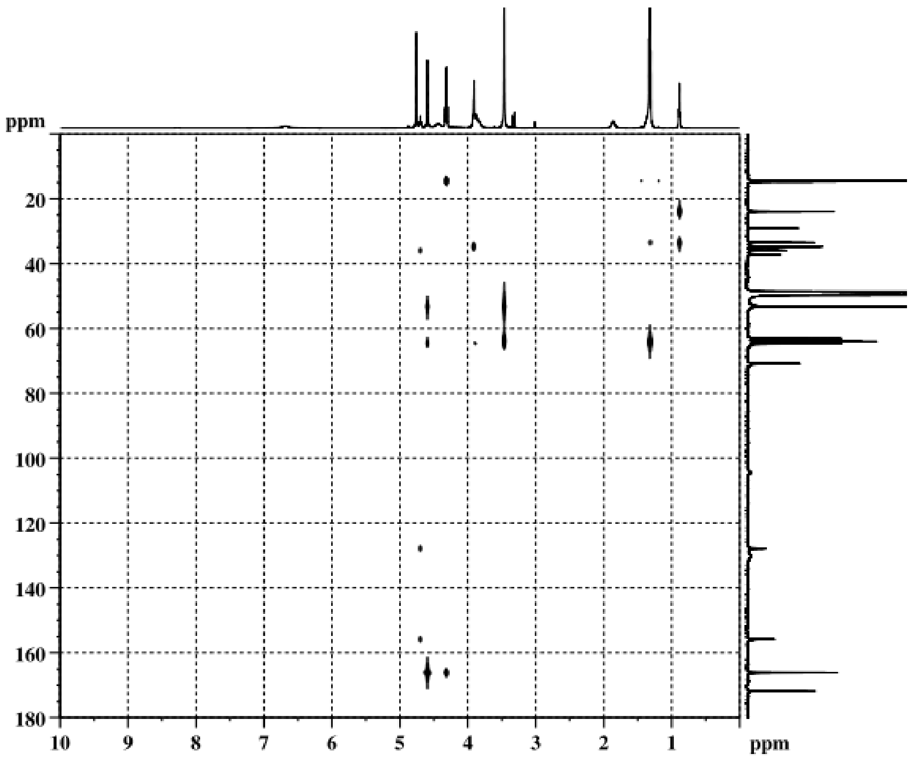

Figure S19. HMBC spectrum of macrocycle **3** in CD_3OD (303 K).

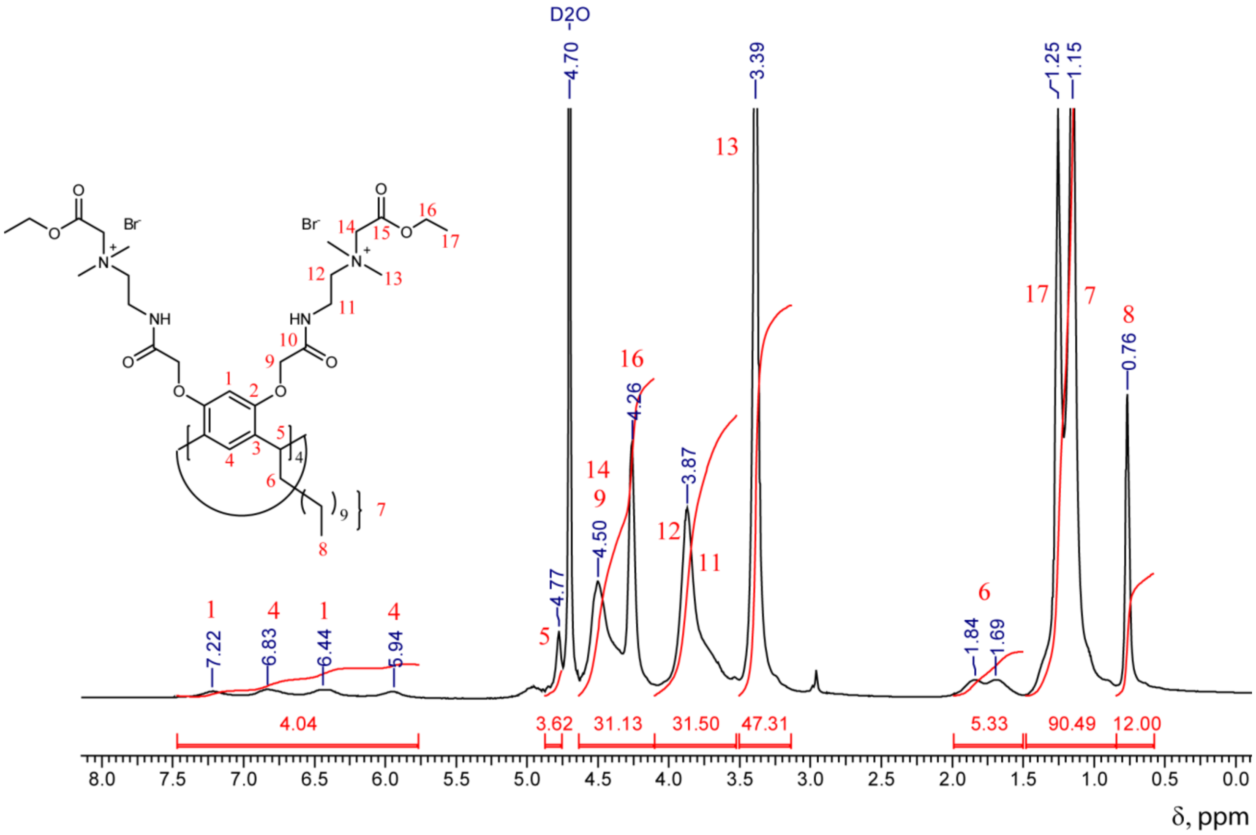

Figure S20.^{1}H NMR spectrum of macrocycle **4** in D_2O (303 K).

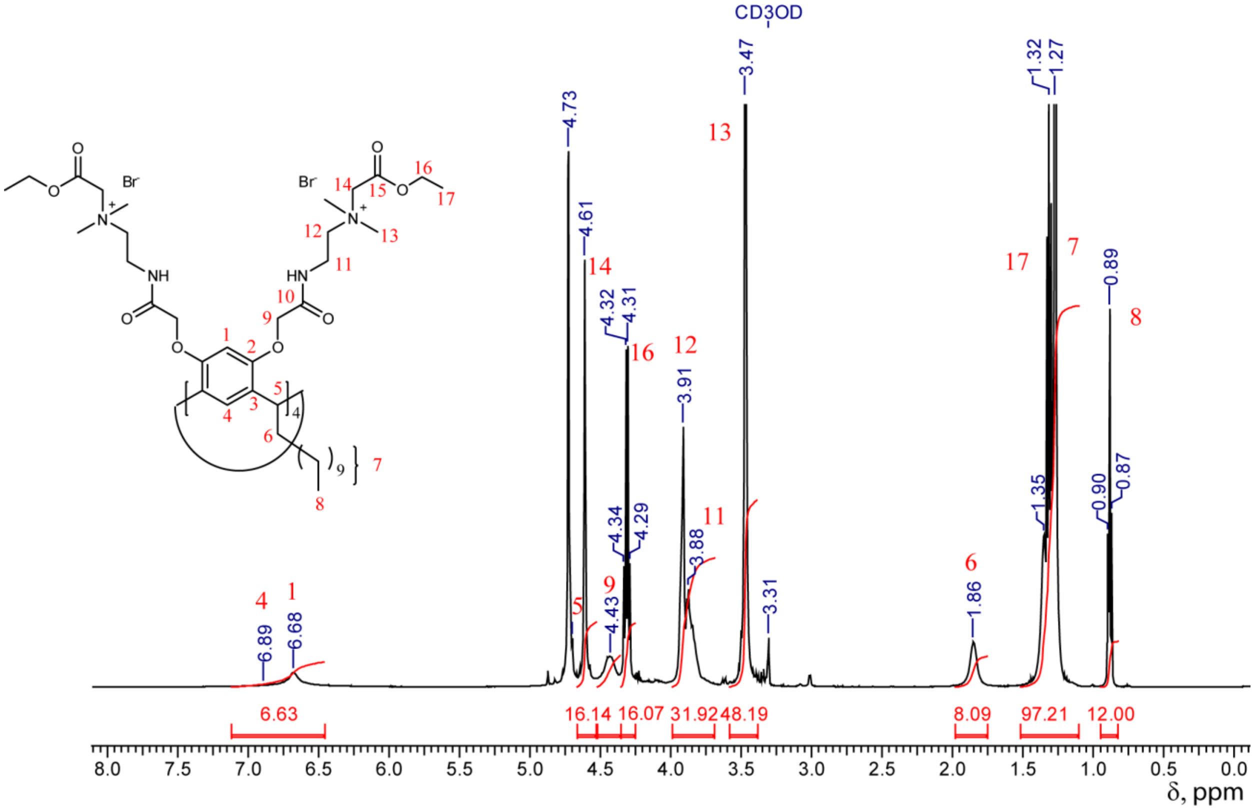

Figure S21.^{1}H NMR spectrum of macrocycle **4** in CD_3OD (303 K).

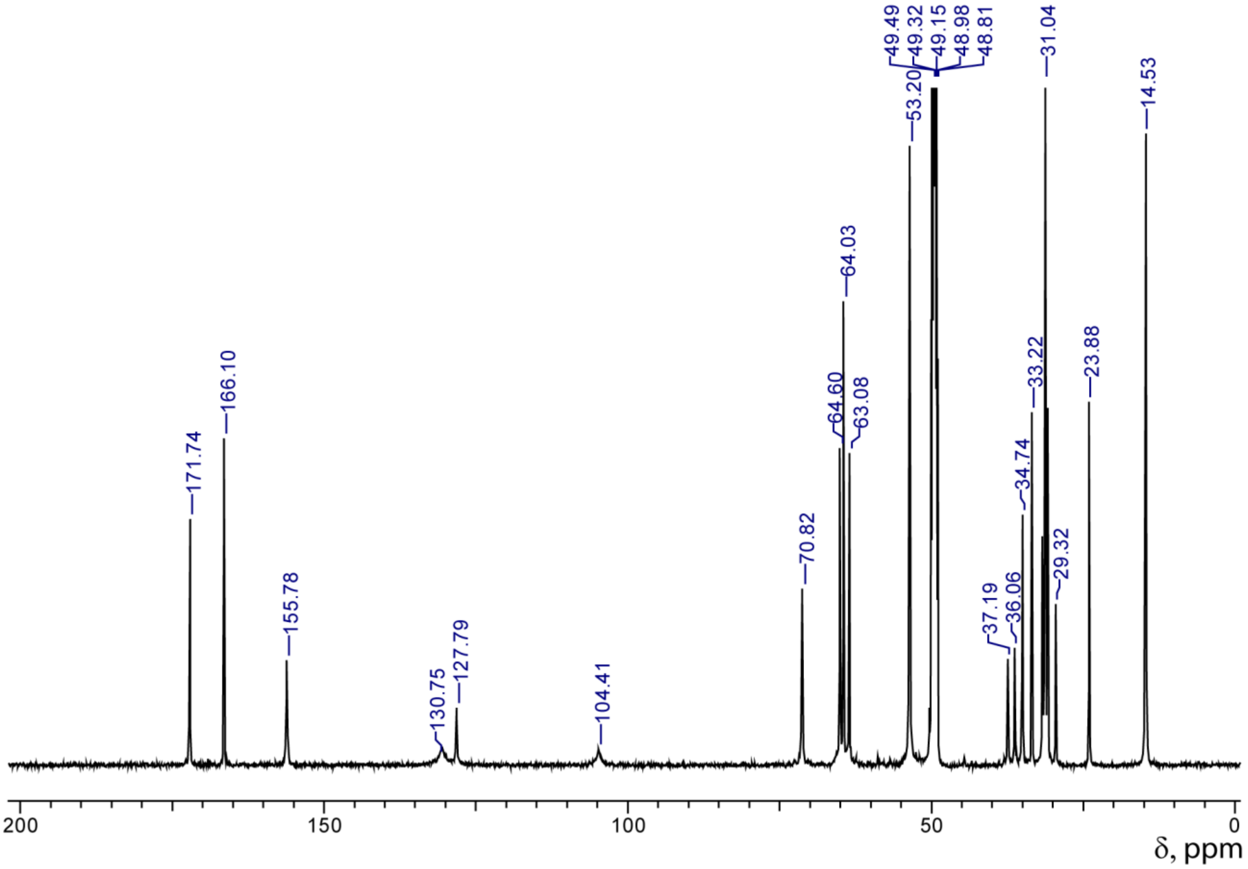

Figure S22. ^{13}C NMR spectrum of macrocycle **4** in CD_3OD (303 K).

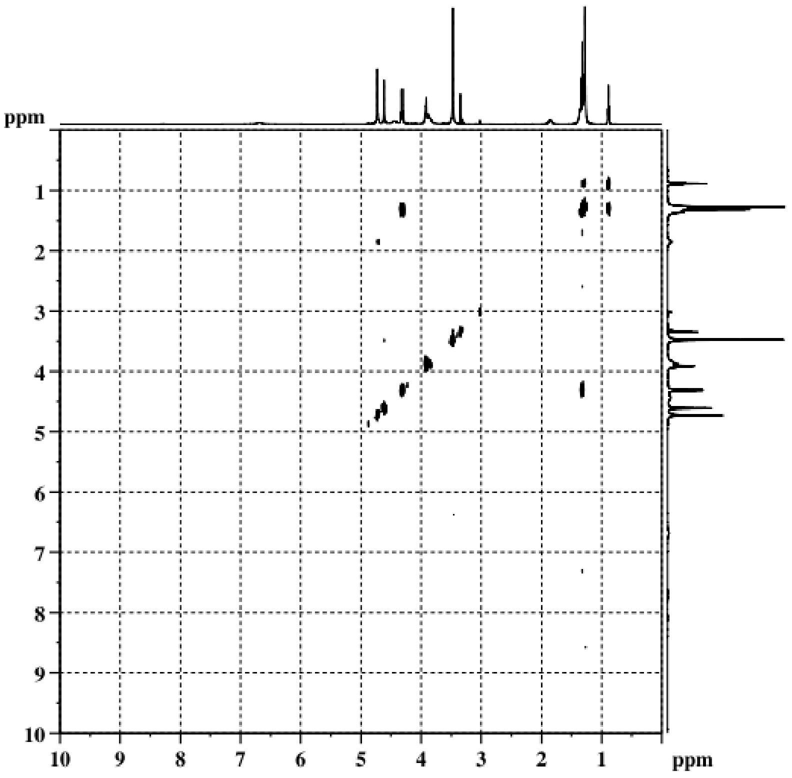

Figure S23. COSY spectrum of macrocycle **4** in CD_3OD (303 K).

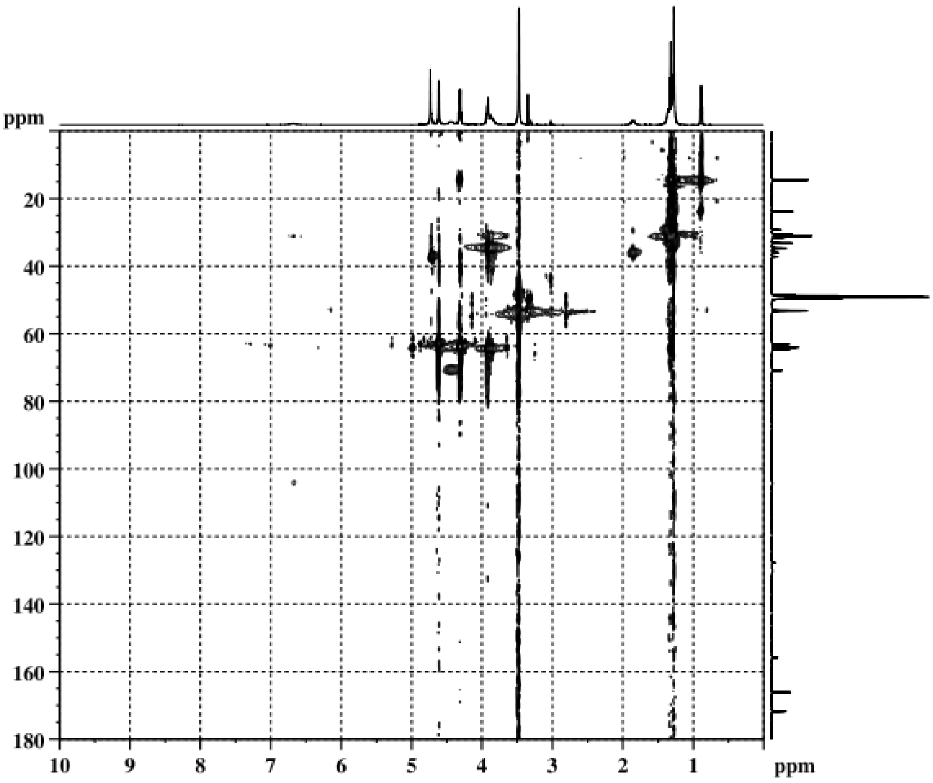

Figure S24. HSQC spectrum of macrocycle **4** in CD_3OD (303 K).

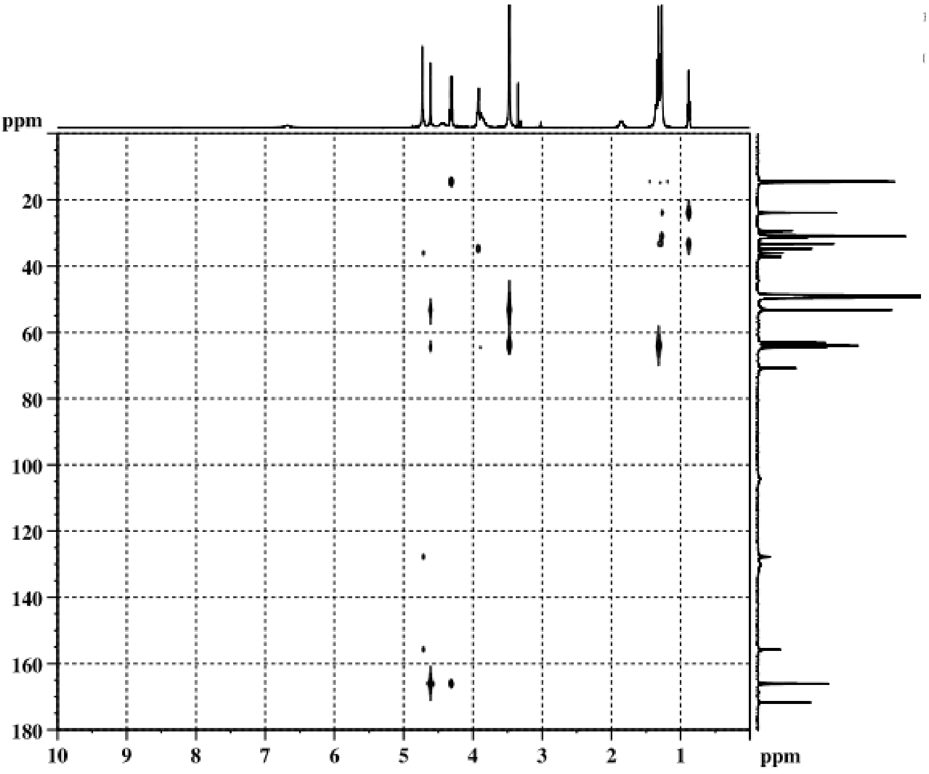

Figure S25. HMBC spectrum of macrocycle **4** in CD_3OD (303 K).

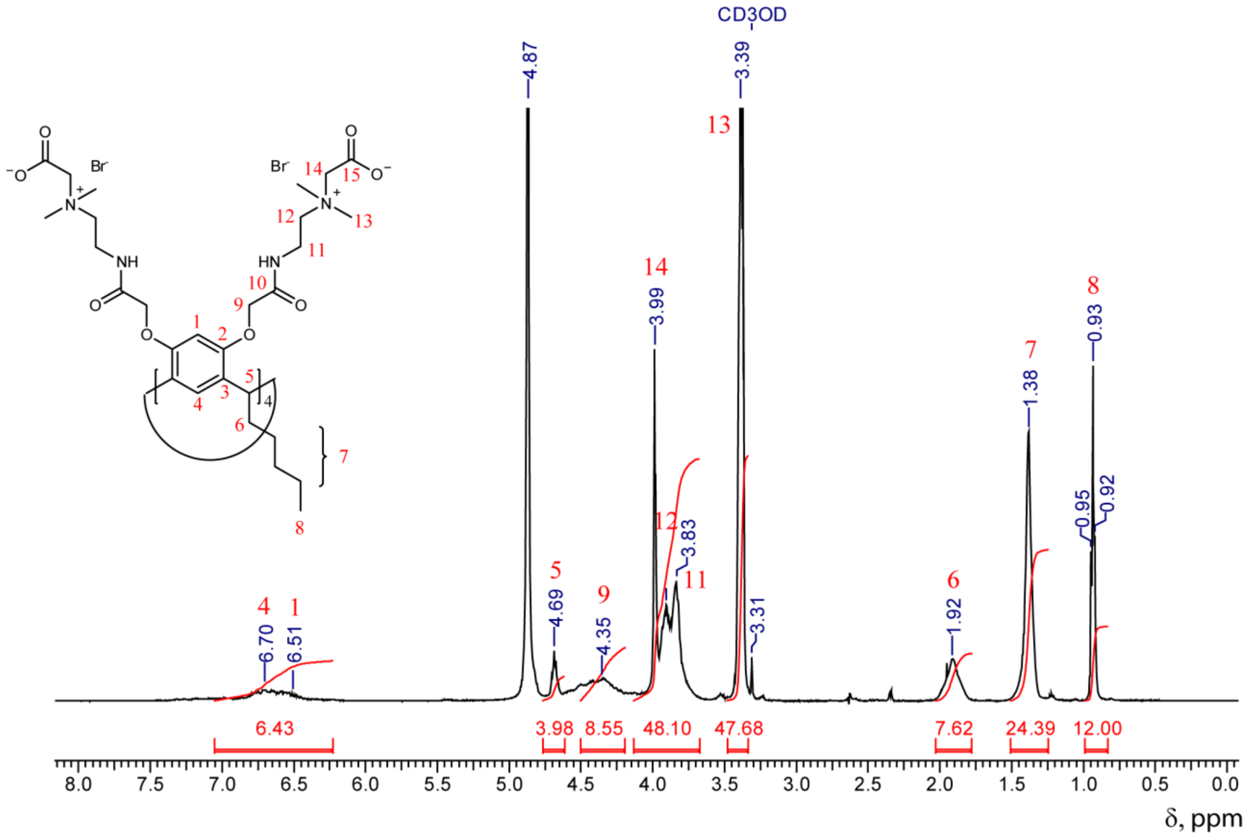

Figure S26.^{1}H NMR spectrum of macrocycle **5** in CD_3OD (303 K).

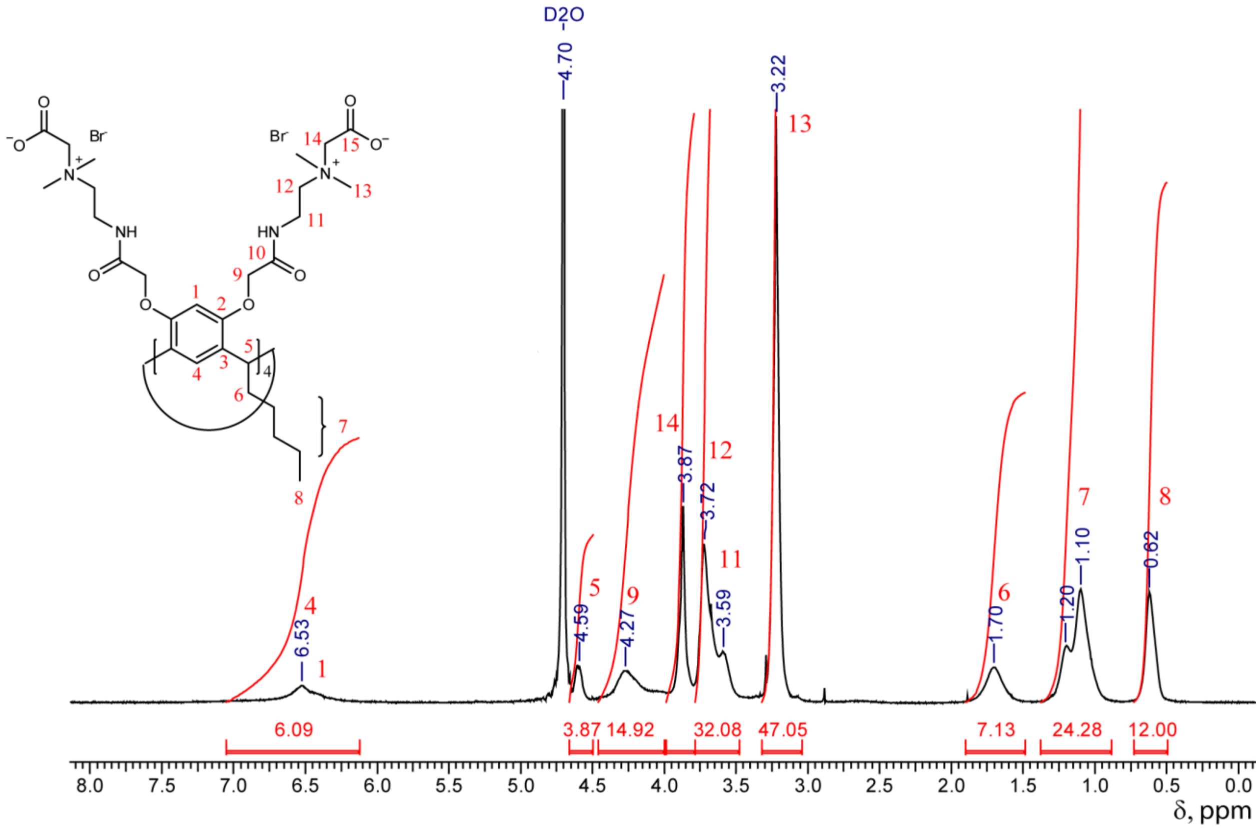

Figure S27.^{1}H NMR spectrum of macrocycle **5** in D_2O (303 K).

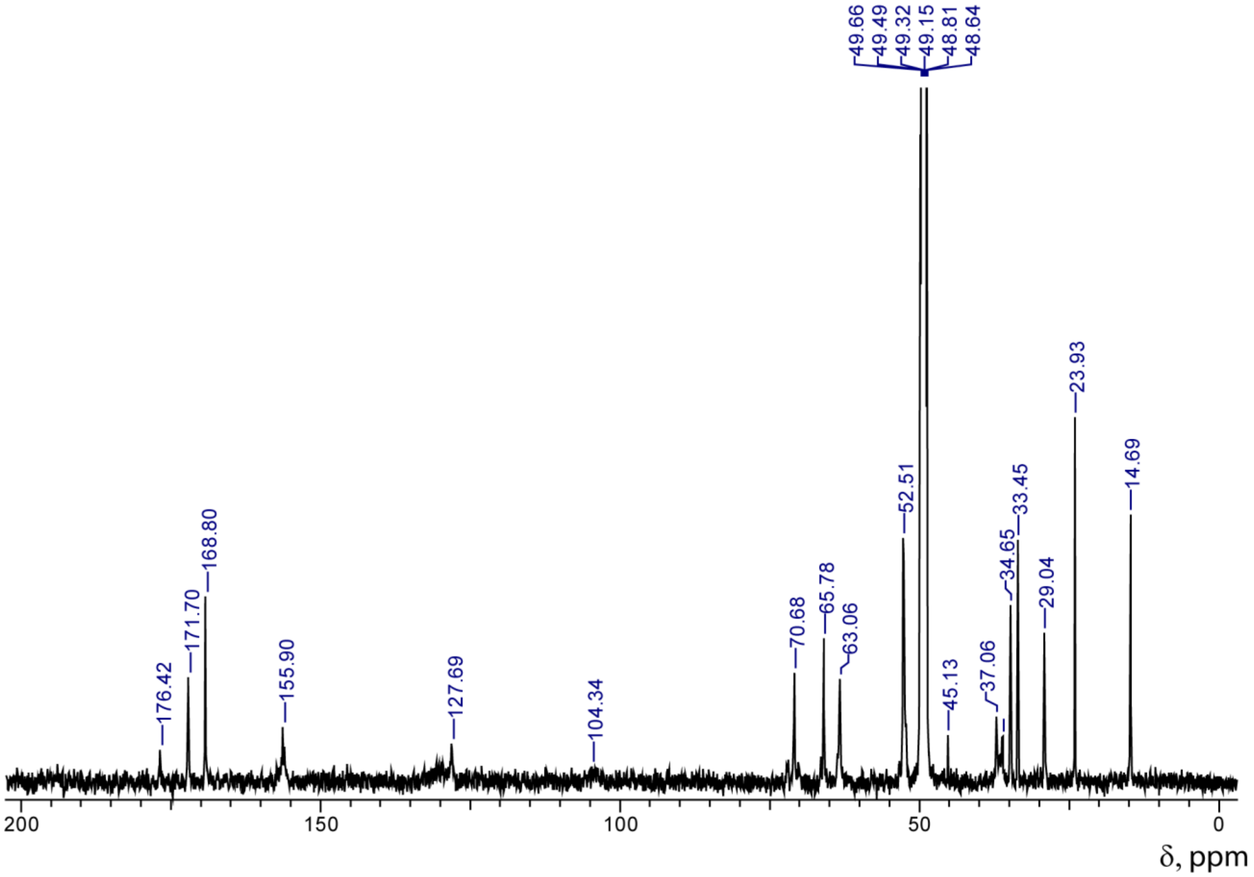

Figure S28. ^{13}C NMR spectrum of macrocycle **5** in CD_3OD (303 K).

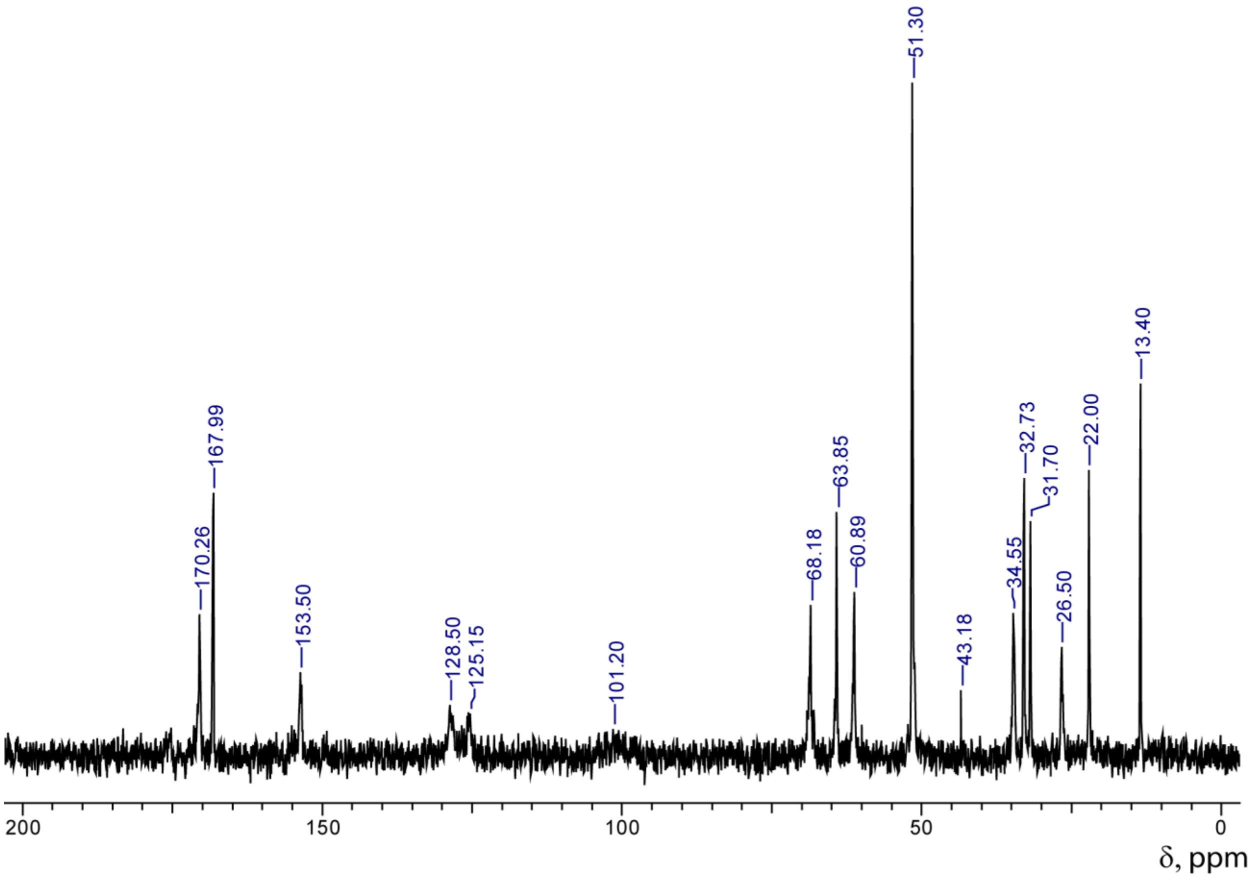

Figure S29. ^{13}C NMR spectrum of macrocycle **5** in D_2O (303 K).

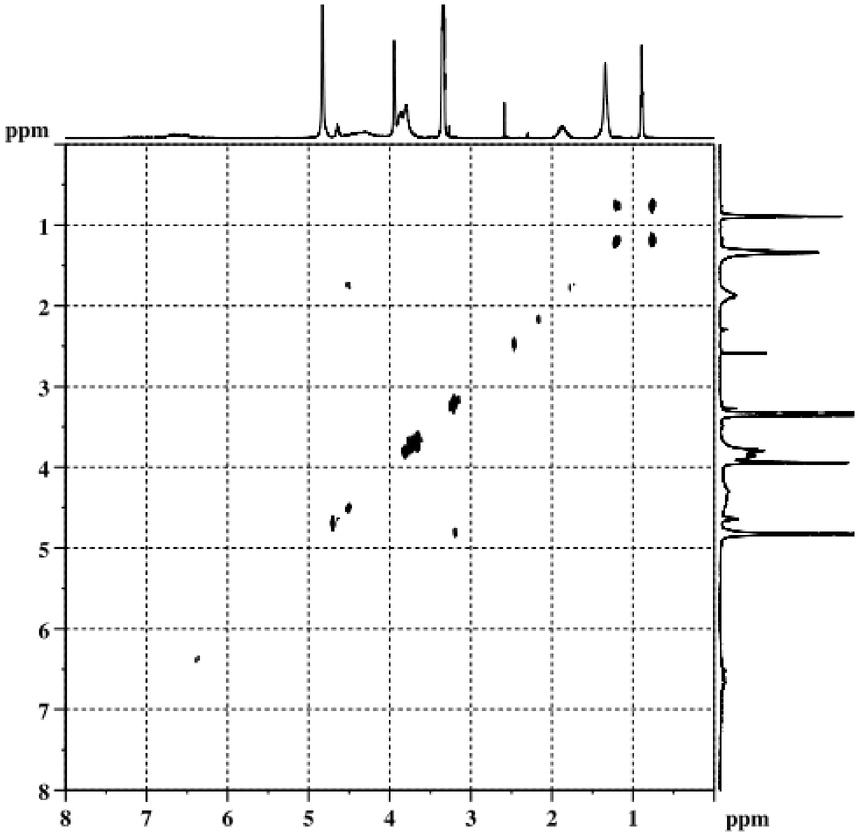

Figure S30. COSY spectrum of macrocycle **5** in CD_3OD (303 K).

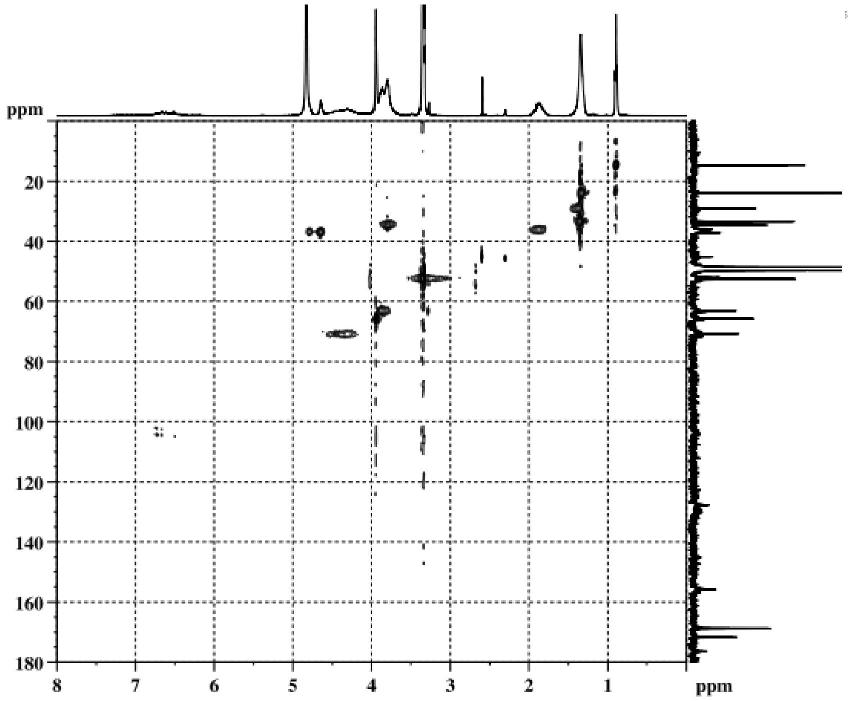

Figure S31. HSQC spectrum of macrocycle **5** in CD_3OD (303 K).

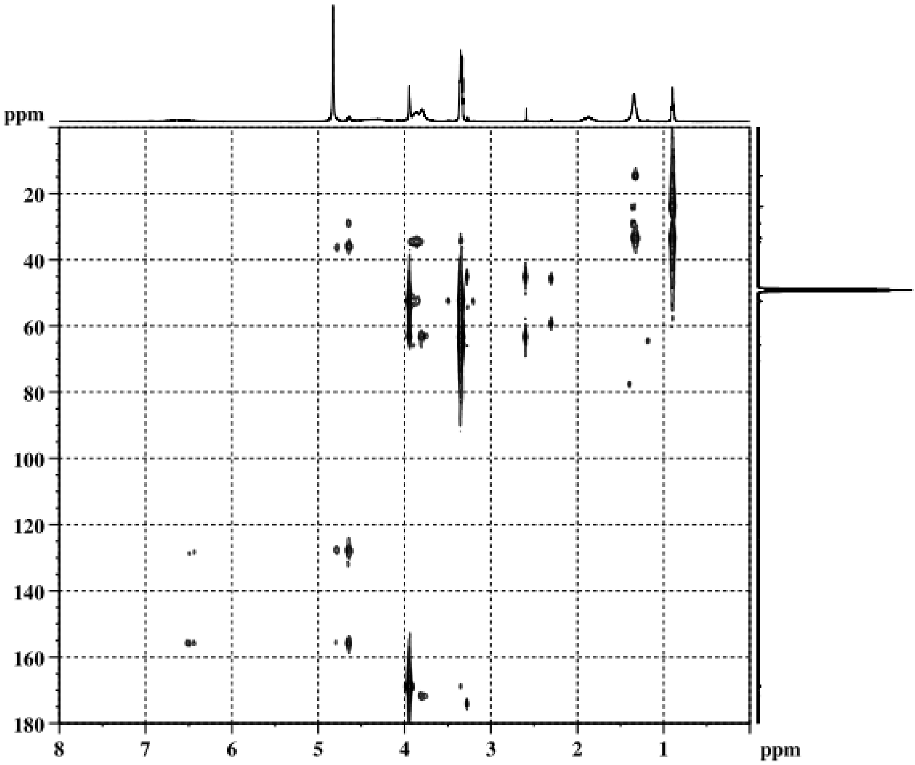

Figure S32. HMBC spectrum of macrocycle **5** in CD_3OD (303 K).

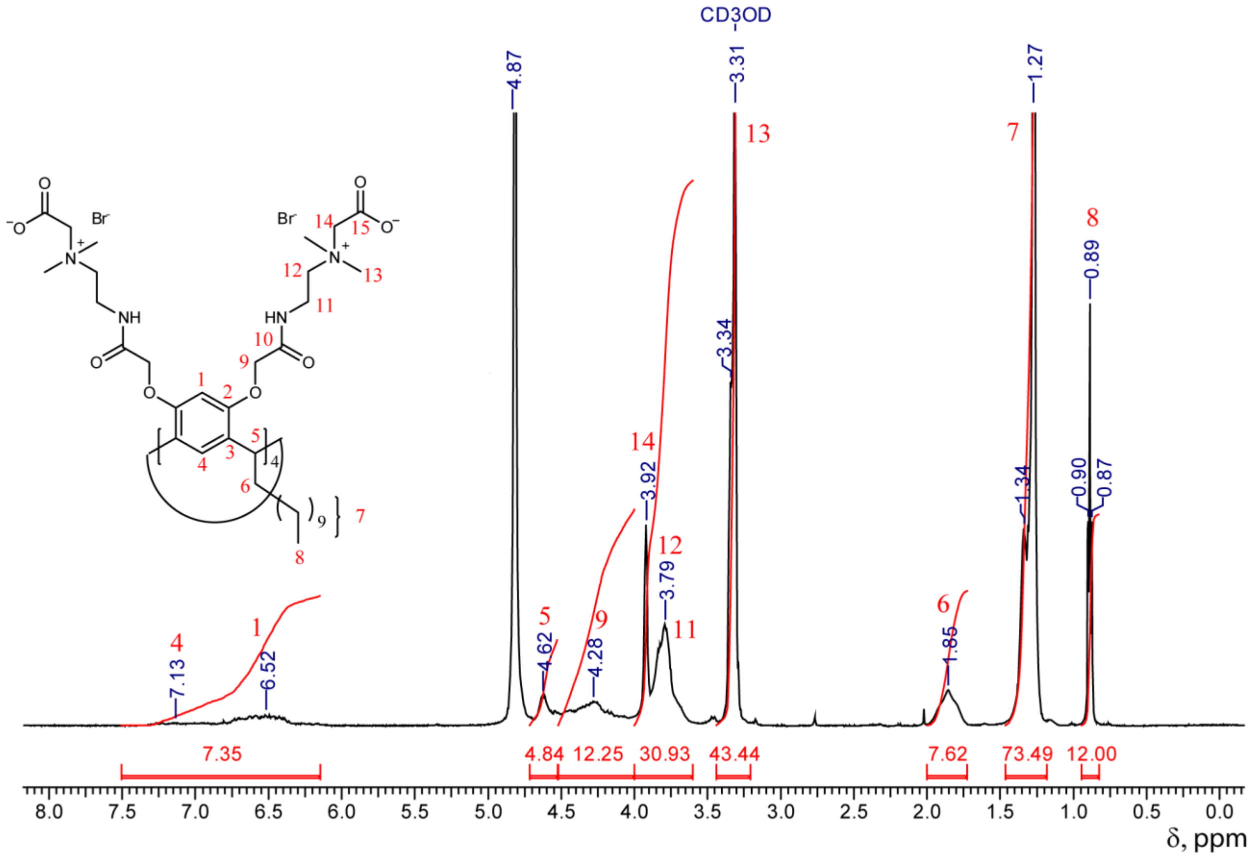

Figure S33.^{1}H NMR spectrum of macrocycle **6** in CD_3OD (303 K).

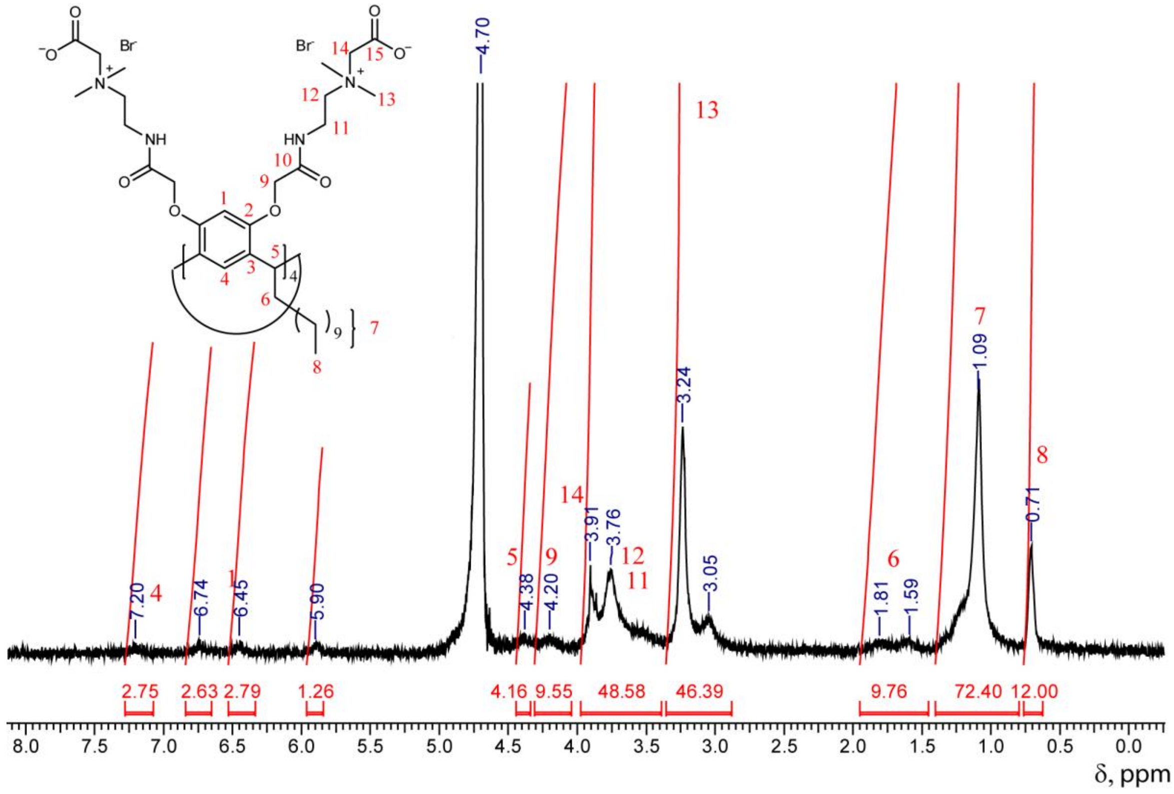

Figure S34.^{1}H NMR spectrum of macrocycle **6** in D_2O (303 K).

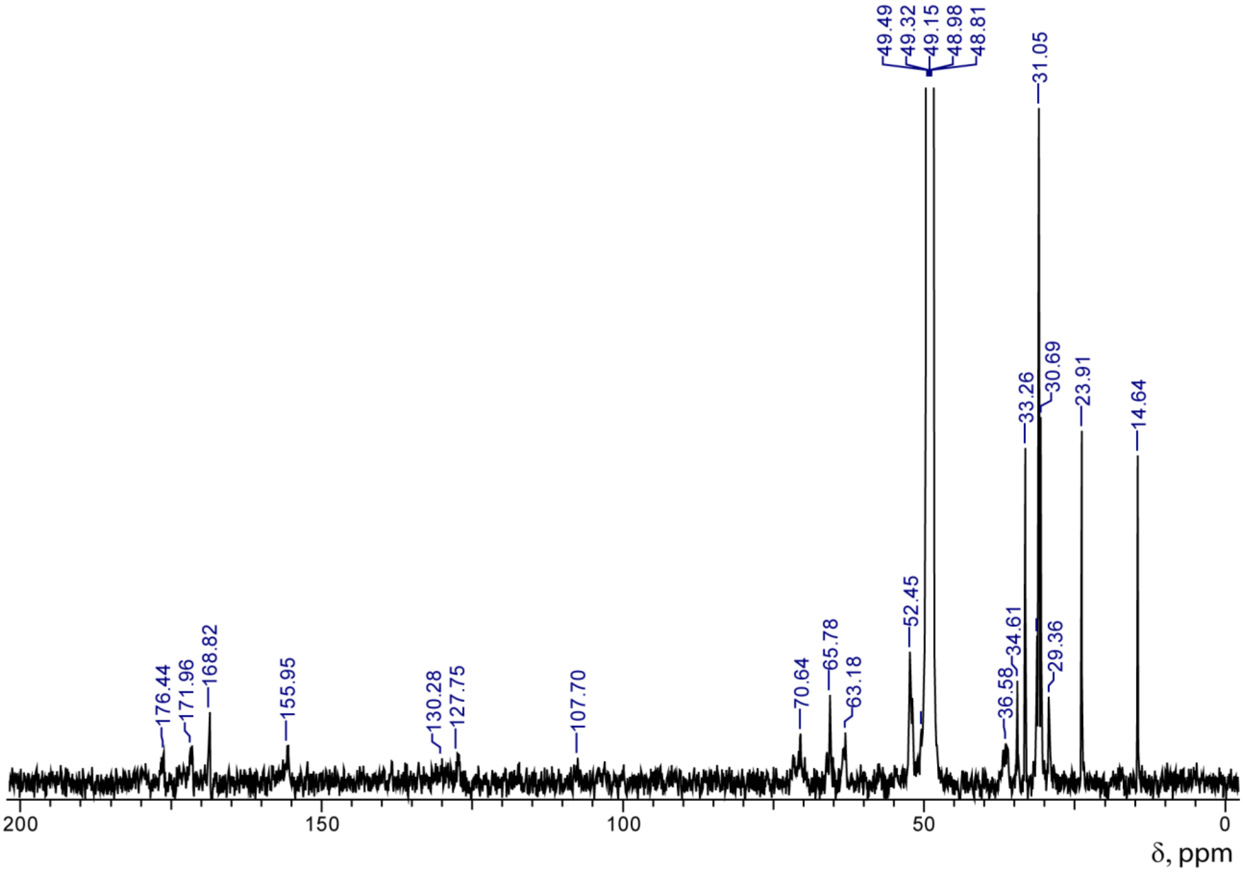

Figure S35. ^{13}C NMR spectrum of macrocycle **6** in CD_3OD (303 K).

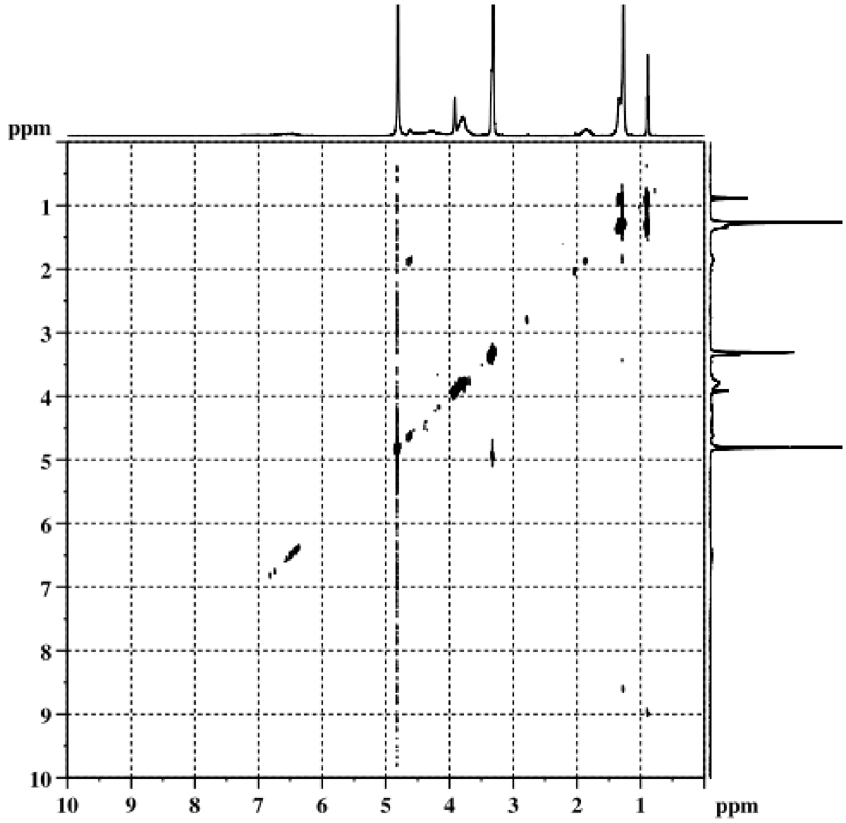

Figure S36. COSY spectrum of macrocycle **6** in CD_3OD (303 K).

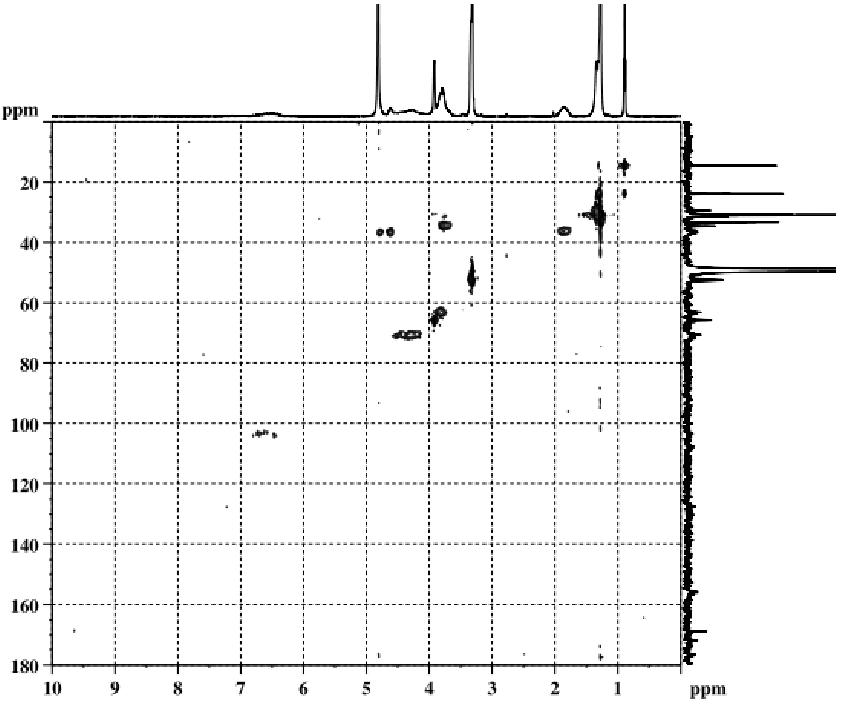

Figure S37. HSQC spectrum of macrocycle **6** in CD_3OD (303 K).

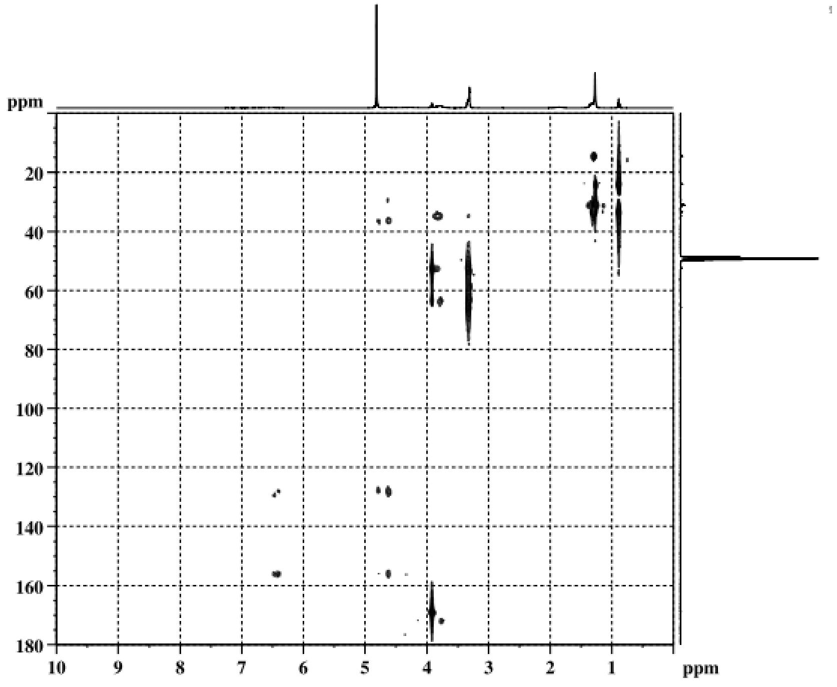

Figure S38. HMBC spectrum of macrocycle **6** in CD_3OD (303 K).

IR and ESI spectra of compounds 3-6

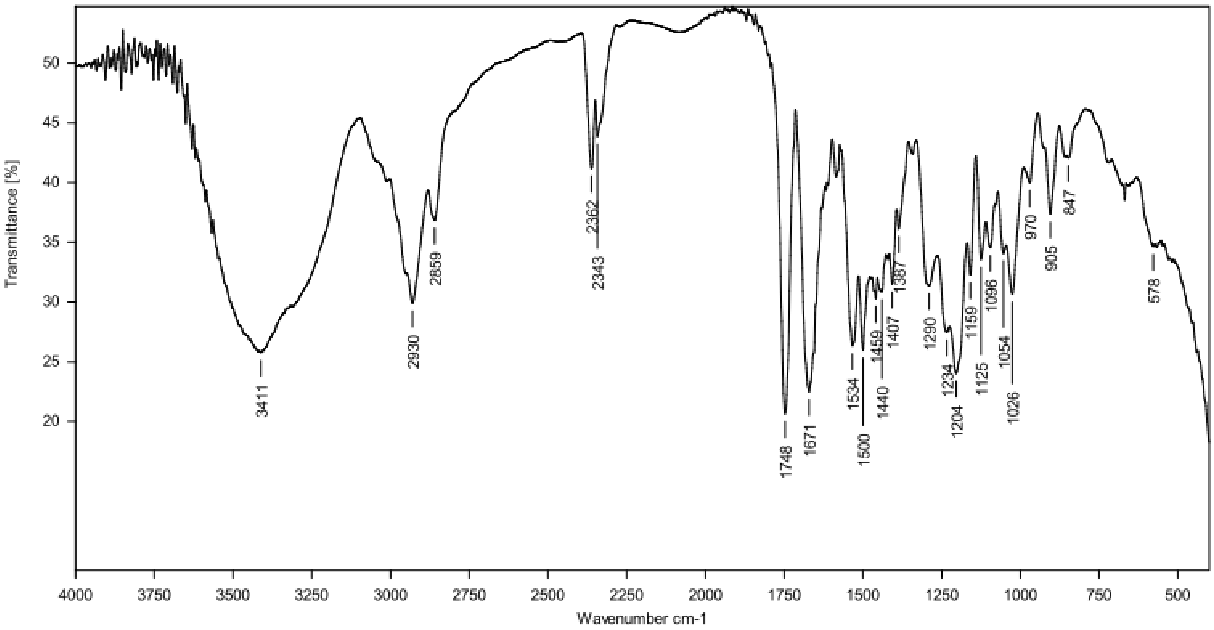

Figure S39. IR spectrum of macrocycle **3** (KBr).

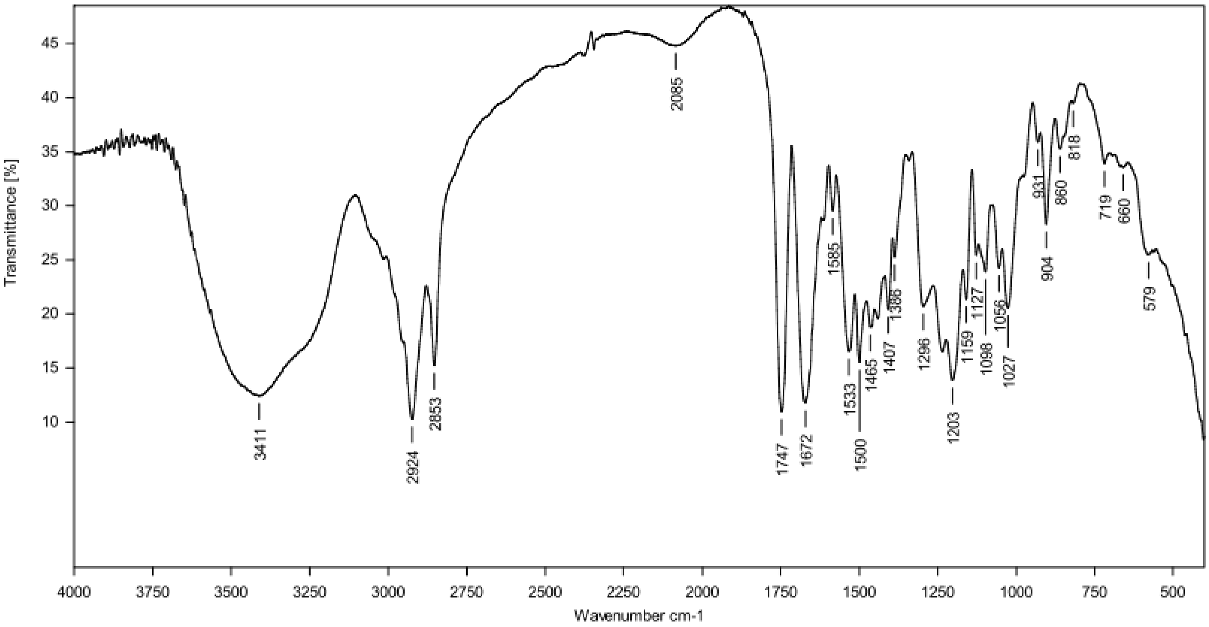

Figure S40. IR spectrum of macrocycle **4** (KBr).

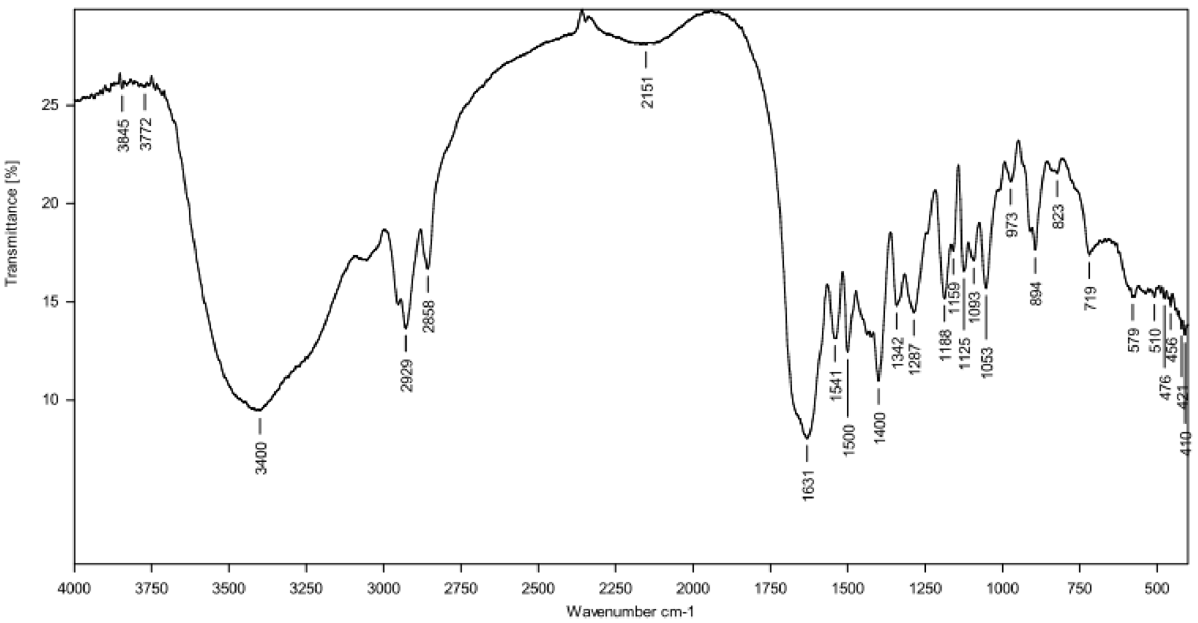

Figure S41. IR spectrum of macrocycle **5** (KBr).

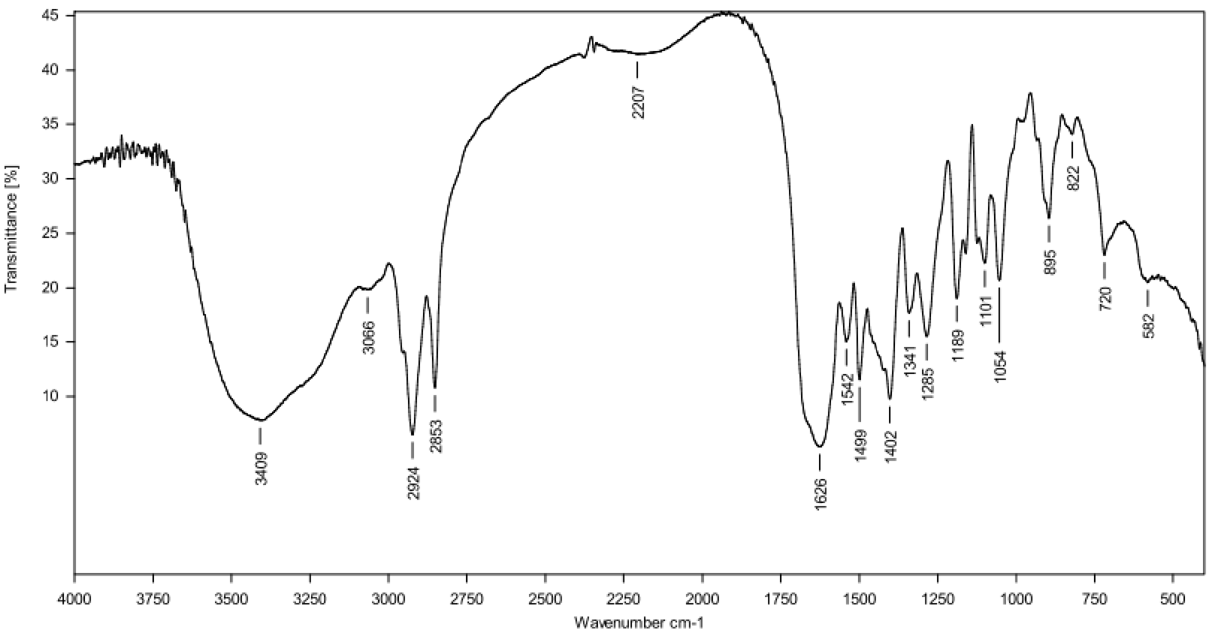

Figure S42. IR spectrum of macrocycle **6** (KBr).

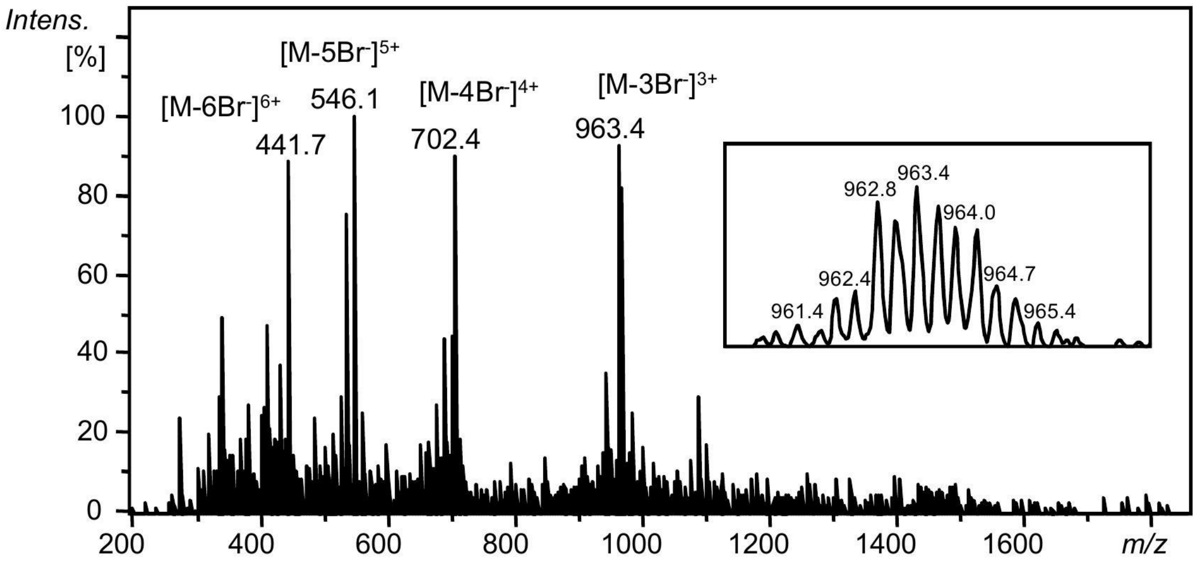

Figure S43. ESI mass spectrum of **3**.

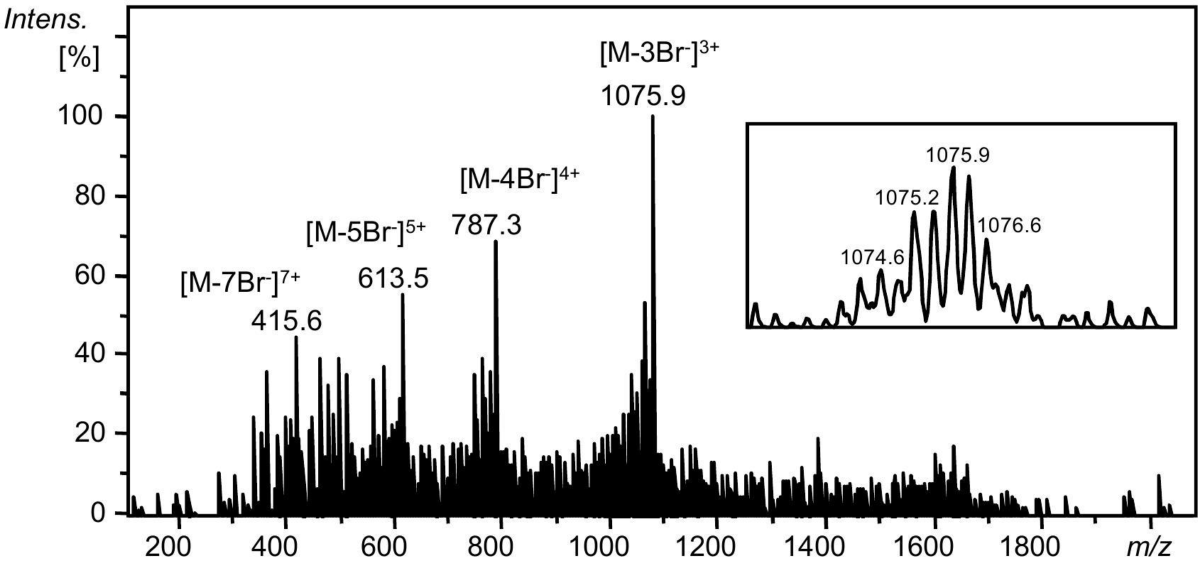

Figure S44. ESI mass spectrum of **4**.

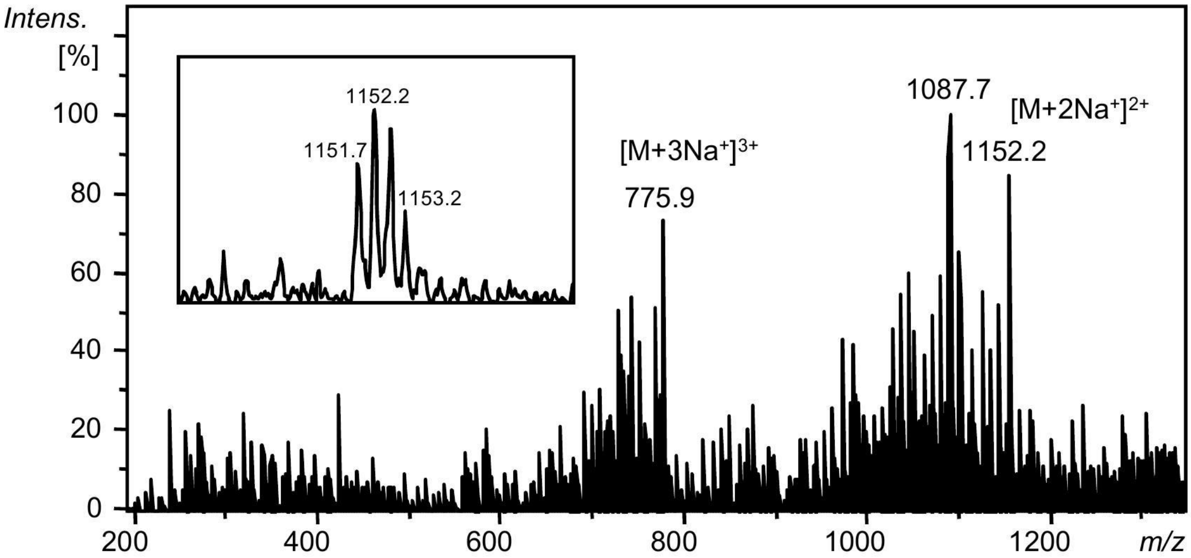

Figure S45. ESI mass spectrum of **5**.

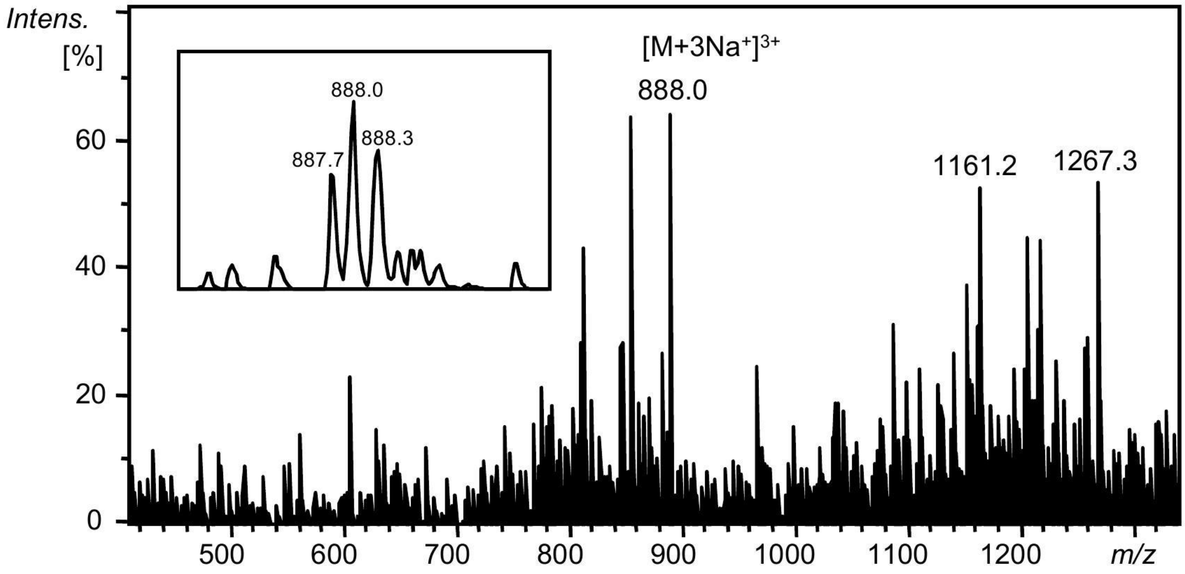

Figure S46. ESI mass spectrum of **6**.

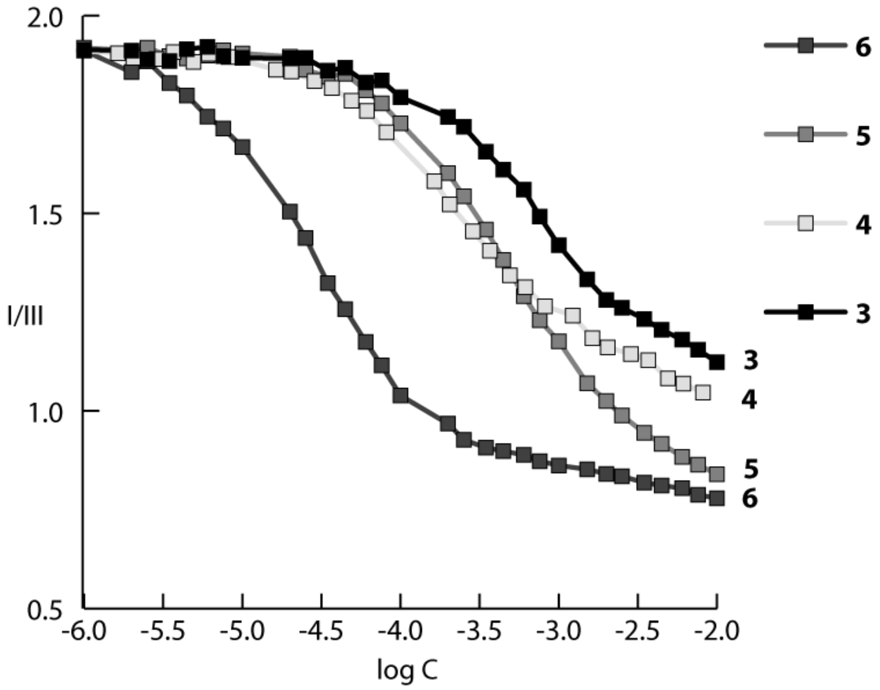

Figure S47. The pyrene I/III values dependence on the logarithmic concentration of macrocycles **3-6** in an aqueous solution, 25 °C

Cytotoxicity and hemolytic activity of 3 – 6

Table S1. Cytotoxicity of compounds **3** – **6** on Chang liver cell and M-HeLa cells

Test compound	Concentrations (mM)	Chang liver		M-HeLa	
		Cell viability (%)	IC_{50}(μM)	Cell viability (%)	IC_{50} (μM)
**3**	5.0	7.13	688	0	1244
	2.5	10.4		4	
	1.25	26.48		49.8	
	0.625	55.02		92.3	
	0.313	71.46		96.7	
	0.156	81.86		100	
**4**	5.0	2.3	1219	0	2166
	2.5	10.0		13.6	
	1.25	57.0		100	
	0.625	68.6		100	
	0.313	88.3		100	
	0.156	93.3		100	
**5**	5	59.9	6000	44.5	4600
	2.5	86.3		81.8	
	1.25	96.2		95.0	
	0.625	100		100	
	0.313	100		100	
**6**	5	45.2	3200	31.3	2400
	2.5	54.8		52.2	
	1.25	72.7		56.4	
	0.625	91.9		66.0	
	0.313	100		71.5	

Table S2. Hemolytic activity of compounds **3 - 6**

Test compound	Concentration (mM)	Hemolysis (%)	HC_{50} (μM)
**3**	5	2.9	>5000
	2.5	1.5	
	1.25	0.2	
	0.625	0	
	0.313	0	
**4**	5	8.0	>5000
	2.5	6.5	
	1.25	2.4	
	0.625	1.5	
	0.313	0	
**5**	5	1.4	>5000
	2.5	0.6	
	1.3	0	
	0.6	0	
**6**	5	0	>5000
	2.5	0	
	1.3	0	
	0.6	0	

The fluorescence and absorbance spectra of BSA – compound 3-6 solutions.

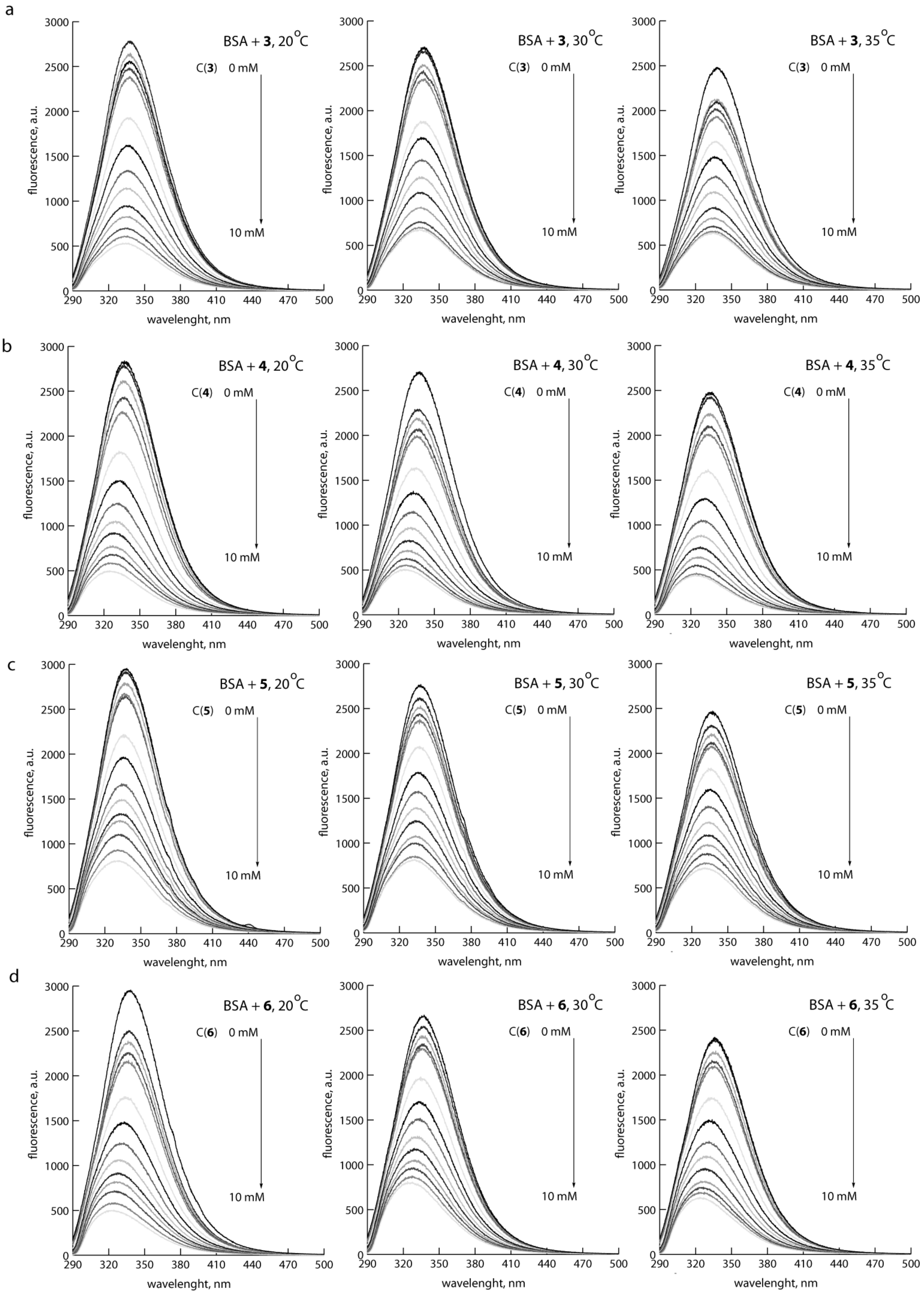

Figure S48. Fluorescence spectra of BSA solutions in the presence of macrocycle **3** (a), **4** (b), **5** (c), and **6** (d), at 20, 30, and 35 °C.

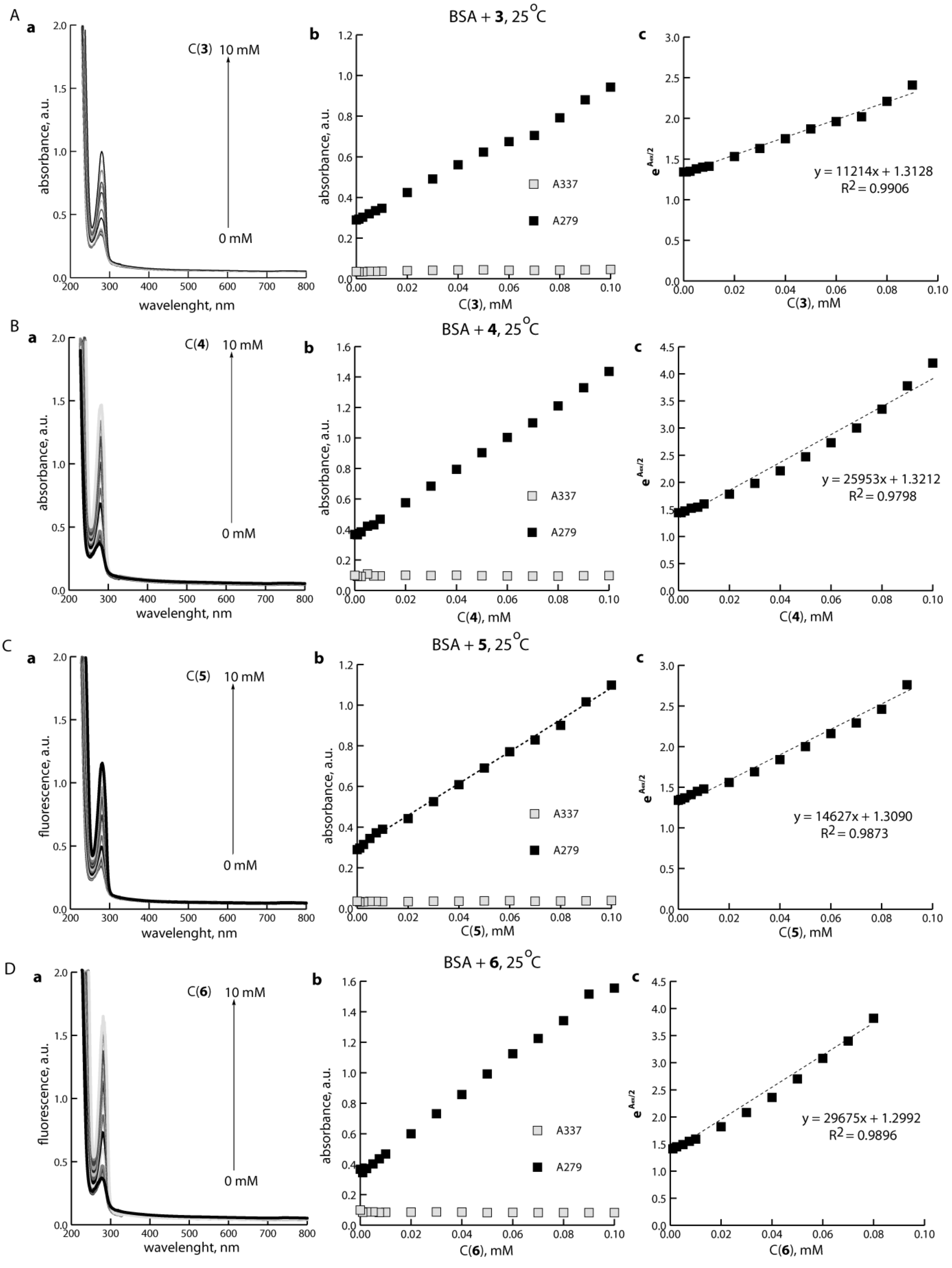

Figure S49. Absorbance spectra of BSA-macrocycles solutions (a), the dependence of the A^{337} and A^{279} on macrocycle' concentration C (b), the dependence of $e^{Aex/2}$ on macrocycle' concentration C (c) in the case of macrocycle **3** (A), **4** (B), **5** (C), and **6** (D), 25°C.

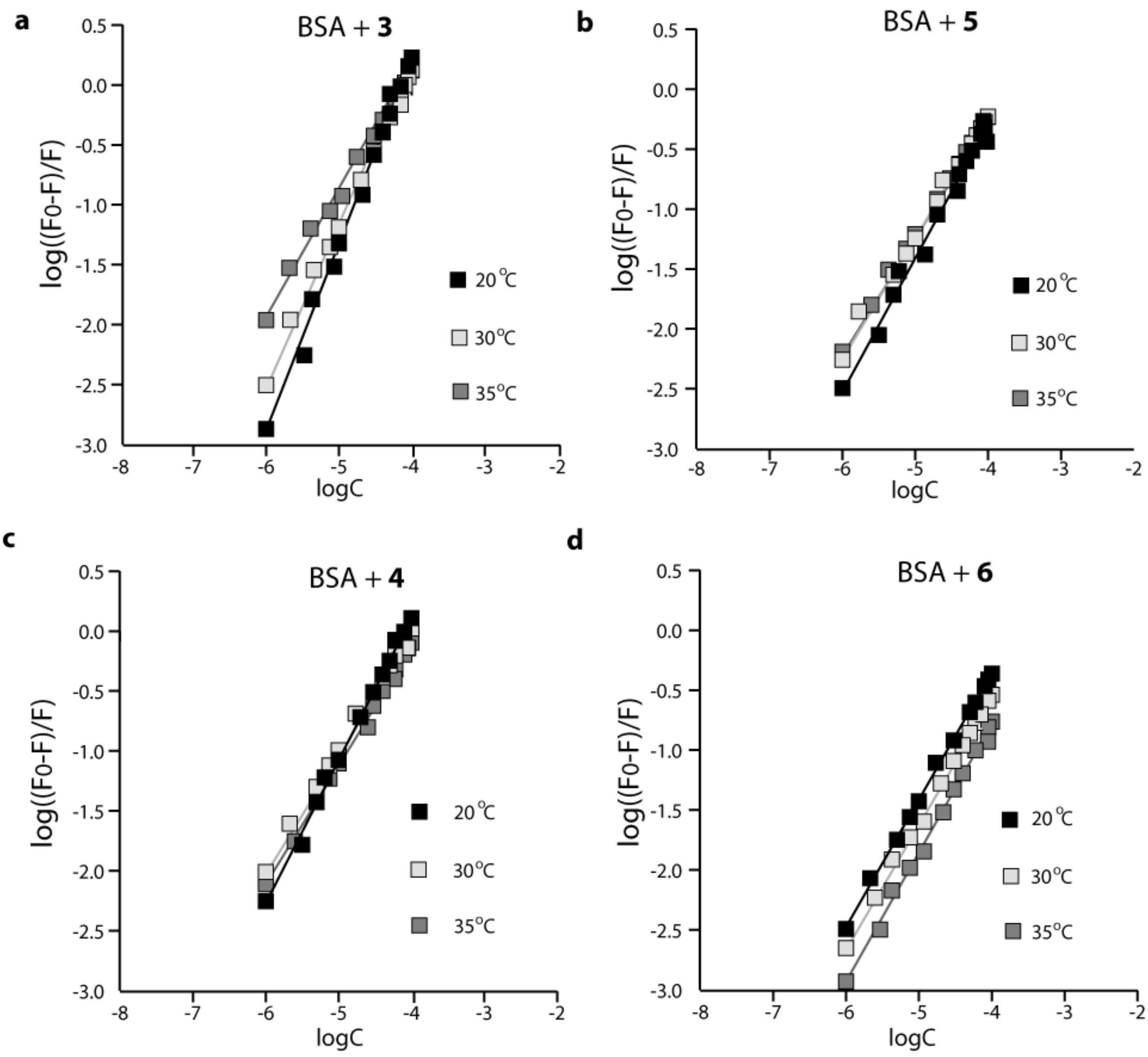

Figure S50. The dependence of log(F_0-F)/F on log[C] for BSA-macrocycles solutions for macrocycle **3** (a), **5** (b), **4** (c), and **6** (d) at 20, 30, and 35 °C.

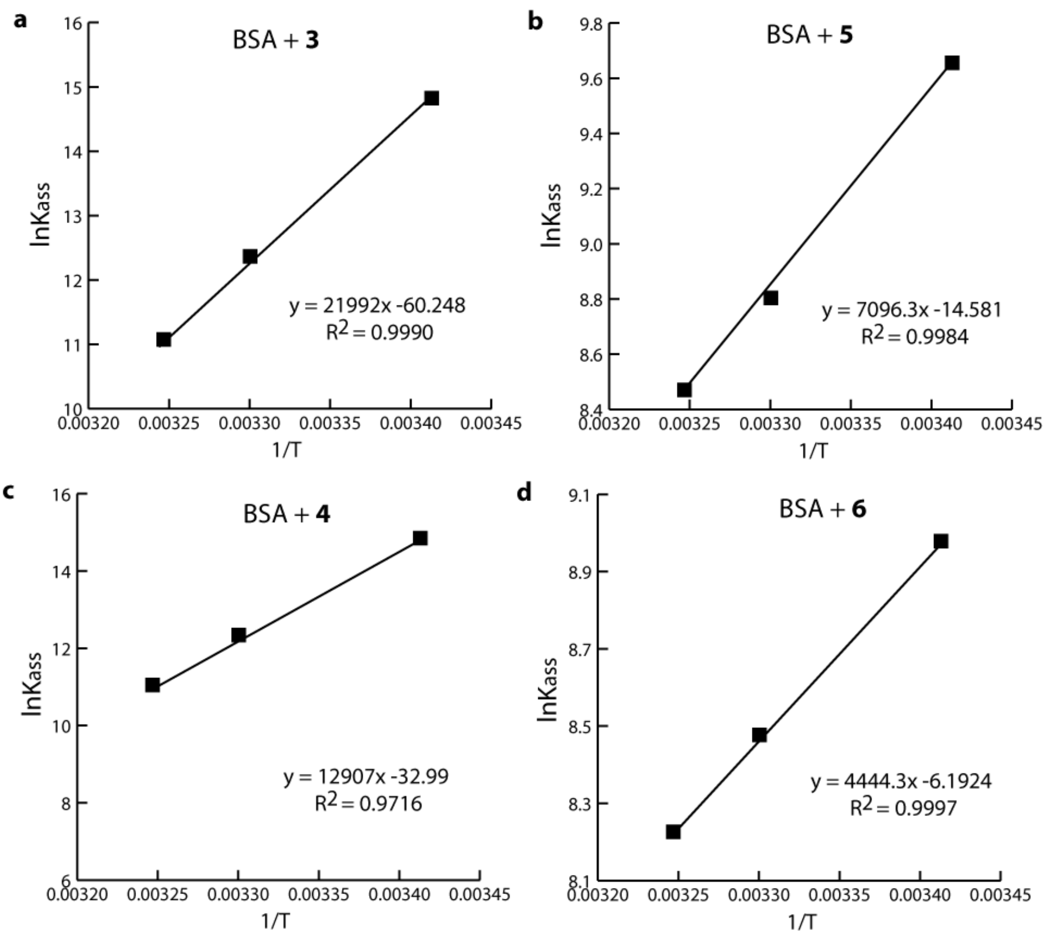

Figure S51. Van't Hoff plots for BSA binding by macrocycle **3** (a), **5** (b), **4** (c), and **6** (d).

Table S3. DLS data for BSA (0.01 mM), macrocycles, and BSA-macrocycles solutions in PB, pH 7, 25 °C.

	C(3-6), mM	fresh			after 2 week		
		d_h, nm		PDI	d_h, nm		PDI
		**number-averaged**	**intensity-averaged**		**number-averaged**	**intensity-averaged**	
BSA	-	10.1 ± 4.8	18.18; 37.84	0.323 ± 0.131	712.4 ± 6.63	825.0	0.219 ± 0.006
BSA+**3**	0.1	6.5 ± 3.4	28.21; 255.0	0.334 ± 0.045	precipitation	-	-
	1	13.5 ± 3.2	18.17; 190.1	0.408 ± 0.068	5.6 ± 3.0	6.503; 28.21; 342.0	0.877 ± 0.115
BSA+**4**	0.1	7.5 ± 3.9	32.67; 295.3	0.484 ± 0.188	precipitation	-	-
	1	4.9 ± 1.8	5.615; 24.36; 220.2	0.355 ± 0.045	4.2 ± 1.5	6.503; 28.21; 615.1	0.543 ± 0.129
BSA+**5**	0.1	7.5 ± 2.6	10.10; 37.84; 295.3	0.439 ± 0.135	7.5 ± 2.6	8.721; 21.04; 396.1	0.701 ± 0.262
	1	4.2 ± 1.6	4.849; 21.04; 164.2	0.279 ± 0.068	11.7 ± 5.2	13.54; 396.1	0.786 ± 0.239
BSA+**6**	0.1	4.9 ± 1.3	37.84; 396.1	0.361 ± 0.036	7.5 ± 2.6	21.04; 295.3	0.586 ± 0.113
	1	4.2 ± 1.5	6.503; 28.21; 122.4	0.405 ± 0.296	4.9 ± 1.3	6.503; 32.67; 458.7	0.620 ± 0.006
**3**	1	2.3 ± 0.6	2.696; 190.1	0.629	-	-	-
**4**	1	4.2 ± 1.0	4.849; 295.3	0.392	-	-	-
**5**	1	2.7 ± 0.7	3.615; 164.2	0.781	-	-	-
**6**	1	4.2 ± 1.1	4.849; 105.7	0.477	-	-	-

References

1. Linda M. Tunstad, John A. Tucker, Enrico Dalcanale, Jurgen Weiser, Judi A. Bryant, John C. Sherman, Roger C. Helgeson, Carolyn B. Knobler, Donald J. Cram. Host-Guest Complexation. 48. Octol Building Blocks for Cavitands and Carcerands. *J. Org. Chem.* 1989, *54*, 1305-1312.
